# Systematics and diversification of *Anindobothrium* Marques, Brooks & Lasso, 2001 (Eucestoda: Rhinebothriidea)

**DOI:** 10.1371/journal.pone.0184632

**Published:** 2017-09-27

**Authors:** Bruna Trevisan, Juliana F. Primon, Fernando P. L. Marques

**Affiliations:** 1 Curso de Pós-graduação/Instituto de Biociências, Universidade de São Paulo, São Paulo, São Paulo, Brazil; 2 Departamento de Zoologia/Instituto de Biociências, Universidade de São Paulo, São Paulo, São Paulo, Brazil; University of Minnesota, UNITED STATES

## Abstract

Tapeworms of the genus *Anindobothrium* Marques, Brooks & Lasso, 2001 are found in both marine and Neotropical freshwater stingrays of the family Potamotrygonidae. The patterns of host association within the genus support the most recent hypothesis about the history of diversification of potamotrygonids, which suggests that the ancestor of freshwater lineages of the Potamotrygonidae colonized South American river systems through marine incursion events. Despite the relevance of the genus *Anindobothrium* to understand the history of colonization and diversification of potamotrygonids, no additional efforts were done to better investigate the phylogenetic relationship of this taxon with other lineages of cestodes since its erection. This study is a result of recent collecting efforts to sample members of the genus in marine and freshwater potamotrygonids that enabled the most extensive documentation of the fauna of *Anindobothrium* parasitizing species of *Styracura* de Carvalho, Loboda & da Silva, *Potamotrygon schroederi* Fernández-Yépez, *P. orbignyi* (Castelnau) and *P. yepezi* Castex & Castello from six different countries, representing the eastern Pacific Ocean, Caribbean Sea, and river basins in South America (Rio Negro, Orinoco, and Maracaibo). The newly collected material provided additional specimens for morphological studies and molecular samples for subsequent phylogenetic analyses that allowed us to address the phylogenetic position of *Anindobothrium* and provide molecular and morphological evidence to recognize two additional species for the genus. The taxonomic actions that followed our analyses included the proposition of a new family, Anindobothriidae fam. n., to accommodate the genus *Anindobothrium* in the order Rhinebothriidea Healy, Caira, Jensen, Webster & Littlewood, 2009 and the description of two new species—one from the eastern Pacific Ocean, *A. carrioni* sp. n., and the other from the Caribbean Sea, *A. inexpectatum* sp. n. In addition, we also present a redescription of the type species of the genus, *A. anacolum* (Brooks, 1977) Marques, Brooks & Lasso, 2001, and of *A. lisae* Marques, Brooks & Lasso, 2001. Finally, we discuss the paleogeographical events mostly linked with the diversification of the genus and the protocols adopted to uncover cryptic diversity in *Anindobothrium*.

## Introduction

Members of the genus *Anindobothrium* Marques, Brooks & Lasso, 2001 are found in both marine and Neotropical freshwater stingrays of the family Potamotrygonidae. This genus was erected to accommodate the marine species *Caulobothrium anacolum* Brooks, 1977, a parasite of *Styracura schmardae* (Werner) de Carvalho, Loboda & da Silva off the Caribbean coast of Colombia, and two species found in freshwater potamotrygonids: *A. lisae* Marques, Brooks & Lasso, 2001 and *A. guariticus* Marques, Brooks & Lasso, 2001. Subsequently, Reyda [1] transferred *A. guariticus* to his newly erected genus *Nandocestus* Reyda, 2008. Therefore, *Anindobothrium* is now comprised of two valid species: its type, *A. anacolum* (Brooks, 1977) Marques, Brooks & Lasso, 2001, a parasite of *S. schmardae* collected off the Caribbean coast of Colombia and *A. lisae*, a parasite of freshwater stingray *Potamotrygon orbignyi* (Castelnau) from the Rio Negro, near Barcelos-Amazonas (Brazil).

At present, the systematic position of *Anindobothrium* is considered uncertain [2, 3]. Marques *et al*. [4] erected the genus as a member of the Phyllobothriidae Braun, 1900 [5], a family of the notoriously polyphyletic order Tetraphyllidea Carus, 1863 [6]. In the past decade, the disassembly of the Tetraphyllidea has led to extensive modifications [2, 3, 7, 8] of the concept of the family Phyllobothriidae. Throughout the recent rearrangement of former tetraphyllidean taxa and redefinition of the Phyllobothriidae, Healy *et al*. [2] and Ruhnke *et al*. [9] have suggested that *Anindobothrium* is likely to be a member of the Rhinebothriidea Healy, Caira, Jensen, Webster & Littlewood, 2009, since its members possess stalked bothridia—a putative morphological synapomorphy for the order, which concept was mainly supported by the phylogenetic analysis of molecular data [2, 8, 10]. Since those initial propositions, no effort has been made to address the phylogenetic position of *Anindobothrium* and test the hypothesis that the genus is a member of the Rhinebothriidea.

The observation that *Anindobothrium* is comprised of one member from Neotropical freshwater potamotrygonids and another found in *S. schmardae* from the Caribbean Sea supports the most recent hypothesis about the origin of freshwater stingrays [11–16]. There is evidence to suggest that a freshwater potamotrygonid ancestor colonized South America through marine incursion events during the Paleogene Period, between the early Miocene and mid-Eocene (*i.e.*, 22.5–46 Mya) [12, 14, 15, 17]. According to this hypothesis the current tropical eastern Pacific Ocean and the Caribbean Sea are the areas of derivation of freshwater potamotrygonids. This biogeographic scenario is corroborated by recent phylogenetic hypotheses for batoids based on morphological and molecular data, suggesting that species of *Styracura* de Carvalho, Loboda & da Silva—*S. schmardae* and *S. pacifica* (Beebe & Tee-Van) de Carvalho, Loboda & da Silva—form a clade sister to Neotropical freshwater stingrays [11, 13, 16, 18].

In historical associations between hosts and parasites, if events of co-divergence prevail, we would expect to find congruence between patterns of diversification for host and parasite phylogenies [19]. The same would apply for historical associations between areas and organisms in which vicariance events would render sister taxa inhabiting sister areas. This theoretical framework generates two expectations: (1) that freshwater lineages of *Anindobothrium* inhabiting freshwater potamotrygonids would be closely related to those found in species of *Styracura* and (2) that *S. pacifica* should house at least one lineage of *Anindobothrium*.

Here, we address the phylogenetic position of *Anindobothrium* and the diversity of the genus in species of potamotrygonid hosts. The phylogenetic position of the genus is discussed in light of a phylogenetic analysis of molecular data. The taxon sampling includes members of *Anindobothrium* from both marine and freshwater systems in addition to species from other cestode genera representing all major lineages of the Rhinebothriidea as well as other orders of cestodes. The diversity of *Anindobothrium* reported here is the result of the analyses of molecular and morphological data from an unprecedented number of specimens obtained after examining over 152 freshwater and 33 marine potamotrigonid hosts. Finally, we provide the description of two new species and a redescription of *Anindobothrium anacolum*, the type species of the genus, and *A. lisae*, to include new data on morphological variation and characterization of tegumentary structures (*i.e.*, microtriches).

## Materials and methods

### Biological material

A total of 92 specimens of *Potamotrygon orbignyi* and 39 specimens of *P. schroederi* Fernández-Yépez from the Rio Negro and Rio Orinoco, respectively, 21 specimens of *P. yepezi* Castex & Castello from the Maracaibo basin, and 33 specimens of *Styracura* spp. from the eastern Pacific Ocean and the Caribbean Sea were examined in this study. Freshwater specimens were collected using long-lines or hand nets, whereas marine ones were collected by spears. Collecting activities took place in six different countries and followed the guidelines of collecting permits issued to F.P.L. Marques by IBAMA no. 006/96-DIFAS of January, 1996; no. 015/04 of January, 2004; 083/05-DIFAS of July, 2005; and 24451-1 of July, 2010 in Brazil; by the Instituto Socialista de la Pesca y Agricultura—INSOPESCA AMAZONAS no. 038 in Venezuela; by the Ministery of Food Production—Fisheries Division (issued in September, 2014) in Trinidad & Tobago; by the Autoridad Nacional del Ambiente—ANAM (SE/A-101-14, issued in December, 2014) in Panama; and to J.N. Caira by INDERENA Institute de Pesca y Fauna Terrestre from Bogotá in Colombia; and by the Ministry of Forest, Fisheries and Sustainable Development (Belize Fisheries Department—Proc. No 000016-12, issued in 2012) in Belize. Details on the hosts examined in the present study are in the supporting information (S1 Text).

The spiral intestines of hosts were removed and opened with a longitudinal incision. Marine hosts were fixed individually in 10% formalin buffered in seawater (1:9) or 95% ethanol and shaken for approximately two minutes. Freshwater hosts were fixed likewise with the exception of the use of 10% formalin diluted in saline solution. Samples fixed in ethanol were stored at -20°C; those fixed in formalin were transferred to 70% ethanol after approximately one week for long-term storage. Parasite specimens included in this study were selected from the spiral intestines using a stereomicroscope.

### Morphological data

Cestode specimens prepared as whole mounts for light microscopy were hydrated, stained with Delafield’s hematoxylin (9:1 solution), destained in a 1% acid (HCl) ethanol solution, followed by a 1% basic (NaOH) ethanol solution, dehydrated in ethanol series to 100%, cleared in methyl salicylate and mounted in Canada balsam on glass slides under coverslips. Selected specimens were dehydrated in ethanol series to 95%, stained with acetocarmine, dehydrated in 100% ethanol, and cleared and mounted as described above.

Histological sections were prepared from terminal proglottids of at least one specimen per population (*i.e.*, from each host and locality). Anterior parts of the worms were removed and prepared as whole mounts as described above to serve as vouchers. Posterior parts of the strobila were embedded in paraffin according to conventional techniques. Sections were prepared at 7 *μ*m intervals, using a Leica RM 2025 retracting rotary microtome, placed on glass slides, allowed to dry on a slide warmer for 5 min and then in an oven for 30 min, stained in Mayer’s hematoxylin, counterstained in eosin, dehydrated in ethanol series to 100%, cleared in xylene, and mounted in Canada balsam on glass slides under coverslips.

Scoleces selected for scanning electron microscopy (SEM) were carefully cleaned with brushes to remove the host tissue and mucus, hydrated, transferred to 1% osmium tetroxide overnight, dehydrated in ethanol series until 100%, placed in hexamethyldisilizane (HMDS), allowed to air-dry overnight and mounted on carbon tape on an aluminum stub. The stubs were sputter-coated with gold/palladium and examined with a Zeiss DCM 940 and FEI Quanta 600 FEG scanning electron microscope. The strobilae of the worms used for SEM were prepared as whole mounts as described above to serve as vouchers.

Line drawings were prepared with the aid of a drawing tube attached to a Zeiss Axioscope 2. Whole mounts were photographed using either a Zeiss Axioscope 2 equipped with a SPOT digital camera or an Olympus BX51 equipped with an Olympus SC30 camera. Fiji/ImageJ [20] was used to process the images. Morphometric data were compiled with WormBox [21] and further summarized using a script in R [22]. Only complete specimens with mature (*i.e.*, with open genital pores) or further developed proglottids (*e.g.*, with atrophied testes or filled vas deferens) were examined and measured. All measurements for reproductive structures were taken from terminal proglottids, unless in cases terminal proglottids presented atrophied testes, in which measurements were taken from subterminal mature proglottids. All measurements are in micrometers unless otherwise stated, and are presented as ranges followed by the number of specimens from which each measure was taken in parentheses. Repeated measurements for the number and dimensions of testes and for the dimensions of vitelline follicles were averaged for individuals. Terminology for the shape of the bothridia follows Clopton [23]. Microtriches terminology follows Chervy [24].

Museum abbreviations are as follows: **CHIOC**, Coleção Helmintológica do Instituto Oswaldo Cruz, Rio de Janeiro, Rio de Janeiro, Brazil; **HWML**, Harold W. Manter Laboratory of Parasitology Collection, University of Nebrasca, Lincoln, Nebrasca, U.S.A.; **INPA**, Instituto Nacional de Pesquisas da Amazônia, Manaus, Amazonas, Brazil; **LRP**, Lawrence R. Penner Parasitology Collection, Department of Ecology and Evolutionary Biology, University of Connecticut, Storrs, Connecticut, U.S.A.; **MIUP**, Museo de Invertebrados G. B. Fairchild, Estafeta Universitaria, Universidad de Panamá, Panama; **MZUSP**, Museu de Zoologia da Universidade de São Paulo, São Paulo, Brazil; **USNM**, National Museum of Natural History, Smithsonian Institution, Washington, D.C., U.S.A.; and **USNPC**, United States National Parasite Collection, Beltsville, Maryland, U.S.A. (now available at the **USNM**).

### Molecular data acquisition

Scolices and posterior portions of strobila from specimens used in molecular analyses were prepared as whole mounts as described above. Hologenophores (sensu Pleijel *et al*. [25]) were deposited at the MZUSP. The middle portion of the strobila of each specimen was removed and allowed to air dry for about 5 minutes at room temperature. Total genomic DNA was extracted using Agencourt DNAdvance—Nucleic Acid Isolation Kit (Beckman Coulter) following manufacturer’s instructions. Genomic DNA was quantified using a micro-volume spectrophotometer, NanoDrop 2000 (Thermo Scientific). Polymerase Chain Reaction (PCR) was used to amplify partial sequences of nuclear regions: 18S rDNA, 28S rDNA (D1–D3), Calmodulin (Cal), and Internal Transcribed Spacer 1 (ITS-1), and the mitochondrial region of Cytochrome Oxidase I (COI). Amplifications were performed in a 25 *μ*l volume containing 1 *μ*l of DNA, 20 mM Tris-HCl (pH 8.4), 50 mM KCl, 200 uM dNTPs, 1.0–3.0 mM MgCl_2_, 0.4 uM of each primer, and 1 U of Taq DNA polymerase recombinant (Fermentas, Thermo Scientific). General PCR conditions included initial denaturation for 5 min at 95°C, 35 cycles of denaturation for 30 sec at 95°C, annealing for 30 sec at specific temperatures (see below), extension for 1 min to 1 min and 10 sec at 72°C, and a final extension for 7 min at 72°C. Amplifications and sequencing were performed with following primer sets: 18S rDNA with 300F 5’—AGG GTT CGA TTC CGG AG—3’ and WormB 5’—CTT GTT ACG ACT TTT ACT TCC—3’ at 55°C; 28S rDNA (D1–D3) with LSU-5F 5’—TAG GTC GAC CCG CTG AAY TTA AGC A—3’ and LSU-1500R 5’—GCT ATC CTG AGG GAA ACT TCG—3’ at 58°C; Cal with Cal F 5’—GAR CAR ATT GCI GAR TTY AAR GAR GC—3’ and Cal R 5’—TTC TTC RTA RTT IAC YTG ICC RTC—3’ with 5 cycles at 55°C followed by 5 cycles at 50°C; ITS-1 with CAS-18S F1 5’—TAC ACA CCG CCC GTC GCT ACT A—3’ and CAS-5,8S B1d 5’—TTC TTT TCC TCC SCT TAY TRA TAT GCT TAA CAS—3’ at 58°C; and COI with sean1 5’—TTT ACT TTG GAT CAT AAG CG—3’ and HCO 2198 5’—TAA ACT TCA GGG TGA CCA AAA AAT CA—3’ at 45°C. PCR products were purified using an Agencourt AMPure XP DNA Purification and Cleanup kit (Beckman Coulter Inc.). Products were subsequently cycle-sequenced directly from both forward and reverse directions using ABI Big-Dye Sequence Terminator (v. 3.1), cleaned with ethanol precipitation, and sequenced on an ABI Prism Genetic Analyzer (3131XL) automated sequencer (Applied Biosystems/ThermoFisher).

Contiguous sequences were assembled using the package Consed/PhredPhrap [26–29]. Sequences were aligned using MAFFT [30] and visualized and edited in BioEdit (v. 7.1.3.0; [31]) to remove leading and trailing regions that varied in length. Kimura’s two-parameter (**K2P**) distances among sequences were calculated in PAUP* [32]. Haplotype networks [33] were calculated based on uncorrelated distances from implied alignments (see below) using Pegas (version 0.9; Paradis [34]).

### Phylogenetic analyses

Two major analytical protocols were applied according to the main goals of this study. The first set of analyses addressed the phylogenetic position of *Anindobothrium* within major lineages of cestodes closely related. The second analysis used molecular data as a tool for species discovery and delineation within *Anindobothrium*.

#### Phylogenetic position of *Anindobothrium*

Nucleotide sequences of 18S rDNA and 28S rDNA (D1–D3 regions) were first submitted to phylogenetic analysis by direct optimization (**DO**; [35]) using POY (version 5.1.1; [36]) under parsimony as optimality criteria. Initial tree searches included 10 iterations of two independent searches for 1 h 30 min using the command search [*i.e.*, search(max_time:0:01:30]) assuming equal weights for all character transformations. This search was conducted in a 10 X 2.83 GHz Intel^®^ Core^™^2 Quad Processor Q9550 computer cluster. A DO sensitivity search [37] was performed using nine alignment parameter sets in which gap extension costs varied from one to eight and transformation costs (transversions and transitions) from one to four with an opening gap cost twice that of gap extension cost rendering the following alignment cost ratios for opening gaps, extension gaps, transversions, and transitions, respectively: 0:1:1:1, 2:1:1:1, 2:1:1:2, 2:1:2:1, 2:2:1:1, 2:2:1:2, 2:2:2:1, 2:4:1:1, 2:4:1:2, and 2:4:2:1. For each parameter set, tree space was explored by two independent searches for 2 h [*i.e.*, search(max_time:0:02:00]) in the same computer cluster environment as the previous analysis but using five nodes. After compiling candidate trees by DO, we submitted unique topologies to tree refinement by tree-fusing algorithm [38] and re-diagnosis by iterative pass alignment (**DO/IP+Fuse**; [39]). Our final analytical step under this optimality criterion was to verify the results obtained with DO/IP+Fuse by performing a phylogenetic analysis of the implied alignment (sensu Wheeler [40]) generated by the previous step in TNT [41] using its New Technology searches [41, 42] with the following parameters: rep 100, ratchet 50, fuse 20, hold 10. We evaluated nodal support by using Goodman-Bremer values (GBS, [43–45]; see [46]). To obtain this metric, we considered the shortest tree found by TNT based on the implied alignment above and executed a modified version of the script BREMER.RUN distributed with TNT. This script considered 1,000 replicates with 10 repetitions of ratchet and drift [41, 42] in constrained searches and the remaining default parameters. Finally, putative transformations for selected branches were compiled using the consensus tree obtained by TNT with YBYRÁ [47].

We also analyzed the previous datasets using maximum likelihood (**ML**) to identify nodes sensitive to a different optimality criterion. We started by submitting the implied alignment generated by DO/IP+Fuse to model selection in jModeltest (version 2.1.6; [48, 49]) considering 88 candidate models ranked by AICc scores. Following, tree searches were performed using the parallel implementation of GARLI (version 2.0; [50]) applying 1,000 independent search replicates and remaining default parameters of GARLI configuration file. This search was conducted by implementing 20 searches replicates in 50 X 2.83 GHz Intel^®^ Core^™^2 Quad Processor Q9550 computer cluster. Log-likelihood difference support (LLD; sensu Lee *et al*. [51]; see [52, 53]) was calculated for selected nodes using constrained negative searches in GARLI under the same configuration settings as the initial tree search.

#### Species discovery within *Anindobothrium*

Our first approach was to perform a simultaneous phylogenetic analyses of nuclear regions 18S rDNA, 28S rDNA (D1–D3), Cal, and ITS-1, and the mitochondrial region of COI for all representatives of *Anindobothrium* from three major bodies of water: eastern Pacific Ocean, Caribbean Sea, and Neotropical freshwater systems. We submitted the nucleotide sequences to phylogenetic inference by direct optimization (DO, [35]) using POY (version 5.1.1; [36]) under parsimony as the optimality criterion. Tree search was performed by three independent searches for 30 min using the command search (*i.e.*, search(max_time:0:00:30)) assuming equal weights for character transformations in the same computer cluster environment mentioned above using 10 nodes. All trees compiled by DO were re-diagnosed by iterative pass algorithm (IP, [39]) and the implied alignment (sensu Wheeler, [40]) was submitted to TNT (Golloboff *et al*. [41]) to verify the results using xmu algorithm with 1,000 replicates and holding at the most 10 trees per replicate. A similar analysis was conducted partitioning the dataset into nuclear and mitochondrial regions. The first partition was analyzed in POY as described above and COI was only analyzed in TNT with the same settings as before. Selected clades were diagnosed using YBYRÁ [47] within each partition.

We also performed a phylogenetic analysis using ML as optimality criterion based on the implied alignments resulted from previous analyses. Model selection for the concatenated dataset and for each nuclear region was performed in jModeltest considering 88 candidate models ranked by AICc scores. Under this optimality criterion, phylogenetic analyses were performed for each gene region separately, as well as for two concatenated datasets: one considering only nuclear genes and the other including all regions. We analyzed each concatenated dataset using two different partition models. One considered a single substitution model for all regions and the other considered individual substitution models. Independently of dataset or partition model, tree searches were performed using parallel implementation of GARLI applying a total of 1,000 independent search replicates in 10 X 2.83 GHz Intel^®^ Core^™^2 Quad Processor Q9550 computer cluster. The best partition model was selected based on AICc information criterion.

Congruence between phylogenetic patterns and morphological data was observed by compiling morphometric and meristic data for 137 marine specimens of *Anindobothrium*. The dataset included the newly collected material as well as the type series of *A. anacolum*. A total of 29 measurements were selected, most of which are traditionally used in the taxonomy of the group and missing entries were filled with the mean within clade recovered by phylogenetic inference. All statistical analyses were performed in R as follows. The first step was to identify and exclude all highly correlated measurements (*i.e.*, r > 0.70). Then, a Principal Component Analysis (PCA) was performed to identify whether or not there were any morphological patterns in our data congruent with phylogenetic patterns. Putative groups suggested by phylogenetic analyses and PCA were tested by Linear Discriminant Analysis (LDA; [54, 55]). The error rate of the discriminant function was evaluated by 1,000 iterations of 10-fold cross validation procedure. This dataset and R scripts are available in the repository Dryad under doi:10.5061/dryad.gr0sb.

## Results

### Phylogenetic analyses

#### Phylogenetic position of *Anindobothrium*

To address the phylogenetic position of *Anindobothrium* within the selected lineages of cestodes, 18S and 28S nucleotide data were generated for 27 terminals (Table 1). These terminals included two haplotypes of *Caulobothrium* sp. found in *Potamotrygon* sp. from the Delta of Orinoco, two members of *Rhinebothroides* sp. collected from *Potamotrygon wallacei* de Carvalho, Rosa & de Araujo from the Rio Negro, three specimens of *A. lisae* found in freshwater potamotrygonids from the Rio Negro and Orinoco river basins, and 20 other haplotypes of *Anindobothrium*, most of which collected from species of *Styracura*. In addition to these haplotypes, the dataset included one individual of *Anthocephalum hobergi* (Zamparo, Brooks & Barriga, 1999) Marques & Caira, 2016 from *Urobatis tumbesensis* (Chirichigno & Mc Eachran) off the coast of Ecuador, which was used as out-group taxa. To this dataset, we added 67 selected terminals from Healy *et al*. [2], Caira *et al*. [7], Ruhnke *et al*. [8], and Marques and Caira [10] (Table 2). These additional sequence data included 16 out-group terminals representing members of the Litobothriidea (three), Cathetocephalidea (two), Lecanicephalidea (four), Onchoproteocephalidea (one), Phyllobothriidea (one), and “Tetraphyllidea” (five); and 51 rhinebothriideans, which included members of all families according to Ruhnke *et al*. [8] and Marques and Caira [10]. The complete dataset considered 93 terminals. Sequences of 18S ranged from 1,350 to 1,412 unaligned base pairs (bp)—MAFFT alignment (**MAFFTaln**) resulted in sequences of 1,464 bp—, and sequences of 28S ranged from 803 to 873 unaligned base pairs—MAFFTaln of 1,025 bp.

**Table 1:** Nucleotide sequences generated in the present study and NCBI/Genbank accession numbers.

Locality	species	host	18S	28S	Calm	ITS-1	COI
Caribbean/Belize [10]	*Anindobothrium inexpectatum* sp. n. [MZUSP 7767]	*Styracura schmardae* (BE-002)	MF920380	MF920353	MF947556	MF920330	MF947579
	*Anindobothrium inexpectatum* sp. n. [MZUSP 7768]	*Styracura schmardae* (BE-002)	MF920381	MF920354	MF947557	MF920331	MF947580
	*Anindobothrium inexpectatum* sp. n. [MZUSP 7769]	*Styracura schmardae* (BE-002)	MF920382	MF920355	MF947558	MF920332	MF947581
	*Anindobothrium inexpectatum* sp. n. [MZUSP 7770]	*Styracura schmardae* (BE-003)	MF920383	MF920356	MF947559	MF920333	MF947582
	*Anindobothrium inexpectatum* sp. n. [MZUSP 7771]	*Styracura schmardae* (BE-005)	MF920384	MF920357	MF947560	MF920334	MF947583
	*Anindobothrium inexpectatum* sp. n. [MZUSP 7772]	*Styracura schmardae* (BE-005)	MF920385	MF920358	MF947561	MF920335	MF947584
	*Anindobothrium inexpectatum* sp. n. [MZUSP 7773]	*Styracura schmardae* (BE-011)	MF920386	MF920359	MF947562	MF920336	MF947585
	*Anindobothrium inexpectatum* sp. n. [MZUSP 7774]	*Styracura schmardae* (BE-009)	MF920387	MF920360	MF947563	MF920337	MF947586
	*Anindobothrium inexpectatum* sp. n. [MZUSP 7775]	*Styracura schmardae* (BE-003)	MF920378	MF920351	MF947554	MF920328	MF947577
	*Anindobothrium inexpectatum* sp. n. [MZUSP 7776]	*Styracura schmardae* (BE-005)	MF920379	MF920352	MF947555	MF920329	MF947578
Caribbean/Panama [1]	*Anindobothrium inexpectatum* sp. n. [MZUSP 7777]	*Styracura schmardae* (PN15-54)	MF920377	MF920350	MF947553	MF920327	MF947576
Caribbean/Trinidad & Tobago [4]	*Anindobothrium anacolum* [MZUSP 7778]	*Styracura schmardae* (TT14-06)	MF920372	MF920345	MF947548	MF920322	MF947571
	*Anindobothrium anacolum* [MZUSP 7779]	*Styracura schmardae* (TT14-06)	MF920373	MF920346	MF947549	MF920323	MF947572
	*Anindobothrium anacolum* [MZUSP 7780]	*Styracura schmardae* (TT14-06)	MF920374	MF920347	MF947550	MF920324	MF947573
	*Anindobothrium anacolum* [MZUSP 7789]	*Styracura schmardae* (TT14-06)	MF920371	MF920344	MF947547	MF920321	MF947570
Eastern Pacific/Panama [4]	*Anindobothrium carrioni* sp. n. [MZUSP 7785]	*Styracura pacifica* (PN15-14)	MF920369	MF920342	MF947545	MF920319	MF947568
	*Anindobothrium carrioni* sp. n. [MZUSP 7786]	*Styracura pacifica* (PN15-14)	MF920370	MF920343	MF947546	MF920320	MF947569
	*Anindobothrium carrioni* sp. n. [MZUSP 7787]	*Styracura pacifica* (PN15-14)	MF920375	MF920348	MF947551	MF920325	MF947574
	*Anindobothrium carrioni* sp. n. [MZUSP 7788]	*Styracura pacifica* (PN15-25)	MF920376	MF920349	MF947552	MF920326	MF947575
Eastern Pacific/Ecuador [1]	*Anthocephalum hobergi* [MZUSP 7755]	*Urobatis tumbesensis* (EC-56)	KU295562	KU295566	MF947592	MF939899	MF947591
Neotropical Freshwater/Delta of Orinoco [2]	*Caulobothrium* sp. [MZUSP 7790]	*Potamotrygon* sp. (VZ13-48)	MF920394	MF920367	–	–	–
	*Caulobothrium* sp. [MZUSP 7791]	*Potamotrygon* sp. (VZ13-48)	MF920395	MF920368	–	–	–
Neotropical Freshwater/Maracaibo [1]	*Anindobothrium anacolum* [MZUSP 7781]	*Potamotrygon yepezi* (VZ11-001)	MF920388	MF920361	MF947564	MF920338	MF947587
Neotropical Freshwater/Rio Negro [3]	*Anindobothrium lisae* [MZUSP 7782]	*Potamotrygon orbignyi* (RN11-028)	MF920389	MF920362	MF947565	MF920339	MF947588
	*Anindobothrium lisae* [MZUSP 7783]	*Potamotrygon schroederi* (RN11-058)	MF920390	MF920363	MF947566	MF920340	MF947589
	*Anindobothrium lisae* [MZUSP 7784]	*Potamotrygon orbignyi* (VZ11-029)	MF920391	MF920364	MF947567	MF920341	MF947590
Neotropical Freshwater/Rio Negro [2]	*Rhinebothroides* sp. [MZUSP 7792]	*Potamotrygon wallacei* (RN11-009)	MF920392	MF920365	–	–	–
	*Rhinebothroides* sp. [MZUSP 7793]	*Potamotrygon wallacei* (RN11-030)	MF920393	MF920366	–	–	–

**Table 2:** Nucleotide sequences for members of the Rhinebothriidea and related cestodes taxa included in the analysis.

Suprageneric taxon	species	host	NCBI 18S	NCBI 28S	source
Litobothriidea/Litobothriidae [3]*	*Litobothrium amplifica* [TE-26/LRP 8279]	*Alopias pelagicus* (BJ-713)	KF685843	KF685906	Caira *et al*. [7]
	*Litobothrium janovyi* [BMNH 2000.3.7.3-5]	*Alopias superciliosus* (BJ-716)	AF124468	AF286930	Caira *et al*. [7]
	*Litobothrium nickoli* [TE-113/LRP 8321]	*Alopias pelagicus* (BJ-713)	KF685844	KF685907	Caira *et al*. [7]
Cathetocephalidea/Cathetocephalidae [2]*	*Cathetocephalus thatcheri* [TE-28/LRP 8281]	*Carcharhinus leucas*	KF685838	KF685884	Caira *et al*. [7]
Cathetocephalidea/Disculicipitidae	*Disculiceps* sp. 1 [TE-130/LRP 8328	*Carcharhinus limbatus* (MS05-24)	KF685839	KF685761	Caira *et al*. [7]
Lecanicephalidea/Cephalobothriidae [4]*	*Adelobothrium aetobatidis* [TE-16B/LRP 8272]	*Aetobatus ocellatus* (AU-57)	EF095249	EF095257	Caira *et al*. [7]
Lecanicephalidea/Paraberrapecidae	*Paraberrapex manifestus* [TE-142]	*Squatina californica* (BJ-298)	KF685781	KF685868	Caira *et al*. [7]
Lecanicephalidea/Polypocephalidae	*Anteropora patulobothridium* [TE-90/LRP 8307]	*Taeniura lymma 1* (BO-86)	KF685788	KF685863	Caira *et al*. [7]
	*Polypocephalus helmuti* [TE-17A/LRP 8273]	*Rhinoptera neglecta* (AU-85)	KF685786	KF685869	Caira *et al*. [7]
Onchoproteocephalidea [1]*	*Acanthobothrium santarosaliense* [TE-136/LRP 8300]	*Heterodontus mexicanus* (BJ-300)	KF685834	KF685751	Caira *et al*. [7]
Phyllobothriidea/Phyllobothriidae [1]*	*Anthobothrium* sp. 1 [TE-119/LRP 8325]	*Carcharhinus tilstoni* (NT-55)	KF685806	KF685752	Caira *et al*. [7]
“Tetraphyllidea” [5]*	*Caulobothrium* sp. 5 [CH-25/LRP 3914]	*Pastinachus ater* (NT-105)	FJ177065	FJ177105	Caira *et al*. [7]
	*Caulobothrium opisthorchis* [CH-21/LRP 3910]	*Myliobatis californica* (BJ-626)	FJ177066	FJ177106	Caira *et al*. [7]
	*Duplicibothrium minutum* [TE-135/LRP 8332]	*Rhinoptera* cf.*steindachneri* (MS05-49)	KF685809	KF685885	Caira *et al*. [7]
	New genus 7 sp. 1 [TE-166/LRP 8344]	*Parascyllium collare* (KJG-17)	KF685851	KF685749	Caira *et al*. [7]
	*Pentaloculum* sp. 1 [TE-171/LRP 8347]	*Typhlonarke tarakea* (CR-136)	KF685852	KF685877	Caira *et al*. [7]
Rhinebothriidea/Anthocephalidae [24]	*Anthocephalum alicae* [PEET-319/LRP 8508]	*Dasyatis americana* (TM-19)	KM658190	KM658205	Ruhnke *et al*. [8]
	*Anthocephalum cairae* [PEET-320/LRP 8509]	*Dasyatis americana* (TM-19)	KM658187	KM658202	Ruhnke *et al*. [8]
	*Anthocephalum currani* [PEET-403/LRP 8510]	*Dasyatis dipterura* (BJ-119)	KM658188	KM658203	Ruhnke *et al*. [8]
	*Anthocephalum decristanisorum* [PEET-427/LRP 8511]	*Himantura uarnacoides* (BO-91)	KM658179	KM658194	Ruhnke *et al*. [8]
	*Anthocephalum healyae* [PEET-258/LRP 8512]	*Neotrygon australiae* (CM03-42)	KM658185	KM658200	Ruhnke *et al*. [8]
	*Anthocephalum hobergi* [EC-14.1/MZUSP 7754]	*Urobatis tumbesensis* (EC-14)	KU295561	KU295565	Marques & Caira [10]
	*Anthocephalum hobergi* [EC-56.1/MZUSP 7756]	*Urobatis tumbesensis* (EC-56)	KU295562	KU295566	Marques & Caira [10]
	*Anthocephalum hobergi* [EC-56.2/MZUSP 7757]	*Urobatis tumbesensis* (EC-56)	KU295563	KU295567	Marques & Caira [10]
	*Anthocephalum hobergi* [EC-56.3/MZUSP 7755]	*Urobatis tumbesensis* (EC-56)	KU295564	KU295568	Marques & Caira [10]
	*Anthocephalum jensenae* [PEET-300/LRP 8513]	*Himantura jenkensii* (NT-118)	KM658178	KM658193	Ruhnke *et al*. [8]
	*Anthocephalum mattisi* [TE-141/LRP 4219]	*Dasyatis* sp. (SE-222)	FJ177059	FJ177099	Caira *et al*. [7]
	*Anthocephalum meadowsi* [PEET-297/LRP 8514]	*Himantura leoparda* (NT-32)	KM658180	KM658195	Ruhnke *et al*. [8]
	*Anthocephalum michaeli* [PEET-267/LRP 8515]	*Dasyatis longa* (BJ-423)	KM658189	KM658204	Ruhnke *et al*. [8]
	*Anthocephalum* sp. 1 [PEET-256/LRP 8505]	*Himantura leoparda* (NT-32)	KM658191	KM658206	Ruhnke *et al*. [8]
	*Anthocephalum* sp. 2 [PEET-413/LRP 8506]	*Taeniurops grabata* (SE-121)	KM658183	KM658198	Ruhnke *et al*. [8]
	*Anthocephalum* sp. 3 [PEET-409/LRP 8507]	*Urogymnus asperrimus* 1 (CM03-53)	KM658177	KM658192	Ruhnke *et al*. [8]
	*Anthocephalum odonellae* [PEET-299/LRP 8516]	*Neotrygon kuhlii1* (BO-336)	KM658186	KM658201	Ruhnke *et al*. [8]
	*Anthocephalum papefayi* [PEET-259/LRP 8517]	*Dasyatis margaritella* (SE-225)	KM658184	KM658199	Ruhnke *et al*. [8]
	*Anthocephalum philruschi* [PEET-425/LRP 8518]	*Himantura uarnak* 2 (CM03-24)	KM658181	KM658196	Ruhnke *et al*. [8]
	*Barbeaucestus ralickiae* [CH-35/LRP 3922]	*Taeniura lymma* 1 (BO-86)	FJ177068	FJ177108	Caira *et al*. [7]
	*Barbeaucestus shipleyi* [CH-3/LRP 3894]	*Neotrygon orientale* (BO-336)	FJ177069	FJ177109	Caira *et al*. [7]
	*Divaricobothrium tribelum* [CH-11/LRP 3902]	*Himantura* *gerrardi* (BO-466)	FJ177067	FJ177107	Caira *et al*. [7]
	*Sanguilevator yearsleyi* [TE-114/LRP 4218]	*Lamiopsis tephrodes* (BO-488)	FJ177057	KF685762	Caira *et al*. [7]
	*Sungaicestus kinabatanganensis* [CH-9/LRP 3900]	*Urogymnus polylepis* (BO-108)	FJ177078	FJ177118	Caira *et al*. [7]
Rhinebothriidea/Echeneibothriidae [3]	*Echeneibothrium* sp. 1 [TE-94/LRP 4217]	New genus B *velezi* (BJ-243)	FJ177058	FJ177098	Caira *et al*. [7]
	*Echeneibothrium* sp. 2 [TE-95/LRP 8312]	*Raja* cf.*miraletus* (SE-188)	KF685842	KF685876	Caira *et al*. [7]
	*Pseudanthobothrium* sp. 1 [TE-117/LRP 8324]	*Leucoraja erinacea* (HM-7)	KF685841	KF685750	Caira *et al*. [7]
Rhinebothriidea/Escherbothriidae [9]	*Escherbothrium sp.* [PEET-424/LRP 8519]	*Urotrygon* sp. 1 (CRP-50)	KM658182	KM658197	Ruhnke *et al*. [8]
	Rhinebothriinae New genus 3 sp. 1 [CH-7/LRP 3898]	*Fontitrygon margaritella* (SE-125)	FJ177071	FJ177111	Healy *et al*. [2]
	Rhinebothriinae New genus 3 sp. 2 [CH-8/LRP 3899]	*Fontitrygon margaritella* (SE-125)	FJ177072	FJ177112	Healy *et al*. [2]
	Rhinebothriinae New genus 3 sp. 4 [CH-15/LRP 3906]	*Himantura astra* (NT-26)	FJ177074	FJ177114	Healy *et al*. [2]
	*Stillabothrium amuletum* [CH-30/LRP 3917]	*Glaucostegus typus* (AU-56)	FJ177077	FJ177117	Caira *et al*. [7]
	*Stillabothrium cadenati* [CH-37/LRP 3924]	*Zanobatus schoenleinii* (SE-201)	FJ177070	FJ177110	Caira *et al*. [7]
	*Stillabothrium davicynthiae* [CH-45/LRP 3926]	*Himantura heterura* (BO-237)	FJ177076	FJ177116	Caira *et al*. [7]
	*Stillabothrium* sp. 3 [CH-14/LRP 3905]	*Himantura astra* (NT-26)	FJ177073	FJ177113	Healy *et al*. [2]
	*Stillabothrium* sp. 5 [CH-20/LRP 3909]	*Himantura leoparda* (NT-117)	FJ177075	FJ177115	Healy *et al*. [2]
Rhinebothriidea/Rhinebothriidae [14]	*Rhabdotobothrium anterophallum* [BMNH 2001.1.31.3-4]	*Mobula hypostoma* (M-99-2442)	AF287000	AF286961	Caira *et al*. [7]
	*Rhinebothrium cf. maccallumi* [LRP 2108]	*Dasyatis americana*	AF124476	AF286962	Caira *et al*. [7]
	*Rhinebothrium megacanthophallus* [CH-10/LRP 3901]	*Urogymnus polylepis* (BO-108)	FJ177080	FJ177120	Caira *et al*. [7]
	*Rhinebothrium* sp. 1 [CH-12/LRP 3903]	*Himantura* cf.*pastinacoides* (BO-76)	FJ177081	FJ177121	Caira *et al*. [7]
	*Rhinebothrium* sp. 4 [CH-1/LRP 3892]	*Dasyatis akajei* (JN-1)	FJ177086	FJ177126	Healy *et al*. [2]
	*Rhinebothrium* sp. 5 [CH-2/LRP 3893]	*Dasyatis dipterura* (BJ-51)	FJ177087	FJ177127	Healy *et al*. [2]
	*Rhinebothrium* sp. 7 [CH-6/LRP 3897]	*Fontitrygon margarita* (SE-123)	FJ177089	FJ177129	Caira *et al*. [7]
	*Rhinebothrium* sp. 8 [CH-55/LRP 3930]	*Paratrygon aiereba* (PU-10)	FJ177090	FJ177130	Caira *et al*. [7]
	*Rhinebothrium* sp. 9 [CH-34/LRP 3921]	*Taenirua lymma* 1 (BO-86)	FJ177091	FJ177131	Caira *et al*. [7]
	*Rhinebothroides* cf.*freitasi* [CH-54/LRP 3929]	*Potamotrygon castexi* (PU-25b)	FJ177092	FJ177132	Caira *et al*. [7]
	*Rhodobothrium paucitesticulare* [TE-61/LRP 4216]	*Rhinoptera bonasus* (BNC-22)	FJ177060	FJ177100	Caira *et al*. [7]
	*Scalithrium* sp. [CH-4/LRP 3895]	*Dasyatis longa* (BJ-423)	FJ177093	FJ177133	Caira *et al*. [7]
	*Scalithrium* sp. 1 [TE-140/LRP 8333]	*Dasyatis longa* (BJ-423)	KF685840	KF685878	Caira *et al*. [7]
	*Spongiobothrium* sp. [CH-32/LRP 3919]	*Rhynchobatus palpebratus* (NT-66)	FJ177094	FJ177134	Caira *et al*. [7]
Rhinebothriidea [1]	New genus 11 sp. 1 [CH-26/LRP 4220]	*Pristis clavata* (AU-36)	FJ177079	FJ177119	Healy *et al*. [2]

* Out-group taxa.

**Fig 1:**
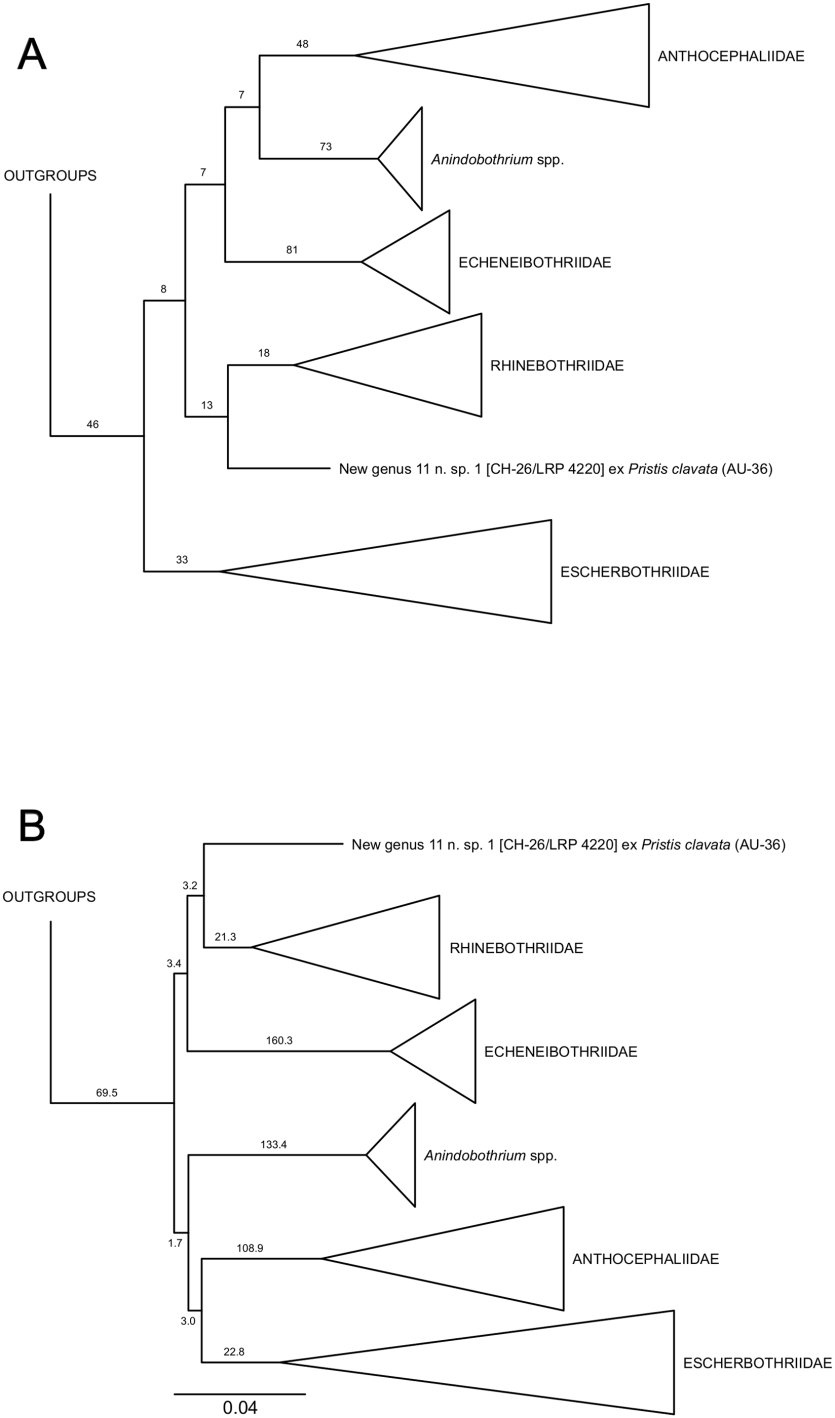
Summary of sister-group relationships within the Rhinebothriidea based on the simultaneous analysis of 18S and 28S rDNA regions. A. Topology inferred under parsimony as the optimality criterion with 6,346 steps in length and Goodman-Bremer support values for selected clades. B. Topology inferred under Maximum Likelihood as the optimality criteria (-lnL 28523.9385) and Likelihood Length Difference support values for selected clades. “New genus 11” refers to Rhinebothriinae n. sp.1 of Healy *et al*. [2].

Tree search using direct optimization under equal weights for all transformations was based on 1,279 builds followed by TBR (Tree-bisectioning and redraft, [56]), 22,032 cycles of tree fusing [38], and 622 iterations of ratchet [42]. Sensitivity search included 186 builds followed by TRB, 2,437 cycles of tree fusing, and 88 iterations of ratchet. Combined, the tree search under direct optimization found 72 unique topologies ranging from 6,407 to 6,663 steps in length. The re-diagnosis of these 72 unique topologies under iterative pass algorithm followed by tree fusing rendered a single topology with 6,350 transformations. The implied alignment submitted to TNT, with 1,698 bp for 18S and 1,299 bp for 28S, resulted in two topologies with 6,346 steps in length. These two trees differed on internal arrangements of apical terminals with near-zero branch lengths. Fig 1A displays the summary results of this analysis, including GBS support for selected nodes. A topology with all terminals is provided in supplementary results (S1 Fig).

Implied alignment resulted from the analyses above was submitted to ML phylogenetic inference assuming GTR+Γ+I as the substitution model. This analysis resulted in a topology with -lnL 28523.9385, which summary is presented in Fig 1B along with values of Likelihood Length Difference as a measure of support for selected clades. The detailed sister-group relationships hypothesized by ML analysis is presented in supplementary results (S2 Fig).

Both optimality criteria supported the monophyly of families but suggested different sets of sister-group relationships (Fig 1A and 1B). These differences involve clades with relatively lower support. According to the parsimony analysis, *Anindobothrium* is sister to the Anthocephaliidae, whereas ML topology suggested it is sister to the clade Anthocephaliidae+Escherbothriidae.

**Fig 2:**
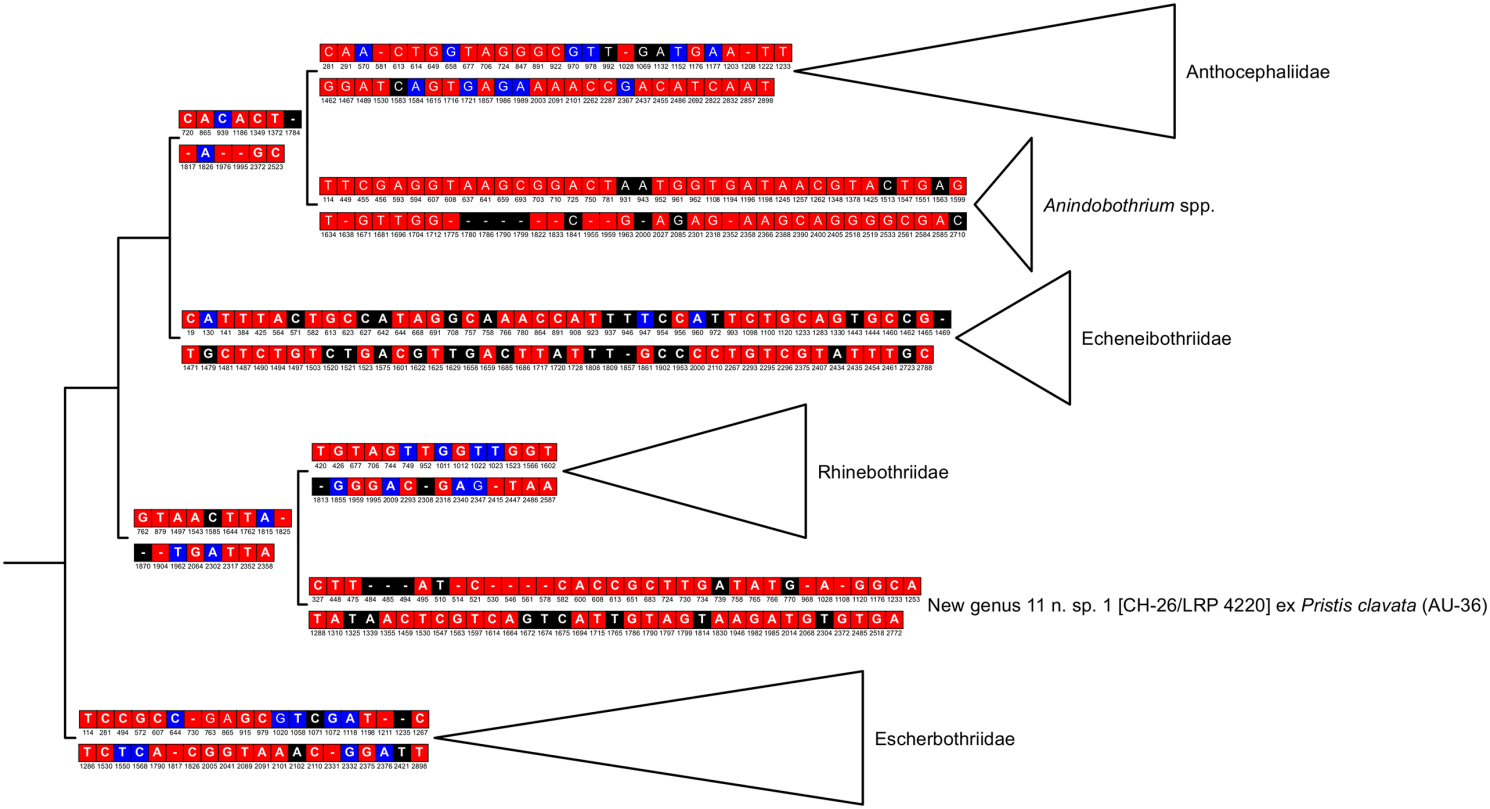
Summary of sister-group relationships based on the simultaneous analysis of 18S and 28S rDNA regions using parsimony as the optimality criteria for rhinebothriideans. Division between sets of synapomorphies indicates transformations from 18S and 28S rDNA, respectively. For each base pair, black cells represent unambiguous unique synapomorphies, red cells represent unique homoplastic synapomorphies, and blue cells represent non-unique homoplastic synapomorphies (see 45). Numbers below each cell represent alignment position for respective gene regions.

Although the internal branches supporting sister-group relationships among families and genera, such as *Anindobothrium* and the “New genus 11” (Rhinebothriinae n. sp.1 of Healy *et al*. [2]), have relatively lower support, all families have a relative high support (Fig 1A and 1B). Also, branches leading to clades for recognized families, representatives of *Anindobothrium* nested, and for the “New genus 11” possess sets of molecular synapomorphies that could be used as putative diagnostic nucleotides for each of them (Fig 2). For instance, *Anindobothrium* is supported by 30 transformations from the 18S region, three of which are observable (non-gap), unique, unambiguous synapomorphies, and 44 from 28S region, four of which are observable (non-gap), unique, unambiguous synapomorphies. The amount of transformations inferred for *Anindobothrium* (74) is smaller than what was recovered for the Echeneibothriidae de Beauchamp, 1905 (86) but larger when compared to all other families (Fig 2). Implied alignment data files and consensus tree used to provide diagnosis for each clade of the Rhinebothriidea are available in the repository Dryad under doi:10.5061/dryad.gr0sb.

#### Species discovery within *Anindobothrium*

**Molecular data**: Our phylogenetic analyses utilized 24 terminals, 23 haplotypes of *Anindobothrium* and one specimen of *Anthocephalum hobergi* used to root the tree (Table 1). For this dataset, unaligned sequences of 18S ranged from 1,365 to 1,384 bp (MAFFTaln resulted in sequences of 1,384 bp), 28S (D1–D3) ranged from 1,112 to 1,133 bp (MAFFTaln = 1,143 bp), Calmodulin ranged from 386 to 388 bp (MAFFTaln = 388 bp), COI ranged from 477 to 549 bp (MAFFTaln = 549 bp), and ITS ranged from 678 to 713 bp (MAFFTaln = 806 bp).

The biogeographical representation of this dataset included three individuals identified as *A. lisae* (one) ex *Potamotrygon schroederi* from the Rio Negro and (two) ex *P. orbignyi* from the Rio Negro and Mid-Orinoco river basins, three terminals identified as *A. anacolum* from Trinidad & Tobago parasitizing *Styracura schmardae* and one from Lake Maracaibo infecting *P. yepezi*. We also included 15 members of *Anindobothrium*, among which 10 individuals were from Belize and one from the Caribbean coast of Panama recovered from *S. schmardae*, and four worms parasitizing *S. pacifica* from the eastern Pacific coast of Panama (Table 1).

**Fig 3:**
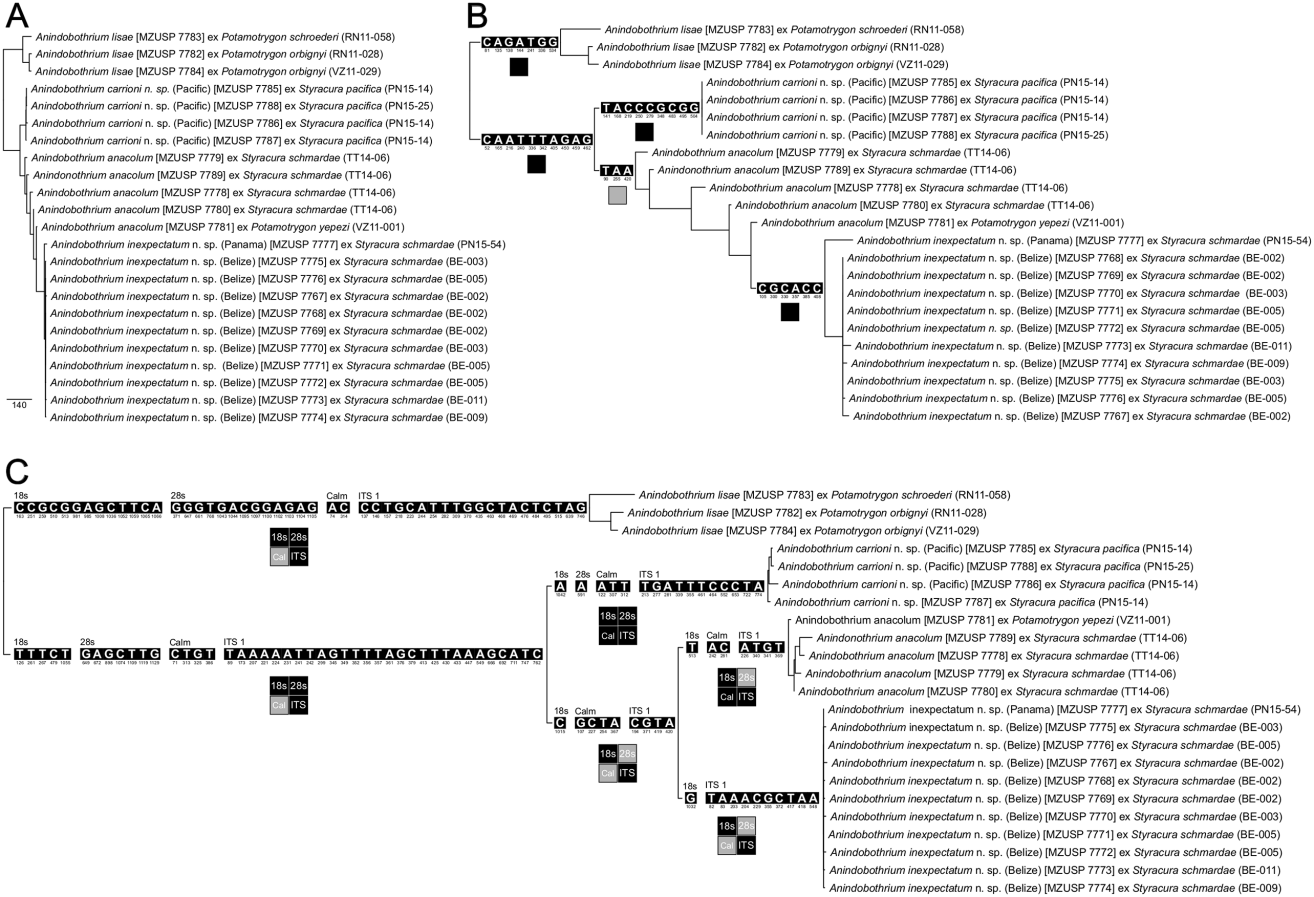
Phylogenetic relationships among haplotypes of *Anindobothrium* based on parsimony analysis of nucleotide sequences. A. Phylogeny based on the simultaneous analysis of 18S, 28S, COI, and ITS-1 for members of *Anindobothrium* by direct optimization of nucleotide data (tree length = 1,245). B. Phylogeny based on the parsimony analysis of COI (tree length = 371). C. Phylogeny based on the simultaneous analysis of nuclear regions (18S, 28S and ITS-1) by direct optimization of nucleotide data (tree length = 863). Base pairs on branches represent unambiguous unique synapomorphies for respective clades. Squares under branches represent presence (black) or absence (gray) of selected clades in the ML analysis.

Tree search using direct optimization under equal weights for all transformations was based on 126 builds followed by TRB, 13,465 cycles of tree fusing, and 79 iterations of ratchet, found 144 trees, 1,249 steps long. The re-diagnosis of all trees using iterative pass, utilizing 1,386 bp for 18S, 1,148 bp for 28S, 393 bp for Calmodulin, 549 bp for COI, and 803 bp for ITS, found all of them to have 1,245 steps. The phylogenetic analysis of the implied alignment resulted in 11 trees equally parsimonious with 1,245 steps. The consensus tree of these 11 topologies (Fig 3A) suggested the monophyly of *A. lisae*, the haplotypes of *Anindobothrium* from eastern Pacific, and a clade comprised by members of the genus from Belize and Panama (Caribbean). However, *A. anacolum* resulted as paraphyletic. The same phylogenetic pattern was observed when the partition for COI was analyzed separately in TNT. This analysis found 12 topologies at a cost of 371 steps, for which the consensus tree is presented in Fig 3B. Our analysis based on direct optimization of nuclear genes included 184 builds, 22,826 cycles with tree fusing, and 156 iterations of ratchet and found 215 trees with 868 steps. The re-diagnosis of these trees by iterative pass found all of them to have 863 steps and the implied alignment analyzed in TNT rendered two topologies with same cost. Contrary to COI, the nuclear genes recognized four monophyletic groups within *Anindobothrium*. Among those not recognized by COI, *A. anacolum* resulted as a monophyletic group (Fig 3C). Implied alignment data files and consensus tree used to provide diagnosis for each clade of *Anindobothrium* are available in the repository Dryad under doi:10.5061/dryad.gr0sb.

The ML analysis rendered similar results. Model selection suggested that the best fitting model for the concatenated dataset was TVM+Γ+I, whereas for 18S, 28S, Cal, COI, and ITS-1 it was TPM3uf+Γ, TIM2+Γ, TIM1+Γ, TPM1uf+I, and TPM1uf+Γ, respectively. For the simultaneous analysis of all data we utilized two partition models. One analysis utilized the model TVM+Γ+I for all concatenated partitions and the other assumed distinct substitution models for each gene regions separately. The AICc favored the partition model in which different substitution models were assigned to each region (Table 3). This analysis recovered the same phylogenetic pattern as obtained by parsimony analysis (Fig 3A). The phylogenetic analysis of COI using ML corresponded to most of the nodes recovered by parsimony analysis (see Fig 3B). For nuclear genes, the partition model also favored different substitution models for each partition (Table 3). The ML analysis of nuclear regions displayed the same topology as obtained for the parsimony analysis (see Fig 3B). However, ML analysis of individual partitions did not recover some nodes.

**Table 3:** Partition model tests for ML phylogenetic analyses including haplotypes of *Anindobothrium*.

Model	# Taxa	# Branches	# EPSM	K	Char.	*lnL*	AIC	AICc
All genes single	24	45	9	79	356	-11012.38731	22182.77463	22227.42680
All genes partition	24	45	32	102	483	-10598.46585	21400.93171	21455.15276
Nuclear single	24	45	7	77	220	-8222.02870	16598.05741	16680.47994
Nuclear partition	24	45	25	95	320	-8065.99067	16321.98134	16401.71348

# Taxa, number of taxons analyzed; # Branches, number of branches; # EPSM, number of free (estimated) parameters in substitution models; K, total number of free parameter, which includes topology, branch lengths, and free parameters in the substitution model(s); # Char, number of characters utilized in ML analyses (unique patterns); *lnL*, negative Log-Likelihood scores; AIC, Akaike Information Criterion score of partition models; AICc, Corrected Akaike Information Criterion score of partition models.

A comparison of KP2 distances among representatives of the marine clades showed that COI was the most divergent region used in this study (Table 4). On average, sequences of COI from haplotypes of *Anindobothrium* collected in the eastern Pacific Ocean differed in 13.8% from representatives of the genus collected in the Caribbean Sea. Between the clades in the Caribbean, *A. anacolum* differed from *A. inexpectatum* sp. n. in 10.5% (see Table 4). Calmodulin and ITS were together the second most divergent regions. In general, for both regions, specimens from the eastern Pacific differed in 2.7% from those collected in the Caribbean and the differences between *A. anacolum* and *A. inexpectatum* sp. n. were 1.0% and 1.3%, respectively. Finally, 18S and 28S regions showed little variation within marine haplotypes of *Anindobothrium* (Table 4).

**Table 4:** Kimura’s two-parameter (K2P) distances among marine lineages of *Anindobothrium*. Distances are presented as average distance and ranges between brackets.

**18S**	
*A. carrioni* sp. n. vs. *A. anacolum*	0.003 (0.002-0.007)
*A. carrioni* sp. n. vs. *A. inexpectatum* sp. n.	0.003 (0.003-0.004)
*A. carrioni* sp. n. vs. *A. anacolum* + *A. inexpectatum* sp. n.	0.003 (0.002-0.007)
*A. anacolum* vs. *A. inexpectatum* sp. n.	0.003 (0.002-0.008)
**28S**	
*A. carrioni* sp. n. vs. *A. anacolum*	0.004 (0.002-0.006)
*A. carrioni* sp. n. vs. *A. inexpectatum* sp. n.	0.003 (0.003-0.005)
*A. carrioni* sp. n. vs. *A. anacolum* + *A. inexpectatum* sp. n.	0.003 (0.002-0.006)
*A. anacolum* vs. *A. inexpectatum* sp. n.	0.002 (0.001-0.003)
**Cal**	
*A. carrioni* sp. n. vs. *A. anacolum*	0.032 (0.026-0.037)
*A. carrioni* sp. n. vs. *A. inexpectatum* sp. n.	0.024 (0.021-0.032)
*A. carrioni* sp. n. vs. *A. anacolum* + *A. inexpectatum* sp. n.	0.027 (0.021-0.037)
*A. anacolum* vs. *A. inexpectatum* sp. n.	0.010 (0.005-0.016)
**COI**	
*A. carrioni* sp. n. vs. *A. anacolum*	0.097 (0.049-0.150)
*A. carrioni* sp. n. vs. *A. inexpectatum* sp. n.	0.157 (0.144-0.162)
*A. carrioni* sp. n. vs. *A. anacolum* + *A. inexpectatum* sp. n.	0.138 (0.049-0.162)
*A. anacolum* vs. *A. inexpectatum* sp. n.	0.105 (0.065-0.136)
**ITS**	
*A. carrioni* sp. n. vs. *A. anacolum*	0.030 (0.025-0.036)
*A. carrioni* sp. n. vs. *A. inexpectatum* sp. n.	0.025 (0.022-0.030)
*A. carrioni* sp. n. vs. *A. anacolum* + *A. inexpectatum* sp. n.	0.027 (0.022-0.036)
*A. anacolum* vs. *A. inexpectatum* sp. n.	0.013 (0.010-0.016)

**Fig 4:**
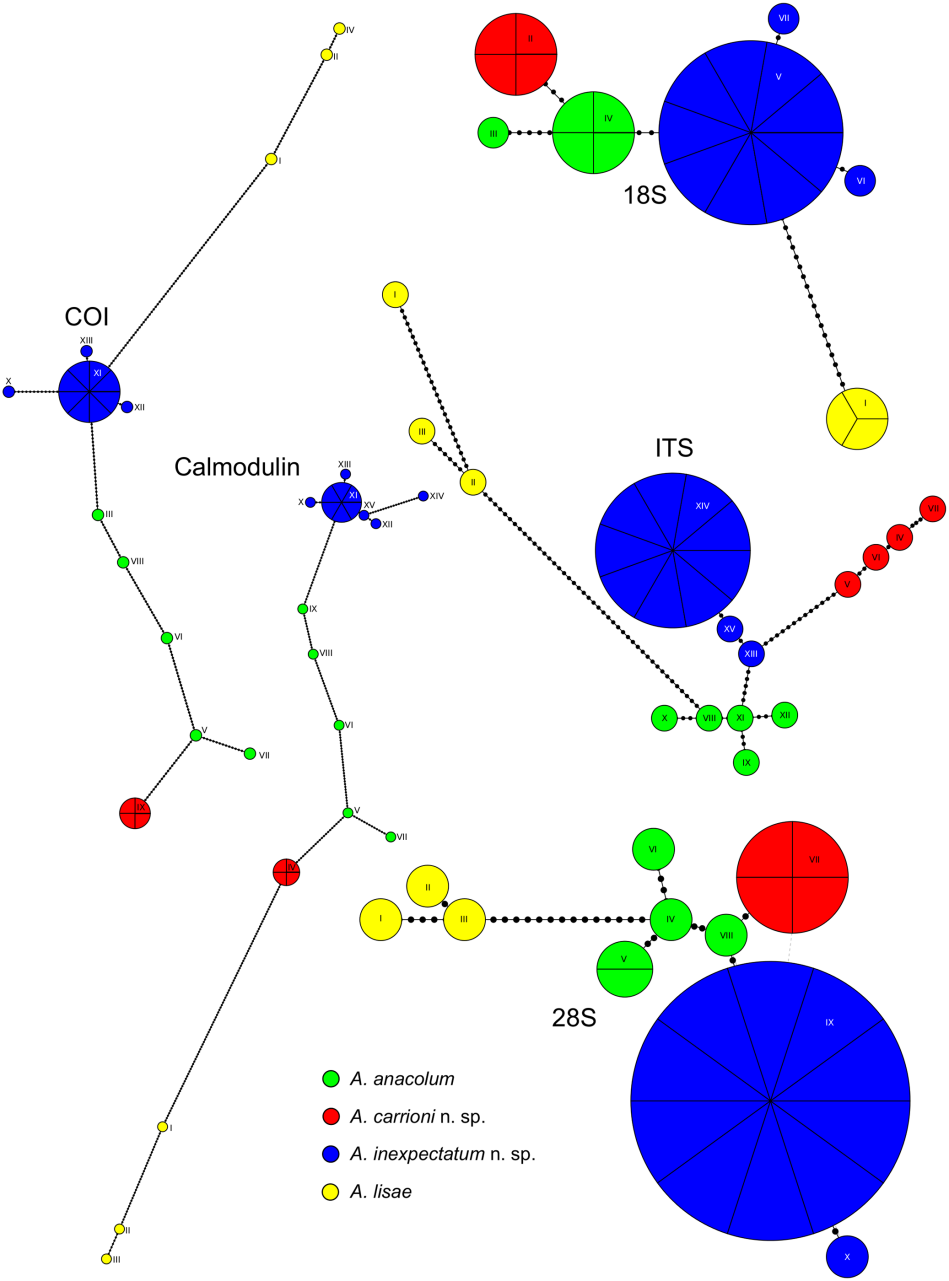
Haplotype networks based on uncorrelated distances of 23 nucleotide sequences from COI mtDNA and nuclear regions 18S, 28S, Calmodulin, and ITS for putative species of *Anindobothrium*. Sizes of the circles are proportional to haplotype frequency—number of divisions indicate numbers of individuals sharing the same haplotype, and the lengths of the connecting lines are proportional to the number of mutational steps (dots on the lines) between haplotypes—dashed lines represent alternative links. Number of haplotypes found: 18S = 7, 28S = 10, Calmodulin = 15, COI = 13, and ITS = 15.

Haplotype networks of the mitochondrial region COI and the nuclear markers 18S, 28S, Calmodulin, and ITS revealed some interesting patterns (Fig 4). The most obvious is the observation that there is no shared haplotypes among the biogeographical areas sampled (*i.e.*, freshwater rivers of South America, eastern Pacific Ocean, and the Caribbean coasts of Central and South America). As expected, the haplotypes of *A. lisae* are the most divergent among *Anindobothrium*. For genes with low substitution rates, such as 18S and 28S, putative new species were segregated from others by few mutational steps. For instance, *A. carrioni* sp. n. and *A. inexpectatum* sp. n. were segregated from *A. anacolum* by 3 and 2 mutational steps, respectively, according to 18S sequences (Fig 4). A similar pattern is observed for 28S in which *A. anacolum* is separated from these two new species by a single mutational step. However, these newly recognized lineages are well segregate from others by faster evolving genes (*i.e.*, Calmodulin, COI, and ITS; see Fig 4).

**Morphological data**: The morphological dataset utilized 29 morphometric variables for 137 specimens. We focused on the marine representatives of *Anindobothrium* considering the following representation: 29 specimens of *A. anacolum* from the type locality—including five individuals from the type series (USNPC 73969, holotype and HMWL 20265a–d, paratypes), 24 individuals attributed to *A. anacolum* from Trinidad & Tobago, 41 worms from the Caribbean representing the clade formed by specimens collected in *Styracura schmardae* from Belize (31) and Panama (10), and 32 specimens of *Anindobothrium* collected from *S. pacifica* from the eastern Pacific coast of Panama. Eight of the initial 29 morphometric variables were found to be highly correlated (r > 0.70, see supplementary S1 Table) and were excluded from further analyses.

**Fig 5:**
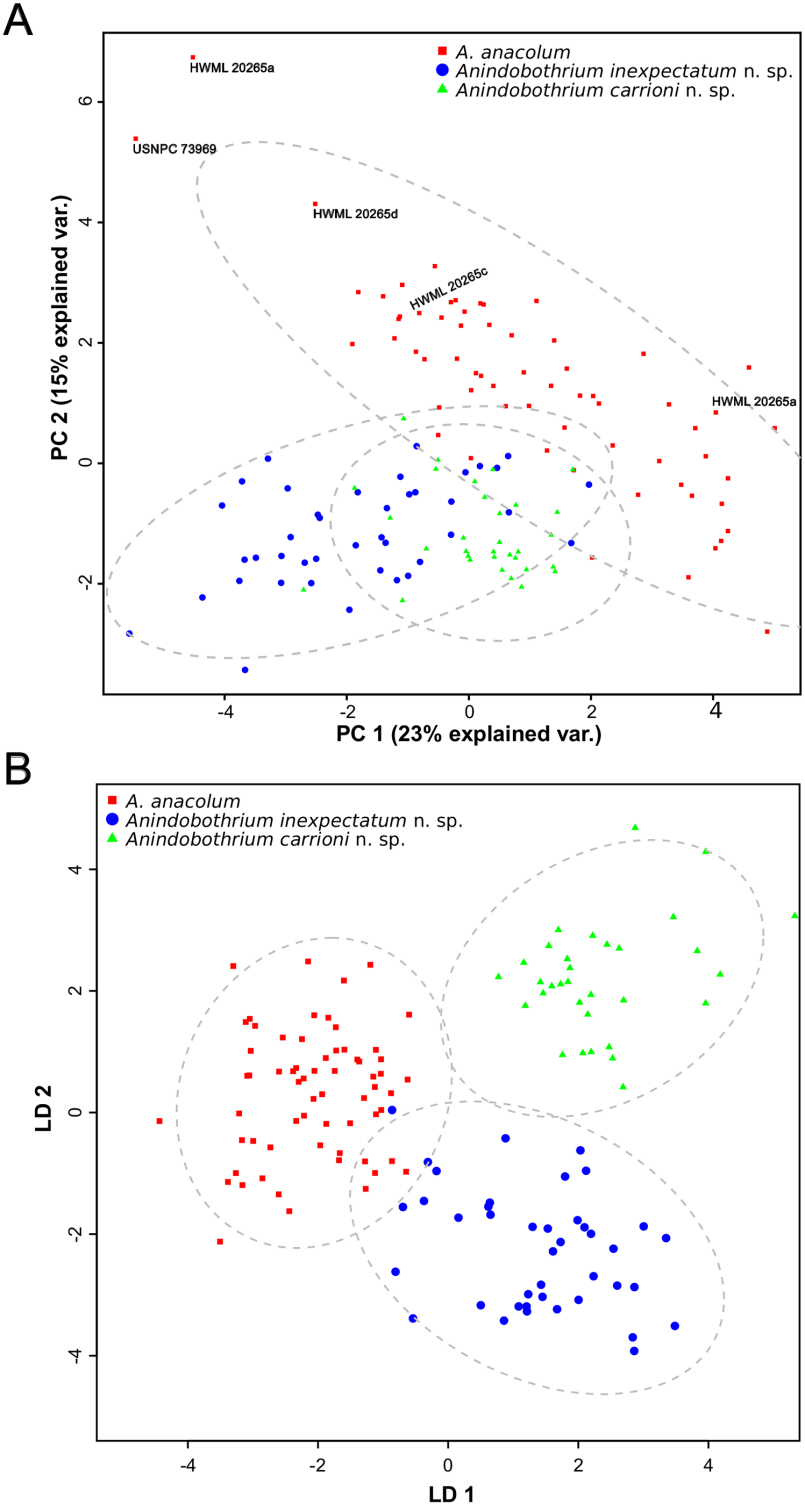
Multivariate statistical analysis of morphological data from marine lineages of *Anindobothrium*. A. Principal Component Analysis (PCA). B. Linear Discriminant Analysis (LDA).

The PCA utilizing 21 morphometric variables suggested that PCA1 explains 23% whereas PCA2 explains 15% of total variance (Fig 5A). The centroid around the means (95% confidence) suggested that individuals assigned to *A. anacolum* clustered together with most of the specimens from its type series and worms collected in the type locality as well as from Trinidad & Tobago. The results of this analysis revealed a great overlap between members of this genus collected in the eastern Pacific Ocean and from the Caribbean coast of Panama/Belize. These two populations overlapped at the edge of the centroid of *A. anacolum*. The loadings of PCA1 indicate that total length, bothridial length and scolex width have greater influence in that component, whereas for PCA2, most of the variance is due to the length of poral testes and cirrus sac dimensions (S2 Table).

The LDA was performed to evaluate whether the recognized marine lineages based on molecular data could be discriminated by morphological data. Our analysis was able to discriminate representatives of marine clades (Fig 5B). The proportion of traces (*i.e.*, the percentage separation achieved by each discriminant function) was 59% for LDA1 and 41% for LDA2. Loadings for each discriminant function suggested that terminal mature proglottid ratio and number of testes are the most important measurements for LDA1, whereas the number of mature proglottids and vitelline follicles length were most important measurements for LDA2 (S3 Table). The cross validation procedure indicated a discriminant function error rate of 3%.

In summary, our results indicated that the phylogenetic position of *Anindobothrium* was sensitive to optimality criteria, especially in nodes that displayed relative lower support. However, we found that the amount of molecular divergence in the branch supporting the monophyly of *Anindobothrium* was as great as, if not greater than, those found in branches of presently recognized families within the Rhinebothriidea. For *Anindobothrium*, we were able to recognize four independent lineages, two of them already described, *A. anacolum* and *A. lisae*, whereas the other two require formal description. The recognition of these lineages was not only based on molecular data but also supported by the LDA analysis. Based on these results the proposed taxonomic actions are as follows.

### Nomenclatural acts

The electronic edition of this article conforms to the requirements of the amended International Code of Zoological Nomenclature, and hence the new names contained herein are available under that Code. This published work and the nomenclatural acts it contains have been registered in ZooBank, the online registration system for the ICZN. The ZooBank LSIDs (Life Science Identifiers) can be resolved and the associated information viewed through any standard web browser by appending the LSID to the prefix “http://zoobank.org/”. The LSID for this publication is: urn:lsid:zoobank.org:pub:0CC9ACE3-9691-4043-8695-B70B6C4A2342. The electronic edition of this work was published in a journal with an ISSN, and has been archived and is available from the following digital repositories: PubMed Central and LOCKSS.

#### Anindobothriidae fam. n.

urn:lsid:zoobank.org:act:1D6F5223-DB13-42D8-A70F-514D56FD133F

**Diagnosis**: Scolex with four stalked bothridia; bothridia with marginal loculi, 2 columns of regular facial loculi present or absent; bothridial apical sucker present; anterior to posterior orientation of bothridia conspicuous; myzorhynchus lacking in adult stage. Genital pore anterior. Postvaginal testes present. Vitelline follicles with partial or total interruption by ovary. Parasites of marine and freshwater stingrays of the family Potamotrygonidae.

**Type and only genus**: *Anindobothrium* Marques, Brooks & Lasso, 2001.

**Remarks**: The phylogenetic analysis of molecular data provided unequivocal evidence that *Anindobothrium* is a member of the Rhinebothriidea. This results corroborate Ruhnke’s [3] observation that the presence of stalked bothridia found in members of *Anindobothrium* would support the assignment of this genus to this order (see also Healy *et al*. [2] and Ruhnke *et al*. [9]).

The sub-ordinal classification of the Rhinebothriidea, as we know to date, was proposed by Ruhnke *et al*. [8] which recognized four families: Anthocephaliidae Ruhnke, Caira & Cox, 2015, Echeneibothriidae, Escherbothriidae Ruhnke, Caira & Cox, 2015, and Rhinebothriidae Euzet, 1953. Despite the great taxonomic representation of their dataset, no specimens of *Anindobothrium* were included in their analysis. Our results revealed that the position of *Anindobothrium* is sensitive to optimality criterion since the MP topology suggested that this genus is sister to Anthocephaliidae, whereas the ML topology suggested that *Anindobothrium* is sister to the clade Anthocephaliidae+Escherbothriidae. Therefore, based on the phylogenetic patterns recovered, we would not be able to assign *Anindobothrium* to any family of the Rhinebothriidea. In addition, the amount of molecular divergence of the branch leading to haplotypes of *Anindobothrium* is comparable to those supporting families and the morphology of the genus that does not conform with the diagnoses of the families provided by Ruhnke *et al*. [8]. Hence these observations justify the erection of a new family to accommodate *Anindobothrium*.

The family Anindobothriidae fam. n. can be distinguished from the Echeneibothriidae by the absence of a myzorhynchus in the adult stage and by the position of genital pore (anterior vs. mid-posterior). It differs from the Rhinebothriidae by possessing a clear anteroposterior orientation of the bothridia characterized by a conspicuous apical sucker and by the partial or total interruption of the vitelline follicles by the ovary. It closely resembles the Anthocephaliidae and the Escherbothriidae but can be easily distinguished by the possession of post-vaginal testes. Below we provide a revised key of the Rhinebothriidea to accommodate the Anindobothriidae fam. n.

**Key to families of the Rhinebothriidea** (modified from Ruhnke *et al*. [8]):

1 Myzorhynchus present in the adult stage            Echeneibothriidae
- Myzorhynchus absent in the adult stage                    2
2 Bothridia lacking apical suckers and anterior/posterior orientation; vitelline follicles usually not interrupted by ovary                   Rhinebothriidae
- Bothridia with, or occasionally without, apical suckers and conspicuous anterior/posterior orientation; vitelline follicles with partial or total interruption by the ovary                                    3
3 Presence of post-vaginal testes                    Anindobothriidae
- Absence of post-vaginal testes                        4
4 Facial loculi arranged in anterior columns and posterior rows    Escherbothriidae
- Facial loculi lacking or arranged in multiple rows only        Anthocephaliidae

#### *Anindobothrium* Marques, Brooks & Lasso, 2001

**Amended Diagnosis**: Rhinebothriidea, Anindobothriidae fam. n. Worms acraspedote, apolytic. Scolex with four stalked bothridia; myzorhynchus absent. Bothridia typically longer than wide, with or without longitudinal septa, with apical sucker and marginal loculi, with or without two rows of facial loculi. Mature proglottids longer than wide. Testes numerous, arranged in two irregular columns; post-poral field present. Vas deferens extending anteriorly from mid-proglottid to enter to cirrus sac at anterior margin, more porally than anti-porally; external seminal vesicle absent. Genital pores marginal, irregularly alternating from 15 to 44% from anterior end of proglottid; genital atrium shallow. Cirrus sac in anterior $\frac{1}{4}$ of proglottid, thin-walled, tilted posteriorly, containing eversible coiled cirrus armed with spinitriches. Vagina extending from ootype along midline of proglottid to anterior margin of cirrus sac and laterally, becoming sinuous, to open into genital atrium anterior to cirrus sac; vaginal sphincter present; seminal receptacle absent. Ovary H-shaped in frontal view, tetralobed in cross section; ovarian margins lobulate. Vitellarium follicular, in two lateral bands; bands extending length of proglottid, interrupted by terminal genitalia, partial or total interruption by ovary. Uterus median, ventral, sacciform, with poorly differentiated lateral diverticula or lacking diverticula, extending from ovarian isthmus to anterior margin of proglottid. Excretory vessels four in number, arranged in dorsal and ventral pairs at lateral margins of proglottid. Parasites of the Potamotrygonidae (Myliobatiformes).

**Remarks**: The amended diagnosis made several contributions to that provided by Marques *et. al.* [4] and included modifications to accommodate the new findings on the morphology of this taxon. For instance, Marques *et al*. [4] described *Anindobothrium* as possessing bilobed bothridia, while in fact its members have elongated ones. Moreover, the absence of longitudinal septa became contradictory with the examination of additional specimens of *A. anacolum* (see below) and the presence of marginal loculi is found in all species we now recognize within the genus. Also, the vitelline follicles may be partial interrupted by the ovary. Since *Anindobothrium* is the only member of the Anindobothriidae fam. n., the genus can be differentiated from all other genera within the Rhinebothriidea by the same characters that differentiate the families of this order (see the key to the families above).

#### *Anindobothrium anacolum* (Brooks, 1977) Marques, Brooks & Lasso, 2001

**Fig 6:**
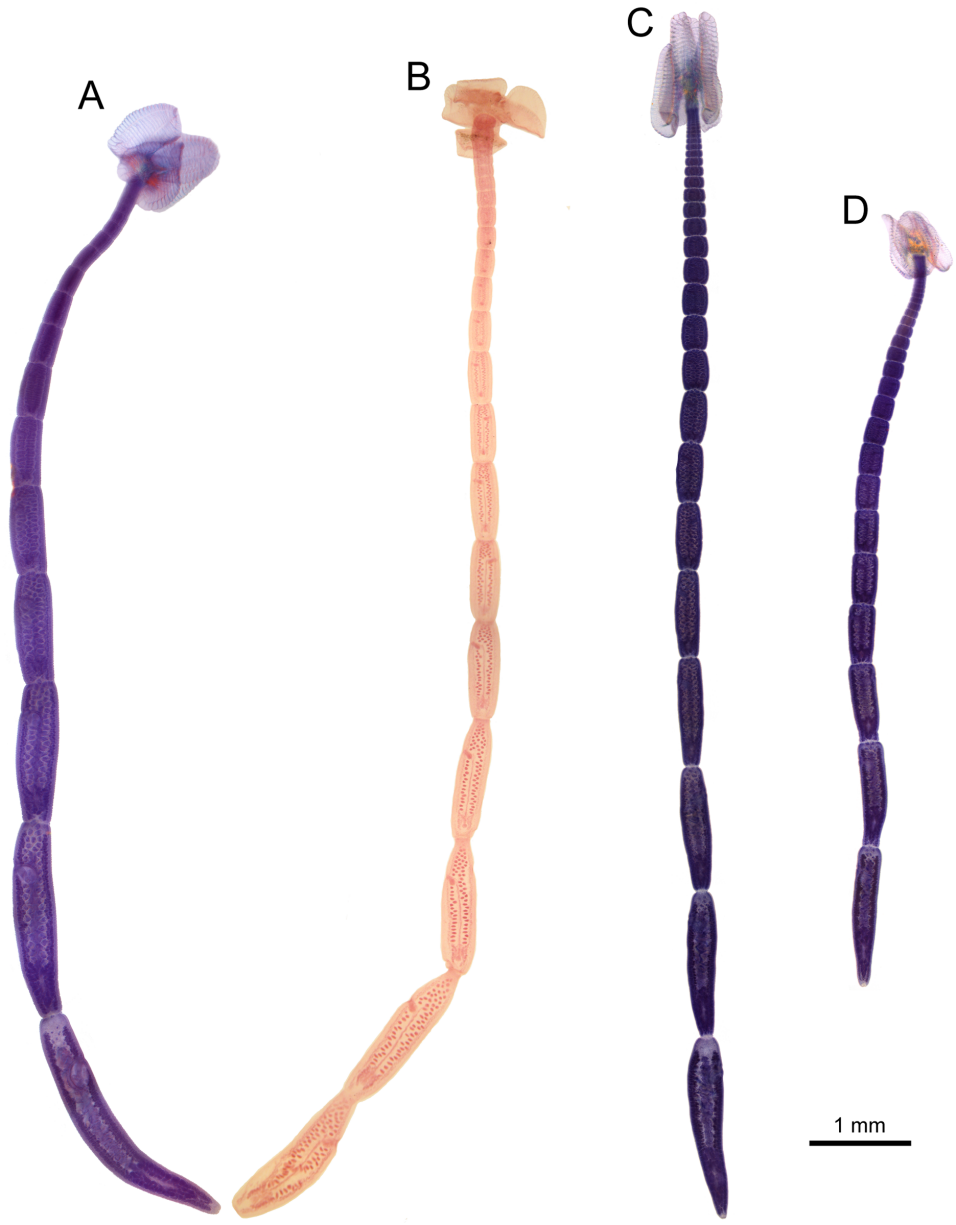
Light micrographs of the whole worms of *Anindobothrium* spp. A. *Anindobothrium anacolum* (Brooks, 1977) Marques, Brooks & Lasso, 2001 collected from the type locality (MZUSP 7937e, Voucher) ex *Styracura schmardae*. B. *A. lisae* (Brooks, 1977) Marques, Brooks & Lasso, 2001 (CHIOC 34375, holotype) ex *Potamotrygon orbignyi*. C. *A. inexpectatum* sp. n. (HWML 139137, paratype) ex *S. schmardae*. D. *A. carrioni* sp. n. (MIUP C-TET-PHY-A2, holotype) ex *S. pacifica*. Scale bar = 1 mm.

**Fig 7:**
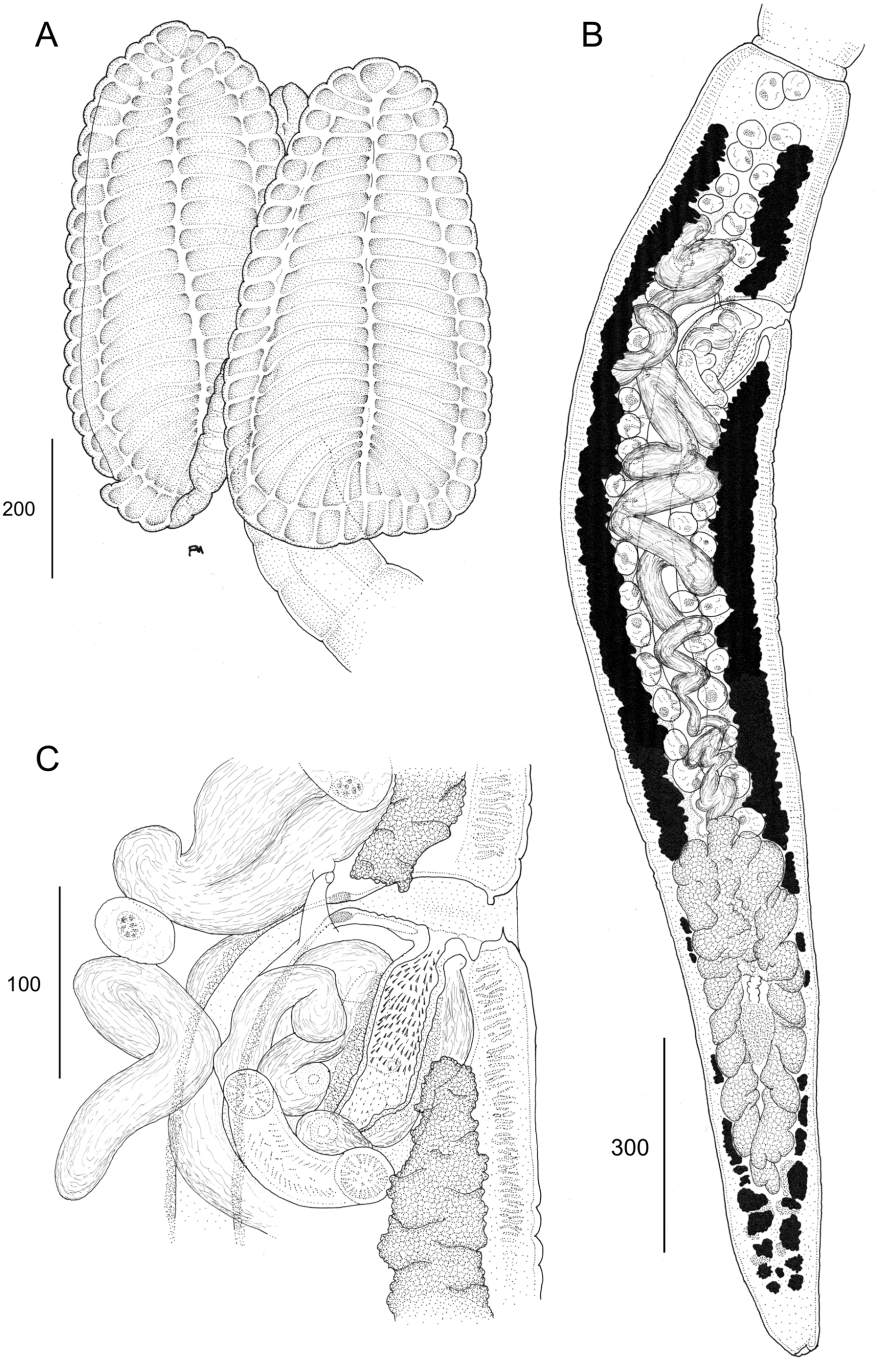
Line drawings of *Anindobothrium anacolum* ex *Styracura schmardae*. A. Scolex (MZUSP 7937f, Voucher). B. Terminal proglottid (HWML 139156, Voucher). C. Cirrus sac (LRP 9298, Voucher).

**Fig 8:**
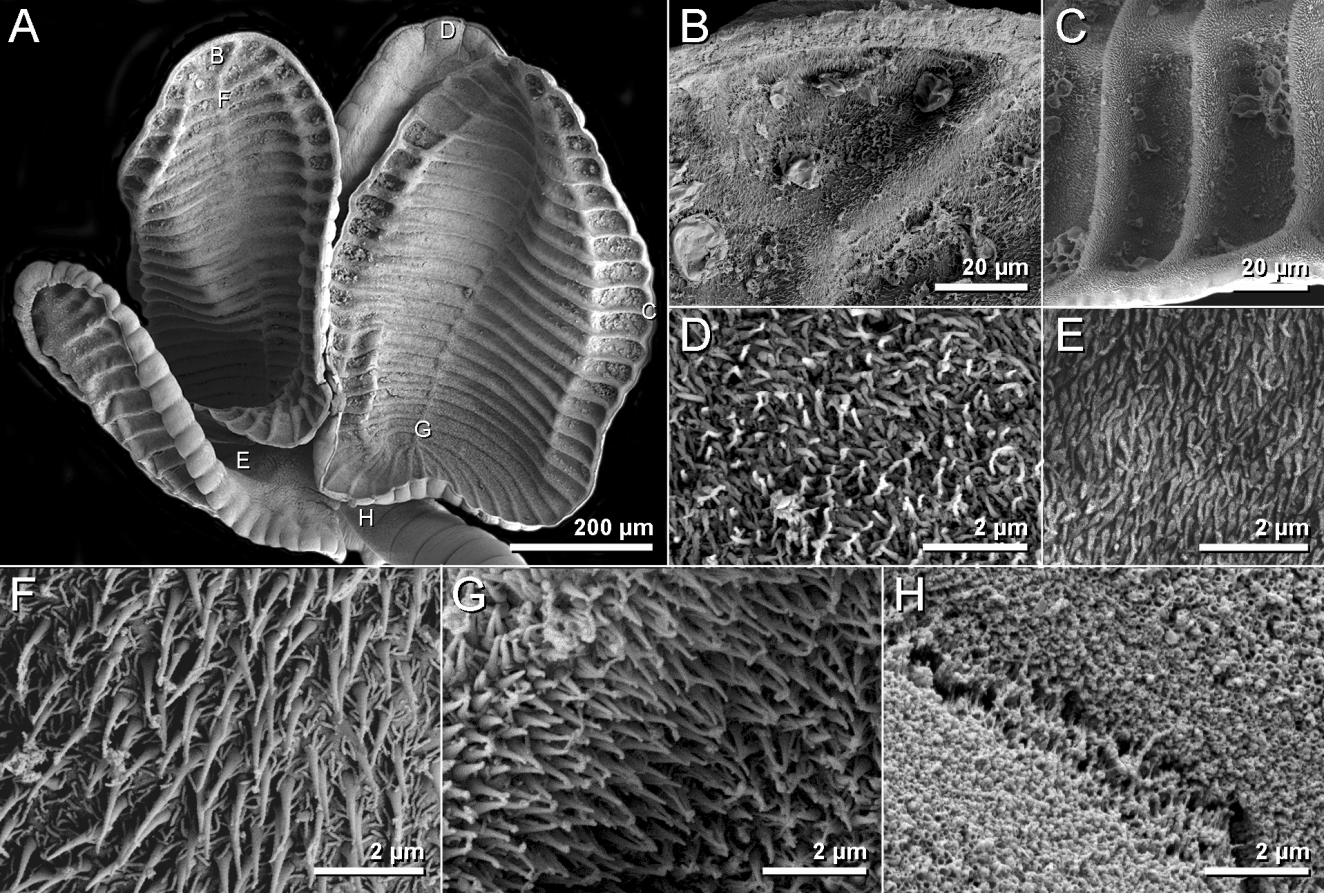
Scanning electron micrographs of *Anindobothrium anacolum* ex *Styracura schmardae*. A. scolex. B. distal surface of apical sucker. C. distal surface of medial loculi. D. proximal surface of apical sucker. E. proximal bothridial surface near centre of bothridium. F. distal anterior surface of longitunal septum. G. posterior portion of longitudinal septum on distal bothridial surface. H. cephalic peduncle.

**Fig 9:**
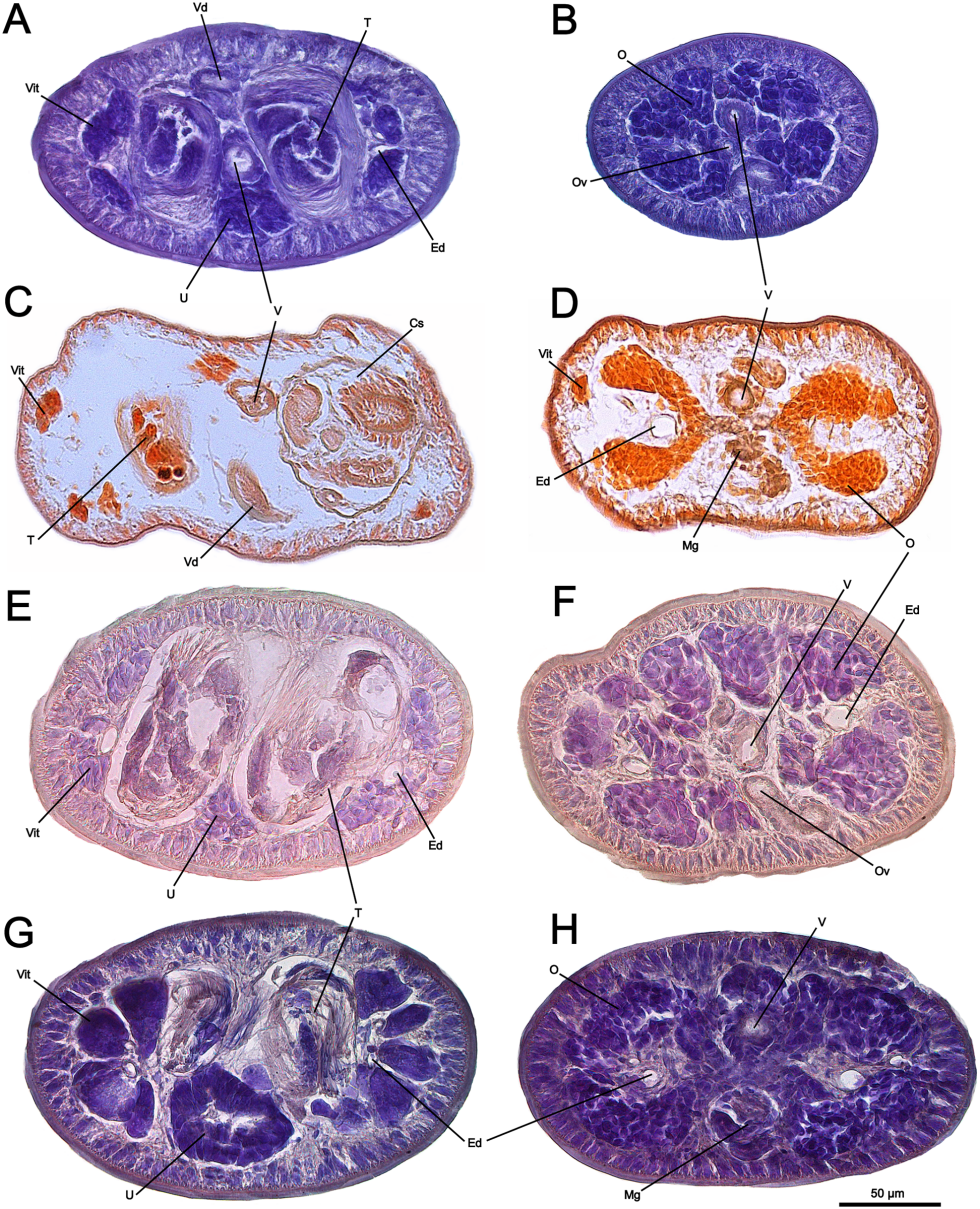
Micrographs of transversal histological sections of *Anindobothrium* spp. A,C,E,G. section at level of testes; B,D,F,H. section at level of ovary. *Anindobothrium anacolum* (A,B); *A. lisae* (C,D); *A. inexpectatum* sp. n. (E,F); e *A. carrioni* sp. n. (G,H). Abbreviations: Cs. cirrus sac; Ed. excretory duct; Mg. Mehlis’ gland; O. ovary; Ov. oviduct; T. testes; U. uterus; V. vagina; Vd. deferens vas; Vit. vitelline follicles.

(Figs 6A and 7–9A and 9B)

**Type host:** *Styracura schmardae* (Werner) de Carvalho, Loboda & da Silva (Myliobatiformes: Potamotrygonidae).

**Additional host:** *Potamotrygon yepezi* Castex & Castello (Myliobatiformes: Potamotrygonidae).

**Type locality:** Caribbean Sea, 15 km west of La Cienaga, Magdalena, Colombia (11°01’ N, 74°15’ W).

**Additional localities:** Caribbean Sea, Tasajeras, Magdalena, Colombia (10°58’ N, 74°19’ W and 11°00’ N, 74°16’ W); San Rafael de El Mojan, Lake Maracaibo, Maracaibo, Zulia, Venezuela (10°56’ N, 71°42’ W) and Maracas Bay, Maracas, San Juan-Laventille, Trinidad & Tobago (10°45’ N, 61°26’ W).

**Site of infection:** Spiral intestine.

**Type material:** holotype (USNPC 73969) and 4 paratypes (HWML 20265).

**Additional specimens deposited:** **MZUSP** 7778–7781, 7789 [molecular vouchers] and 7937a–7937g, 7938a–7938i [whole mounts]; **LRP** 9291–9305 [whole mounts], and **HWML** 139156–139173 [whole mounts].

**Redescription.** [Based on the type series comprised of holotype (USNPC 73969) and four paratypes (HWML 20265), and 66 additional mature specimens, which included 59 whole mounts, five worms observed with SEM, and two serially-sectioned]. Worms acraspedote, apolytic, 3.1–15.1 mm (63) long, composed of 8–33 (61) proglottids (Fig 6A). Scolex with greatest width 366–1,041 (45), composed of four stalked bothridia (Figs 6A, 7A and 8A). Bothridia elliptoid-shaped, 356–889 (62) long by 128–707 (62) wide, divided by 19–27 (50) transverse septa and one medial, longitudinal septum into 39–55 (50) facial loculi. Medial longitudinal septum extending from posterior margin of apical sucker to posterior margin of bothridium. Apical sucker 27–70 (51) long by 25–74 (49) wide (Fig 8B). Short cephalic peduncle. Proximal surface of apical sucker covered by acicular filitriches (Fig 8D) and medial portion of proximal bothridial surface covered by gladiate spinitriches (Fig 8E). Distal surfaces of bothridia covered by gladiate spinitriches and acicular filitriches (Fig 8C, 8F and 8G). Cephalic peduncle covered by capilliform filitriches (Fig 8H).

Immature proglottids wider than long. Mature proglottids 686–2,484 (63) long by 176–425 (63) wide, 1–7 (61) in number. Some terminal proglottids with sperm-filled vas deferens (Fig 7B). Gravid proglottids not observed. Testes round to oval, 32–96 (59) long by 23–79 (59) wide; 24–50 (59) in number; 2–8 pre-poral, 7–20 post-poral, 13–26 (59) anti-poral (Figs 6A and 9A). Cirrus sac in anterior $\frac{1}{4}$ of proglottid, round, 36–163 (63) long by 61–180 (63) wide, containing eversible coiled cirrus armed with spinitriches (Fig 7B and 7C). Genital atrium present. Genital pores 15–32% (63) of proglottid length from anterior end, irregularly alternating. The vagina runs anterior to the cirrus sac and then turns posteriorly towards the ootype. Ovary near posterior end of proglottid, bilobed in dorso-ventral view, tetra-lobed in cross-section (Figs 6A and 9B), symmetrical, 85–613 (63) long by 67–267 (63) wide at isthmus; anteroventral lobes converging anteriorly to midline of proglottid, but not fusing; ovarian margins lobulate. Vitelline follicles extending length of proglottid, 12–49 (53) long by 8–35 (53) wide; partial or total interruption by the ovary. Eggs not observed.

**Molecular diagnosis**: *Anindobothrium anacolum* may be differentiated from other members of the genus by the following set of 7 character states (base/position): **18S**—T/513; **Calmodulin**—A/242 and C/281; and **ITS**—A/226, T/340, G/341, and T/369. All character states are unique unambiguous synapomophies (see Fig 3C; Tables 5–7).

**Table 5:** Diagnostic sites of 18S, color shaded by species, based on unique and unambiguous synamoporphies for species of *Anindobothrium*. Site positions refer to alignment available in the repository Dryad under doi:10.5061/dryad.gr0sb.

Species\Site	163	251	259	510	513	981	985	1,008	1032	1,036	1,042	1,052	1,059	1,065	1,066
*A. inexpectatum* sp. n. [MZUSP 7767]	T	T	-	T	C	A	C	A	G	T	G	G	C	T	G
*A. inexpectatum* sp. n. [MZUSP 7768]	T	T	-	T	C	A	C	A	G	T	G	G	C	T	G
*A. inexpectatum* sp. n. [MZUSP 7769]	T	T	-	T	C	A	C	A	G	T	G	G	C	T	G
*A. inexpectatum* sp. n. [MZUSP 7770]	T	T	-	T	C	A	C	A	G	T	G	G	C	T	G
*A. inexpectatum* sp. n. [MZUSP 7771]	T	T	-	T	C	A	C	A	G	T	G	G	C	T	G
*A. inexpectatum* sp. n. [MZUSP 7772]	T	T	-	T	C	A	C	A	G	T	G	G	C	T	G
*A. inexpectatum* sp. n. [MZUSP 7773]	T	T	-	T	C	A	C	A	G	T	G	G	C	T	G
*A. inexpectatum* sp. n. [MZUSP 7774]	T	T	-	T	C	A	C	A	G	T	G	G	C	T	G
*A. inexpectatum* sp. n. [MZUSP 7777]	T	T	-	T	C	A	C	A	G	T	G	G	C	T	G
*A. inexpectatum* sp. n. [MZUSP 7775]	T	T	-	T	C	A	C	A	G	T	G	G	C	T	G
*A. inexpectatum* sp. n. [MZUSP 7776]	T	T	-	T	C	A	C	A	G	T	G	G	C	T	G
*A. anacolum* [MZUSP 7789]	T	T	-	T	T	A	C	A	A	T	G	G	C	T	G
*A. anacolum* [MZUSP 7778]	T	T	-	T	T	A	C	A	A	T	G	G	C	T	G
*A. anacolum* [MZUSP 7779]	T	T	-	T	T	A	C	A	A	T	G	G	C	T	G
*A. anacolum* [MZUSP 7780]	T	T	-	T	T	A	C	A	A	T	G	G	C	T	G
*A. anacolum* [MZUSP 7781]	T	T	-	T	T	A	C	A	A	T	G	G	C	T	G
*A. carrioni* sp. n. [MZUSP 7787]	T	T	-	T	C	A	C	A	A	T	A	G	C	T	G
*A. carrioni* sp. n. [MZUSP 7788]	T	T	-	T	C	A	C	A	A	T	A	G	C	T	G
*A. carrioni* sp. n. [MZUSP 7785]	T	T	-	T	C	A	C	A	A	T	A	G	C	T	G
*A. carrioni* sp. n. [MZUSP 7786]	T	T	-	T	C	A	C	A	A	T	A	G	C	T	G
*A. lisae* [MZUSP 7783]	C	C	G	C	G	G	A	G	-	C	G	T	T	C	A
*A. lisae* [MZUSP 7782]	C	C	G	C	G	G	A	G	-	C	G	T	T	C	A
*A. lisae* [MZUSP 7784]	C	C	G	C	G	G	A	G	-	C	G	T	T	C	A
*Anth. hobergi* [MZUSP 7756]	T	T	-	T	C	A	C	A	A	T	G	G	C	T	G

**Table 6:** Diagnostic sites of Calmodulin, color shaded by species, based on unique and unambiguous synamoporphies for species of *Anindobothrium*. Site positions (bold) refer to alignment available in the repository Dryad under doi:10.5061/dryad.gr0sb.

Species\Site	74	122	242	281	307	314
*A. inexpectatum* sp. n. [MZUSP 7767]	G	G	G	T	-	-
*A. inexpectatum* sp. n. [MZUSP 7768]	G	G	G	T	-	-
*A. inexpectatum* sp. n. [MZUSP 7769]	G	G	G	T	-	-
*A. inexpectatum* sp. n. [MZUSP 7770]	G	G	G	T	-	-
*A. inexpectatum* sp. n. [MZUSP 7771]	G	G	G	T	-	-
*A. inexpectatum* sp. n. [MZUSP 7772]	G	G	G	T	-	-
*A. inexpectatum* sp. n. [MZUSP 7773]	G	G	G	T	-	-
*A. inexpectatum* sp. n. [MZUSP 7774]	G	G	G	T	-	-
*A. inexpectatum* sp. n. [MZUSP 7777]	G	G	G	T	-	-
*A. inexpectatum* sp. n. [MZUSP 7775]	G	G	G	A	-	A
*A. inexpectatum* sp. n. [MZUSP 7776]	G	G	G	T	-	-
*A. anacolum* [MZUSP 7789]	G	G	A	C	-	-
*A. anacolum* [MZUSP 7778]	G	G	A	C	-	-
*A. anacolum* [MZUSP 7779]	G	G	A	C	-	-
*A. anacolum* [MZUSP 7780]	G	G	A	C	-	-
*A. anacolum* [MZUSP 7781]	G	G	A	C	-	-
*A. carrioni* sp. n. [MZUSP 7787]	G	A	G	T	T	-
*A. carrioni* sp. n. [MZUSP 7788]	G	A	G	T	T	-
*A. carrioni* sp. n. [MZUSP 7785]	G	A	G	T	T	-
*A. carrioni* sp. n. [MZUSP 7786]	G	A	G	T	T	-
*A. lisae* [MZUSP 7783]	A	G	G	G	-	C
*A. lisae* [MZUSP 7782]	A	G	G	G	-	C
*A. lisae* [MZUSP 7784]	A	G	G	G	-	C
*Anth. hobergi* [MZUSP 7756]	G	C	G	A	-	-

**Table 7:** Diagnostic sites of ITS, color shaded by species, based on unique and unambiguous synamoporphies for species of *Anindobothrium*. Site positions (bold) refer to alignment available in the repository Dryad under doi:10.5061/dryad.gr0sb.

**Species\Site**	**82**	**83**	**137**	**146**	**157**	**203**	**204**	**213**	**218**	**223**	**226**	**229**	**244**	**254**	**277**	**281**	**282**	**309**	**339**	**340**	**341**	**355**
*A. inexpectatum* sp. n. [MZUSP 7767]	T	A	T	T	-	A	A	A	C	-	-	C	-	-	A	G	-	A	C	-	-	G
*A. inexpectatum* sp. n. [MZUSP 7768]	T	A	T	T	-	A	A	A	C	-	-	C	-	-	A	G	-	A	C	-	-	G
*A. inexpectatum* sp. n. [MZUSP 7769]	T	A	T	T	-	A	A	A	C	-	-	C	-	-	A	G	-	A	C	-	-	G
*A. inexpectatum* sp. n. [MZUSP 7770]	T	A	T	T	-	A	A	A	C	-	-	C	-	-	A	G	-	A	C	-	-	G
*A. inexpectatum* sp. n. [MZUSP 7771]	T	A	T	T	-	A	A	A	C	-	-	C	-	-	A	G	-	A	C	-	-	G
*A. inexpectatum* sp. n. [MZUSP 7772]	T	A	T	T	-	A	A	A	C	-	-	C	-	-	A	G	-	A	C	-	-	G
*A. inexpectatum* sp. n. [MZUSP 7773]	T	A	T	T	-	A	A	A	C	-	-	C	-	-	A	G	-	A	C	-	-	G
*A. inexpectatum* sp. n. [MZUSP 7774]	T	A	T	T	-	A	A	A	C	-	-	C	-	-	A	G	-	A	C	-	-	G
*A. inexpectatum* sp. n. [MZUSP 7777]	T	A	T	T	-	A	A	A	C	-	-	C	-	-	A	G	-	A	C	-	-	G
*A. inexpectatum* sp. n. [MZUSP 7775]	T	A	T	T	-	A	A	A	C	-	-	C	-	-	A	G	-	A	C	-	-	G
*A. inexpectatum* sp. n. [MZUSP 7776]	T	A	T	T	-	A	A	A	C	-	-	C	-	-	A	G	-	A	C	-	-	G
*A. anacolum* [MZUSP 7789]	-	-	T	T	-	-	-	A	C	-	A	G	-	-	A	G	-	A	C	T	G	C
*A. anacolum* [MZUSP 7778]	-	-	T	T	-	-	-	A	C	-	A	G	-	-	A	G	-	A	C	T	G	C
*A. anacolum* [MZUSP 7779]	-	-	T	T	-	-	-	A	C	-	A	G	-	-	A	G	-	A	C	T	G	C
*A. anacolum* [MZUSP 7780]	-	-	T	T	-	-	-	A	C	-	A	G	-	-	A	G	-	A	C	T	G	C
*A. anacolum* [MZUSP 7781]	-	-	T	T	-	-	-	A	C	-	A	G	-	-	A	G	-	A	C	T	G	C
*A. carrioni* sp. n. [MZUSP 7787]	-	-	T	T	-	-	-	T	C	-	-	G	-	-	G	A	-	A	T	-	-	T
*A. carrioni* sp. n. [MZUSP 7788]	-	-	T	T	-	-	-	T	C	-	-	G	-	-	G	A	-	A	T	-	-	T
*A. carrioni* sp. n. [MZUSP 7785]	-	-	T	T	-	-	-	T	C	-	-	G	-	-	G	A	-	A	T	-	-	T
*A. carrioni* sp. n. [MZUSP 7786]	-	-	T	T	-	-	-	T	C	-	-	G	-	-	G	A	-	A	T	-	-	T
*A. lisae* [MZUSP 7783]	-	-	C	C	T	-	-	A	G	C	-	-	A	T	-	G	T	T	C	-	-	C
*A. lisae* [MZUSP 7782]	-	-	C	C	T	-	-	A	G	C	-	-	A	T	-	G	T	T	C	-	-	C
*A. lisae* [MZUSP 7784]	-	-	C	C	T	-	-	A	G	C	-	-	A	T	-	G	T	T	C	-	-	C
*Anth. hobergi* [MZUSP 7756]	-	-	T	T	-	-	-	A	C	-	-	G	G	-	A	G	-	A	C	-	-	-
**Species\Site**	**369**	**370**	**372**	**417**	**418**	**435**	**461**	**463**	**464**	**468**	**469**	**484**	**495**	**515**	**548**	**552**	**639**	**653**	**722**	**746**	**774**	
*A. inexpectatum* sp. n. [MZUSP 7767]	G	T	C	T	A	-	G	T	G	-	-	C	T	-	A	T	G	T	C	A	-	
*A. inexpectatum* sp. n. [MZUSP 7768]	G	T	C	T	A	-	G	T	G	-	-	C	T	-	A	T	G	T	C	A	-	
*A. inexpectatum* sp. n. [MZUSP 7769]	G	T	C	T	A	-	G	T	G	-	-	C	T	-	A	T	G	T	C	A	-	
*A. inexpectatum* sp. n. [MZUSP 7770]	G	T	C	T	A	-	G	T	G	-	-	C	T	-	A	T	G	T	C	A	-	
*A. inexpectatum* sp. n. [MZUSP 7771]	G	T	C	T	A	-	G	T	G	-	-	C	T	-	A	T	G	T	C	A	-	
*A. inexpectatum* sp. n. [MZUSP 7772]	G	T	C	T	A	-	G	T	G	-	-	C	T	-	A	T	G	T	C	A	-	
*A. inexpectatum* sp. n. [MZUSP 7773]	G	T	C	T	A	-	G	T	G	-	-	C	T	-	A	T	G	T	C	A	-	
*A. inexpectatum* sp. n. [MZUSP 7774]	G	T	C	T	A	-	G	T	G	-	-	C	T	-	A	T	G	T	C	A	-	
*A. inexpectatum* sp. n. [MZUSP 7777]	G	T	C	T	A	-	G	T	G	-	-	C	T	-	A	T	G	T	C	A	-	
*A. inexpectatum* sp. n. [MZUSP 7775]	G	T	C	T	A	-	G	T	G	-	-	C	T	-	A	T	G	T	C	A	-	
*A. inexpectatum* sp. n. [MZUSP 7776]	G	T	C	T	A	-	G	T	G	-	-	C	T	-	A	T	G	T	C	A	-	
*A. anacolum* [MZUSP 7789]	T	T	-	-	-	-	G	T	G	-	-	C	T	-	G	T	G	T	C	A	-	
*A. anacolum* [MZUSP 7778]	T	T	-	-	-	-	G	T	G	-	-	C	T	-	G	T	G	T	C	A	-	
*A. anacolum* [MZUSP 7779]	T	T	-	-	-	-	G	T	G	-	-	C	T	-	G	T	G	T	C	A	-	
*A. anacolum* [MZUSP 7780]	T	T	-	-	-	-	G	T	G	-	-	C	T	-	G	T	G	T	C	A	-	
*A. anacolum* [MZUSP 7781]	T	T	-	-	-	-	G	T	G	-	-	C	T	-	G	T	G	T	C	A	-	
*A. carrioni* sp. n. [MZUSP 7787]	G	T	-	-	-	-	T	T	C	-	-	C	T	-	G	C	G	C	T	A	A	
*A. carrioni* sp. n. [MZUSP 7788]	G	T	-	-	-	-	T	T	C	-	-	C	T	-	G	C	G	C	T	A	A	
*A. carrioni* sp. n. [MZUSP 7785]	G	T	-	-	-	-	T	T	C	-	-	C	T	-	G	C	G	C	T	A	A	
*A. carrioni* sp. n. [MZUSP 7786]	G	T	-	-	-	-	T	T	C	-	-	C	T	-	G	C	G	C	T	A	A	
*A. lisae* [MZUSP 7783]	G	G	-	-	-	G	G	C	G	T	A	T	C	T	G	T	A	T	C	G	-	
*A. lisae* [MZUSP 7782]	G	G	-	-	-	G	G	C	G	T	A	T	C	T	G	T	A	T	C	G	-	
*A. lisae* [MZUSP 7784]	G	G	-	-	-	G	G	C	G	T	A	T	C	T	G	T	A	T	C	G	-	
*Anth. hobergi* [MZUSP 7756]	G	T	-	-	-	-	G	T	-	-	-	C	T	-	G	-	G	G	C	A	-	

**Remarks.** The acquisition of additional specimens from the type locality and other localities in the Caribbean Sea and adjacent waters (*i.e.*, Lake Maracaibo) allowed us to better understand the distribution, host association, and morphological variability of *Anindobothrium anacolum*. In addition, we provided for the first time the description of the microtriches morphology and a molecular diagnosis for this species.

*Anindobothrium anacolum* seems to have a restricted distribution in the Southern Caribbean Sea. Among all localities sampled in the Caribbean Sea, which included the coasts of Belize, Panama, Colombia and Trinidad & Tobago, we were only able to collect this species off the coasts of the last two countries. Furthermore, we also collected few specimens from Lake Maracaibo in Maracaibo infecting *Potamotrygon yepezi*, a freshwater potamotrygonid.

The presence of *A. anacolum* in *Potamotrygon yepezi* was unexpected. This species was only known to parasitize *S. schmardae* and most members of the Rhinebothriidea seems to exhibit oioxenous specificity for their hosts (sensu Euzet & Combes [57]; Ruhnke *et al*. [8]). Be that as it may, this could represent an accidental infection that needs further investigation.

The examination of the type series and additional material revealed that the original description provided by Brooks [58] as well as the diagnosis of this species amended by Marques *et al*. [4] did not provide a fair account on the bothridial morphology of this taxon. Both previous studies provided a description of the bothridia based on the holotype (USNPC 73969), which was poorly prepared and did not permit the verification of the presence of a longitudinal septum and the marginal loculi on the bothridia. Moreover, Marques *et al*. [4] illustrated a poorly defined anterior marginal loculus (figure 1 in [4]), which we considered as an apical sucker. Therefore, in the present redescription, bothridial structures were observed and described in newly collected specimens.

The additional material provided a better understanding of the morphological variability of this species. Their examination allowed us to increase the range of some structures, which revealed to be more variable than previously reported. Both previous studies found that the total length was 6.8–15.4 but we found it to be 3.1–15.1. Brooks [58] observed 23–24 loculi, while Marques *et al*. [4] found between 42 and 44. The present study revealed a number of loculi of 39–55, which closely corresponds to the range reported by Marques *et al*. [4]. This difference could be attributed to the presence of longitudinal septa, not seen by Brooks in 1977. For genital pore position, both studies reported it to be 19–29% from anterior end, whereas, we found 15–32%.

*Anindobothrium anacolum* can be easily differentiated from the only species previously assigned to the genus, *A. lisae*, by the morphology of the bothridia. *Anindobothrium anacolum* possesses marginal and facial loculi due to the presence of a longitudinal septum and transversal septa on bothridia, whereas *A. lisae* has only marginal loculi. As will be shown below, all marine species recognized for *Anindobothrium* possess bothridial architecture similar to *A. anacolum*, hence can be likewise differentiated from *A. lisae*. Despite the differences in the bothridial morphology, the proglottids of both species are similar by possessing numerous testes arranged in two irregular columns with post-poral field present, cirrus sac at the anterior $\frac{1}{4}$ of proglottid, ovary H-shaped in frontal view, follicular vitelaria arranged in two lateral bands extending the length of proglottid, which is interrupted by terminal genitalia and partial or total interruption by the ovary. Finally, *A. anacolum* can be distinguished from all species of the genus by a set of 7 molecular synapomorphies (Fig 3C; Tables 5–7).

#### *Anindobothrium lisae* Marques, Brooks & Lasso, 2001

**Fig 10:**
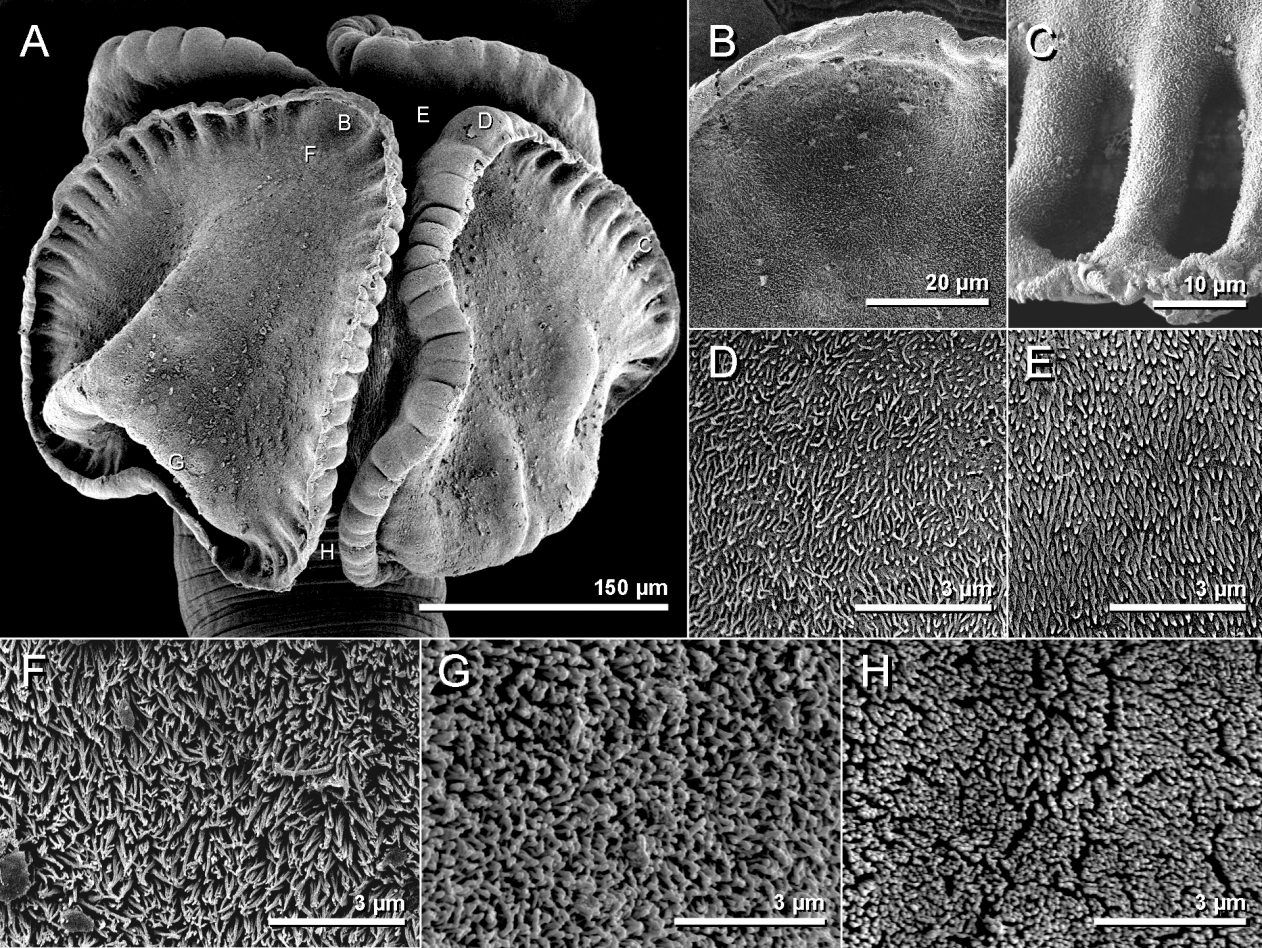
Scanning electron micrographs of *Anindobothrium lisae* ex *Potamotrygon orbignyi*. A. scolex. B. distal surface of apical sucker. C. distal surface of medial loculi. D. proximal surface of apical sucker. E. proximal bothridial surface near centre of bothridium. F. distal anterior bothridia surface. G. distal bothridia posterior surface. H. cephalic peduncle.

(Figs 6B, 9C and 9D and 10)

**Type host:** *Potamotrygon orbignyi* (Castelnau) (Myliobatiformes: Potamotrygonidae).

**Additional host:** *Potamotrygon schroederi* Fernández-Yépez (Myliobatiformes: Potamotrygonidae).

**Type locality:** Rio Negro, near Barcelos, Amazonas, Brazil (00°59’S, 62°58’W).

**Additional locality:** Rio Negro, near Barcelos, Amazonas, Brazil (00°54’ S, 62°58’ W and 00°98’ S, 62°92’ W); Rio Apure, Muñoz, Apure, Venezuela (7°53’ N, 68°52’ W).

**Site of infection:** Spiral valve.

**Type material:** holotype (CHIOC 34375) and 3 paratypes (HWML 16379a and INPA 400a,b).

**Additional specimens deposited:** **MZUSP** 7782–7784 [molecular vouchers], 7939a–7939d [whole mounts]; **LRP** 9306–9310 [whole mounts]; and **HWML** 139174–139185 [whole mounts].

**Resdescription.** [Based on the type series comprised of holotype (CHIOC 34375) and three paratypes (HWML 16379a and INPA 400a,b), and 34 additional mature specimens, which included 29 whole mounts, three worms observed with SEM, and two serially-sectioned]: Worms acraspedote, apolytic, 2.5–11.7 mm (25) long, composed of 7–24 (26) proglottids (Fig 6B). Scolex with greatest width 386–1,197 (32), composed of four, stalked bothridia (Figs 6B and 10A). Bothridia orbicular-elliptoid shaped, 225–643 (17) long by 297–838 (12) wide, with 40–58 (10) marginal loculi and an apical sucker, 20–79 (8) long by 30–79 (9) wide (Fig 10B and 10C). Transverse and longitudinal septa absent. Short cephalic peduncle. Proximal surface of apical sucker covered by acicular filitriches (Fig 10D); medial portion of proximal bothridial surface covered by gladiate spinitriches (Fig 10E). Distal surfaces of bothridia covered by acicular filitriches (Fig 10C, 10F and 10G), by papilliform (Fig 10C) and capilliform filitriches (Fig 10F). Cephalic peduncle covered by capilliform filitriches (Fig 10H).

Immature proglottids wider than long. Mature proglottids 772–2,395 (28) long by 257–643 (28) wide, 1–4 (26) in number. Vas deferens not sperm-filled in terminal proglottids. Gravid proglottids not observed. Testes round to oval, 44–101 (27) long by 19–61 (27) wide; 30–72 (27) in number; 3–15 pre-poral, 10–21 post-poral and 15–43 (27) anti-poral (Figs 6B and 9C). Cirrus sac pyriform shaped in anterior $\frac{1}{4}$ of proglottid, 45–130 (27) long by 65–212 (27) wide, containing eversible coiled cirrus armed with spinitriches (Figs 6B and 9C). Genital atrium present. Genital pores 18–44% (27) of proglottid length from anterior end, irregularly alternating. The vagina runs anterior to the cirrus sac and then turns posteriorly towards the ootype. Ovary near posterior end of proglottid, billobed in dorso-ventral view, tetra-lobed in cross-section (Figs 6B and 9D), symmetrical, 55–159 (26) long by 138–385 (26) wide at isthmus; anteroventral lobes converging anteriorly to midline of proglottid, but not fused; ovarian margins lobulate. Vitelline follicles extending length of proglottid, 10–49 (27) long by 7–27 (27) wide; which could be partially interrupted by the ovary. Eggs not observed.

**Molecular diagnosis**: *Anindobothrium lisae* may be differentiated from other members of the genus by the following set of 67 character states (base/position): **COI**—A/57, G/61, C/117, T/123, G/153, C/184, T/195, T/219, T/229, T/242, A/243, G/255, T/339, A/382, A/431, T/472, T/495, T/504, and A/528; **18S**—C/163, C/251, G/259, C/510, G/513, G/981, A/985, G/1,008, C/1,036, T/1,052, T/1,059, C/1,065, A/1,066; **28S**—G/371, G/647, G/661, T/768, insertion of 3 base pairs between positions 1041 and 1045 with 2 synapomorphies—G/1,043 and A/1,044, insertion of 113 base pairs between positions 1,094 and 1,106 with 7 synapomorphies—C/1,095, G/1,097, G/1,100, A/1,102, G/1,103, A/1,104, and G/1,105 (see Table 8); **Calmodulin**—A/74, C/314; and **ITS**—C/137, C/146, T/157, G/218, C/223, A/244, T/254, T/282, T/309, G/370, G/435, C/463, T/468, A/469, C/476, T/484, C/495, T/515, A/639, G/746. All character states are unique unambiguous synapomophies (see Fig 3B and 3C; Tables 5–9).

**Table 8:** Diagnostic sites of 28S, color shaded by species, based on unique and unambiguous synamoporphies for species of *Anindobothrium*. Site positions (bold) refer to alignment available in the repository Dryad under doi:10.5061/dryad.gr0sb. Sites in gray indicate place holding positions for INDELs.

**Species\Site**	**371**	**591**	**647**	**661**	**768**	1,040	1,041	1,042	**1,043**	**1,044**	1,045	1,046	1,093
*A. inexpectatum* sp. n. [MZUSP 7767]	A	G	-	A	C	G	A	-	-	-	C	T	T
*A. inexpectatum* sp. n. [MZUSP 7768]	A	G	-	A	C	G	A	-	-	-	C	T	T
*A. inexpectatum* sp. n. [MZUSP 7769]	A	G	-	A	C	G	A	-	-	-	C	T	T
*A. inexpectatum* sp. n. [MZUSP 7770]	A	G	-	A	C	G	A	-	-	-	C	T	T
*A. inexpectatum* sp. n. [MZUSP 7771]	A	G	-	A	C	G	A	-	-	-	C	T	T
*A. inexpectatum* sp. n. [MZUSP 7772]	A	G	-	A	C	G	A	-	-	-	C	T	T
*A. inexpectatum* sp. n. [MZUSP 7773]	A	G	-	A	C	G	A	-	-	-	C	T	T
*A. inexpectatum* sp. n. [MZUSP 7774]	A	G	-	A	C	G	A	-	-	-	C	T	T
*A. inexpectatum* sp. n. [MZUSP 7777]	A	G	-	A	C	G	A	-	-	-	C	T	T
*A. inexpectatum* sp. n. [MZUSP 7775]	A	G	-	A	C	G	A	-	-	-	C	T	T
*A. inexpectatum* sp. n. [MZUSP 7776]	A	G	-	A	C	G	A	-	-	-	C	T	T
*A. anacolum* [MZUSP 7789]	A	G	-	A	C	G	A	-	-	-	C	T	T
*A. anacolum* [MZUSP 7778]	A	G	-	A	C	G	A	-	-	-	C	T	T
*A. anacolum* [MZUSP 7779]	A	G	-	A	C	G	A	-	-	-	C	T	T
*A. anacolum* [MZUSP 7780]	A	G	-	A	C	G	A	-	-	-	C	T	T
*A. anacolum* [MZUSP 7781]	A	G	-	A	C	G	A	-	-	-	C	T	T
*A. carrioni* sp. n. [MZUSP 7787]	A	A	-	A	C	G	A	-	-	-	C	T	T
*A. carrioni* sp. n. [MZUSP 7788]	A	A	-	A	C	G	A	-	-	-	C	T	T
*A. carrioni* sp. n. [MZUSP 7785]	A	A	-	A	C	G	A	-	-	-	C	T	T
*A. carrioni* sp. n. [MZUSP 7786]	A	A	-	A	C	G	A	-	-	-	C	T	T
*A. lisae* [MZUSP 7783]	G	G	G	G	T	G	A	C	G	A	C	T	T
*A. lisae* [MZUSP 7782]	G	G	G	G	T	G	A	C	G	A	C	T	T
*A. lisae* [MZUSP 7784]	G	G	G	G	T	G	A	C	G	A	C	T	T
*Anth. hobergi* [MZUSP 7756]	A	G	-	A	C	G	C	C	-	-	C	T	T
**Species\Site**	1,094	**1095**	1,096	**1097**	1,098	1,099	**1100**	1,101	**1,102**	**1,103**	**1,104**	**1,105**	1,106
*A. inexpectatum* sp. n. [MZUSP 7767]	T	-	-	-	-	-	-	-	-	-	-	-	C
*A. inexpectatum* sp. n. [MZUSP 7768]	T	-	-	-	-	-	-	-	-	-	-	-	C
*A. inexpectatum* sp. n. [MZUSP 7769]	T	-	-	-	-	-	-	-	-	-	-	-	C
*A. inexpectatum* sp. n. [MZUSP 7770]	T	-	-	-	-	-	-	-	-	-	-	-	C
*A. inexpectatum* sp. n. [MZUSP 7771]	T	-	-	-	-	-	-	-	-	-	-	-	C
*A. inexpectatum* sp. n. [MZUSP 7772]	T	-	-	-	-	-	-	-	-	-	-	-	C
*A. inexpectatum* sp. n. [MZUSP 7773]	T	-	-	-	-	-	-	-	-	-	-	-	C
*A. inexpectatum* sp. n. [MZUSP 7774]	T	-	-	-	-	-	-	-	-	-	-	-	C
*A. inexpectatum* sp. n. [MZUSP 7777]	T	-	-	-	-	-	-	-	-	-	-	-	C
*A. inexpectatum* sp. n. [MZUSP 7775]	T	-	-	-	-	-	-	-	-	-	-	-	C
*A. inexpectatum* sp. n. [MZUSP 7776]	T	-	-	-	-	-	-	-	-	-	-	-	C
*A. anacolum* [MZUSP 7789]	T	-	-	-	-	-	-	-	-	-	-	-	C
*A. anacolum* [MZUSP 7778]	T	-	-	-	-	-	-	-	-	-	-	-	C
*A. anacolum* [MZUSP 7779]	T	-	-	-	-	-	-	-	-	-	-	-	C
*A. anacolum* [MZUSP 7780]	T	-	-	-	-	-	-	-	-	-	-	-	C
*A. anacolum* [MZUSP 7781]	T	-	-	-	-	-	-	-	-	-	-	-	C
*A. carrioni* sp. n. [MZUSP 7787]	T	-	-	-	-	-	-	-	-	-	-	-	C
*A. carrioni* sp. n. [MZUSP 7788]	T	-	-	-	-	-	-	-	-	-	-	-	C
*A. carrioni* sp. n. [MZUSP 7785]	T	-	-	-	-	-	-	-	-	-	-	-	C
*A. carrioni* sp. n. [MZUSP 7786]	T	-	-	-	-	-	-	-	-	-	-	-	C
*A. lisae* [MZUSP 7783]	T	C	A	G	C	A	G	C	A	G	A	G	C
*A. lisae* [MZUSP 7782]	T	C	-	G	-	-	G	C	A	G	A	G	C
*A. lisae* [MZUSP 7784]	T	C	-	G	-	-	G	C	A	G	A	G	C
*Anth. hobergi* [MZUSP 7756]	G	-	-	-	-	-	-	C	-	-	-	-	C

**Table 9:** Diagnostic sites of COI based on unique and unambiguous synamoporphies for species of *Anindobothrium*. Site positions refer to alignment available in the repository Dryad under doi:10.5061/dryad.gr0sb.

**Species\Site**	**57**	**61**	**105**	**117**	**123**	**141**	**153**	**168**	**184**	**195**	**219**	**229**	**242**	**243**	**250**
*A. inexpectatum* sp. n. [MZUSP 7767]	T	A	C	T	C	A	T	T	T	A	G	C	C	T	T
*A. inexpectatum* sp. n. [MZUSP 7768]	T	A	C	T	C	A	T	T	T	A	G	C	C	T	T
*A. inexpectatum* sp. n. [MZUSP 7769]	T	A	C	T	C	A	T	T	T	A	G	C	C	T	T
*A. inexpectatum* sp. n. [MZUSP 7770]	T	A	C	T	C	A	T	T	T	A	G	C	C	T	T
*A. inexpectatum* sp. n. [MZUSP 7771]	T	A	C	T	C	A	T	T	T	A	G	C	C	T	T
*A. inexpectatum* sp. n. [MZUSP 7772]	T	A	C	T	C	A	T	T	T	A	G	C	C	T	T
*A. inexpectatum* sp. n. [MZUSP 7773]	T	A	C	T	C	A	T	T	T	A	G	C	C	T	T
*A. inexpectatum* sp. n. [MZUSP 7774]	T	A	C	T	C	A	T	T	T	A	G	C	C	T	T
*A. inexpectatum* sp. n. [MZUSP 7777]	T	T	C	T	C	A	T	C	T	A	G	C	C	T	T
*A. inexpectatum* sp. n. [MZUSP 7775]	T	A	C	T	C	A	T	T	T	A	G	C	C	T	T
*A. inexpectatum* sp. n. [MZUSP 7776]	T	A	C	T	C	A	T	T	T	A	G	C	C	T	T
*A. anacolum* [MZUSP 7789]	T	A	A	T	C	A	A	T	T	A	G	C	C	T	T
*A. anacolum* [MZUSP 7778]	T	A	T	T	C	A	A	T	T	A	G	C	C	T	T
*A. anacolum* [MZUSP 7779]	T	A	A	T	C	A	A	T	T	A	G	C	C	T	T
*A. anacolum* [MZUSP 7780]	T	A	T	T	C	A	T	T	T	A	G	C	C	T	T
*A. anacolum* [MZUSP 7781]	T	A	T	T	C	A	T	T	T	A	G	C	C	T	T
*A. carrioni* sp. n. [MZUSP 7787]	T	A	A	T	C	T	A	A	T	A	C	C	C	T	C
*A. carrioni* sp. n. [MZUSP 7788]	T	A	A	T	C	T	A	A	T	A	C	C	C	T	C
*A. carrioni* sp. n. [MZUSP 7785]	T	A	A	T	C	T	A	A	T	A	C	C	C	T	C
*A. carrioni* sp. n. [MZUSP 7786]	T	A	A	T	C	T	A	A	T	A	C	C	C	T	C
*A. lisae* [MZUSP 7783]	A	G	T	C	T	A	G	T	C	T	T	T	T	A	T
*A. lisae* [MZUSP 7782]	A	G	T	C	T	A	G	T	C	T	T	T	T	A	T
*A. lisae* [MZUSP 7784]	A	G	T	C	T	A	G	T	C	T	T	T	T	A	T
*Anth. hobergi* [MZUSP 7756]	T	A	A	T	C	A	A	T	T	A	G	C	C	T	T
**Species\Site**	**255**	**279**	**300**	**330**	**339**	**348**	**357**	**382**	**385**	**408**	**431**	**472**	**495**	**504**	**528**
*A. inexpectatum* sp. n. [MZUSP 7767]	A	G	G	C	G	A	A	G	C	C	G	C	A	A	T
*A. inexpectatum* sp. n. [MZUSP 7768]	A	G	G	C	G	A	A	G	C	C	G	C	A	A	T
*A. inexpectatum* sp. n. [MZUSP 7769]	A	G	G	C	G	A	A	G	C	C	G	C	A	A	T
*A. inexpectatum* sp. n. [MZUSP 7770]	A	G	G	C	G	A	A	G	C	C	G	C	A	A	T
*A. inexpectatum* sp. n. [MZUSP 7771]	A	G	G	C	G	A	A	G	C	C	G	C	A	A	T
*A. inexpectatum* sp. n. [MZUSP 7772]	A	G	G	C	G	A	A	G	C	C	G	C	A	A	T
*A. inexpectatum* sp. n. [MZUSP 7773]	A	G	G	C	G	A	A	G	C	C	G	C	A	A	T
*A. inexpectatum* sp. n. [MZUSP 7774]	A	G	G	C	G	A	A	G	C	C	G	C	A	A	T
*A. inexpectatum* sp. n. [MZUSP 7777]	A	G	G	C	G	A	A	G	C	C	G	C	A	A	T
*A. inexpectatum* sp. n. [MZUSP 7775]	A	G	G	C	G	A	A	G	C	C	G	C	N	N	N
*A. inexpectatum* sp. n. [MZUSP 7776]	A	G	G	C	G	A	A	G	C	C	G	C	A	A	T
*A. anacolum* [MZUSP 7789]	A	G	C	A	A	A	G	G	T	T	G	C	A	A	T
*A. anacolum* [MZUSP 7778]	A	G	C	A	A	A	G	G	T	T	G	C	A	A	T
*A. anacolum* [MZUSP 7779]	A	G	C	A	G	A	G	G	T	T	G	C	A	A	T
*A. anacolum* [MZUSP 7780]	A	G	A	T	G	A	G	G	T	T	G	C	A	A	T
*A. anacolum* [MZUSP 7781]	A	G	A	T	G	A	G	G	T	T	G	C	A	A	T
*A. carrioni* sp. n. [MZUSP 7787]	T	C	C	A	A	G	G	G	T	T	G	C	G	G	T
*A. carrioni* sp. n. [MZUSP 7788]	T	C	C	A	A	G	G	G	T	T	G	C	G	G	T
*A. carrioni* sp. n. [MZUSP 7785]	T	C	C	A	A	G	G	G	T	T	G	C	G	G	T
*A. carrioni* sp. n. [MZUSP 7786]	T	C	C	A	A	G	G	G	T	T	G	C	G	G	T
*A. lisae* [MZUSP 7783]	G	A	T	G	T	A	T	A	T	T	A	T	T	T	A
*A. lisae* [MZUSP 7782]	G	G	T	A	T	A	T	A	T	T	A	T	T	T	A
*A. lisae* [MZUSP 7784]	G	G	T	A	T	A	T	A	T	T	A	T	T	T	A
*Anth. hobergi* [MZUSP 7756]	T	G	T	A	A	T	T	G	T	T	G	C	A	A	T

**Remarks.** The redescription of *Anindobothrium lisae* added information on the morphology and patterns of distribution of microtriches and cross sections of the ovary for this taxon. Also, we have a better understanding of the distribution, host association and morphological variability of this species. Finally, we provided a molecular diagnosis for *A. lisae* for the first time.

*Anindobothrium lisae* was only known parasitizing *Potamotrygon orbignyi* from the Rio Negro [4]. However, the examination of newly collected material from Venezuela revealed that this species also infects *P. schroederi* from mid-Orinoco. Rio Negro and Orinoco are known to share freshwater fauna [59], including these two species of hosts. Interestingly, *A. lisae* has never been found in *P. schroederi* from the Rio Negro, despite the examination of 31 specimens from there, as well as one from Ilha do Catalão, in the confluence of the Rio Negro and the Rio Solimões. In addition, this cestode has not been observed in *P. orbignyi* after examining 12 specimens from mid-Orinoco (Marques, *unpubl. data*). We found no molecular evidence that can be used to distinguish those two populations of *A. lisae* since the only haplotype from Orinoco included in our study nested between two haplotypes from the Rio Negro (Fig 3A–3C).

The redescription of *A. lisae* provided additional information on morphological variability as compared to what was reported by Marques *et al*. [4]. Comparing the ranges provided in the original description with those reported here revealed a larger spectrum of variation for structures such as bothridial width (322–775 vs. 297–838, respectively), number of poral anterior (5–13 vs. 3–15, respectively), and anti-poral testes (21–38 vs. 15–43 respectively). Also, the redescription provided a better understanding of the bothridial morphology of this species, for which the original account was based on the interpretation and illustration of an immature specimen (see figure 2B in [4]). Furthermore, the examination of additional material showed that this species can have the vitelline follicles partially interrupted by the ovary, which was not observed in the original description.

The early divergence of this lineage of *Anindobothrium* is evident in its morphology and nucleotide sequences. As pointed out above (see Remarks for *A.anacolum*), the morphology of the bothridia in *A. lisae*, which lacks facial loculi and longitudinal septa, is different from all marine species of the genus. As a species, *A. lisae* can be diagnosed by the largest sets of molecular synapomorphies, which comprise a total of 67 sites, and which include two unique insertions in 28S sequences (see Tables 5–9). For diagnostic purposes, however, this is the most conservative approach since there are other positions that could be used in addition to the molecular synapomorphies listed above. For instance, positions 300/T and 357/T for COI (Table 9), 1,032/deletion for 18S (Table 5), 1,042/C and 1,101/C for 28S (Table 8), and 229/deletion and 277/deletion for ITS (Table 7) also may differentiate *A. lisae* from all marine species of the genus.

#### *Anindobothrium inexpectatum* sp. n.

**Fig 11:**
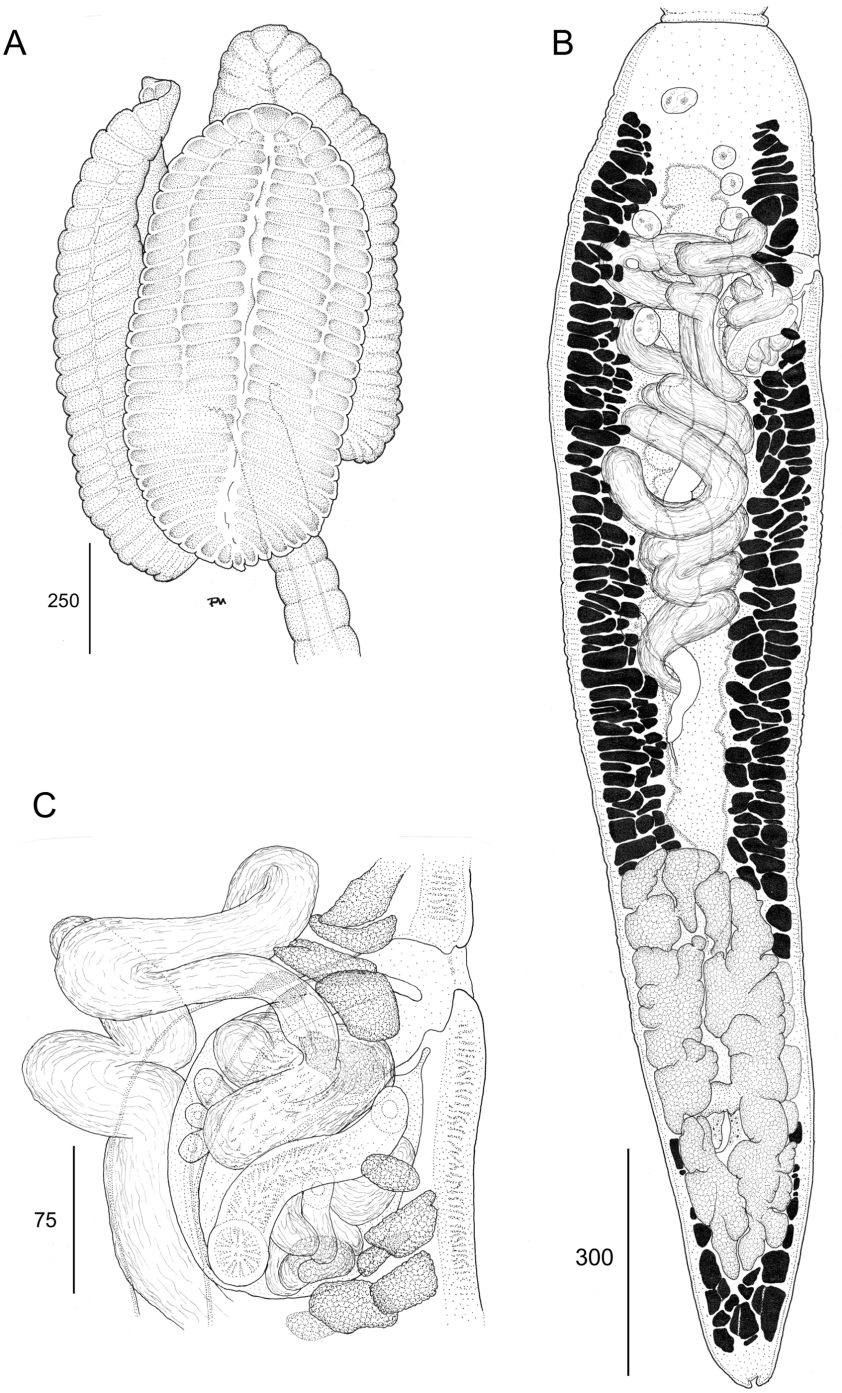
Line drawings of *Anindobothrium inexpectatum* sp. n. ex *Styracura schmardae*. A. scolex (MIUP C-TET-PHY-A1, holotype). B. terminal proglottid (HWML 139137, paratype). C. cirrus sac (MIUP C-TET-PHY-A1, holotype).

**Fig 12:**
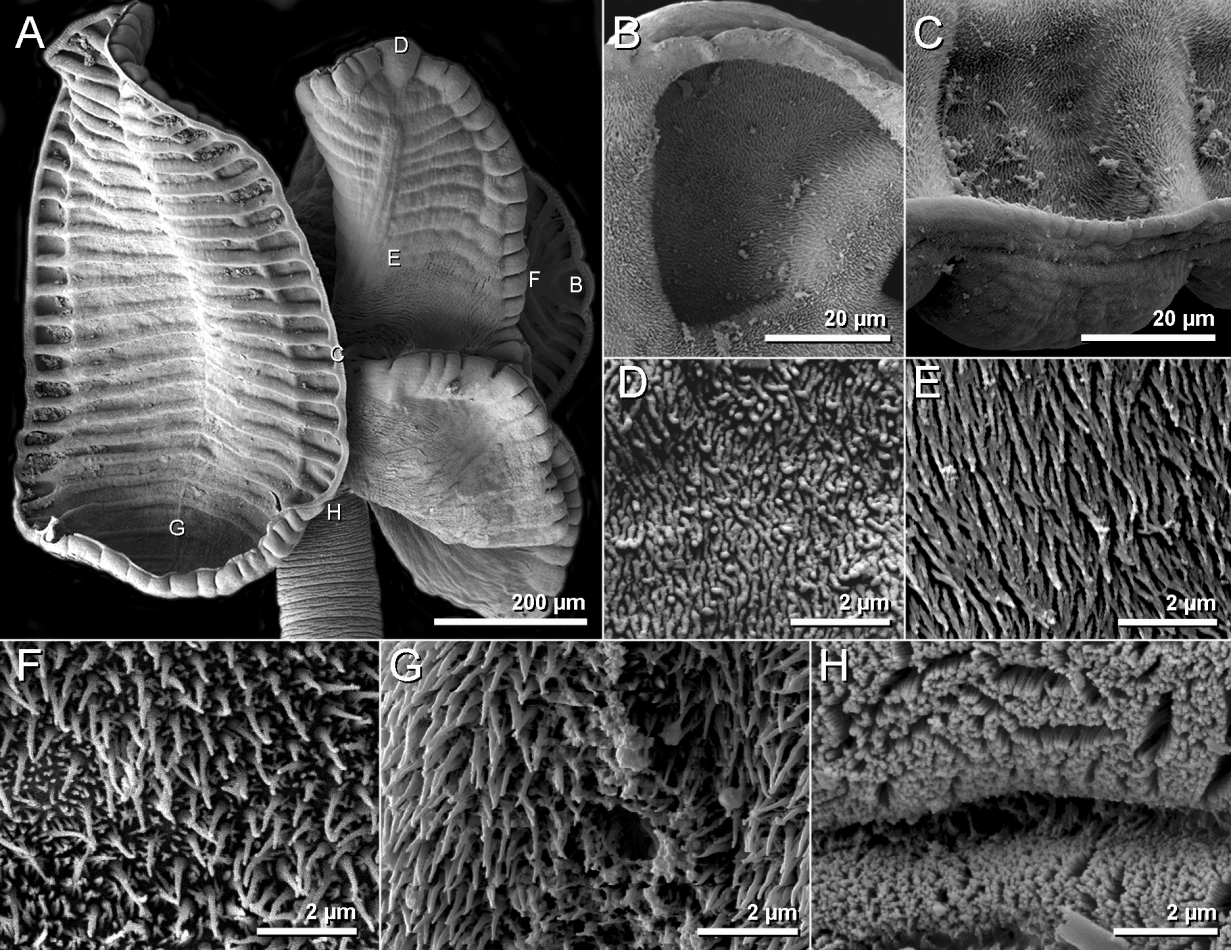
Scanning electron micrographs of *Anindobothrium inexpectatum* sp. n. ex *Styracura schmardae*. A. scolex. B. distal surface of apical sucker. C. distal surface of medial loculi. D. proximal surface of apical sucker. E. proximal bothridial surface near centre of bothridium. F. distal anterior surface of longitunal septum. G. posterior portion of longitudinal septum on distal bothridial surface. H. cephalic peduncle.

urn:lsid:zoobank.org:act:BEDC1D7D-1A11-4DDC-B8BF-2A3D00C837EB

(Figs 6C, 9E and 9F and 11 and 12)

**Type host:** *Styracura schmardae* (Werner) de Carvalho, Loboda & da Silva (Myliobatiformes: Potamotrygonidae).

**Type locality:** Caribbean Sea, Tobacco Caye, Dangriga, Stann Creek, Belize (16°54’ N, 88°03’ W).

**Additional localities:** Caribbean Sea, Head Caye, Punta Gorda, Toledo (16°13’ N, 88°35’ W), north of Southwater Caye, Dangriga, Stann Creek, Belize (16°49’ N, 88°04’ W); Caribbean Sea, Almirante, Bocas del Toro Province, Panama (9°17’ N, 82°20’ W and 9°17’ N, 82°21’ W).

**Site of infection:** Spiral intestine.

**Type material:** **MIUP** C-TET-PHY-A1 [holotype]; **MIUP** C-TET-PHY-A1-P1–P6 [paratypes], **MZUSP** 7767–7776 [molecular vouchers], 7934a–7934j, 7935 [paratypes]; **LRP** 9278–9285 [paratypes]; and **HWML** 139137–139147 [paratypes].

**Etymology:** From the Latin “*inexpectatus*” meaning unexpected, referring to the surprise of finding another species of the genus in the same host in the Caribbean Sea.

**Description.** [Based on 46 mature specimens: 41 whole mounts, three worms observed with SEM, and two serially-sectioned]: Worms acraspedote, apolytic, 4.3–14.1 mm long, composed of 15–39 (39) proglottids (Fig 6C). Scolex with greatest width 458–1,015 (39), composed by four stalked bothridia (Figs 6C, 11A and 12A). Bothridia elliptoid-shaped, 596–1273 (41) long by 222–642 (41) wide, divided by 23–30 (39) transverse septa and one medial longitudinal septum into 47–61 (39) loculi. Medial longitudinal septum extending from posterior margin of apical sucker to posterior margin of bothridium. Apical sucker 40–86 (37) long by 42–83 (36) wide (Fig 12B). Short cephalic peduncle. Proximal surface of apical sucker covered by acicular filitriches (Fig 12D) and medial portion of proximal bothridial surface covered by gladiate spinitriches (Fig 12E). Distal surfaces of bothridia covered by gladiate spinitriches and acicular filitriches (Fig 12C, 12F and 12G). Cephalic peduncle covered by capilliform filitriches (Fig 12H).

Immature proglottids wider than long. Mature proglottids 765–2,058 (39) long by 201–391 (39) wide, 2–6 (39) in number. Some terminal proglottids with sperm-filled vas deferens (Fig 11B). Gravid proglottids not observed. Testes round to oval, 22–57 (38) long by 19–44 (38) wide; 23–44 (37) in number; 3–8 (37) pre-poral, 7–16 (38) post-poral, 12–26 (37) anti-poral (Figs 6C and 9E). Cirrus sac in anterior $\frac{1}{4}$ of proglottid, round, 78–145 (39) long by 84–183 (39) wide, containing eversible coiled cirrus armed with spinitriches (Fig 11B and 11C). Genital atrium present. Genital pores 17–27% (39) of proglottid length from anterior end, irregularly alternating. The vagina runs anterior to the cirrus sac and then turns posteriorly towards the ootype. Ovary near posterior end of proglottid, bilobed in dorso-ventral view, tetra-lobed in cross-section (Figs 11B and 9F), symmetrical, 228–651 (39) long by 97–235 (39) wide at isthmus; antero-ventral lobes converging anteriorly to midline of proglottid, but not fusing; ovarian margins lobulate. Vitelline follicles extending throughout length of proglottid, 11–44 (39) long by 11–26 (39) wide; partial or total interruption by the ovary. Eggs not observed.

**Molecular diagnosis**: *Anindobothrium inexpectatum* sp. n. may be differentiated from other members of the genus by the following set of 17 character states (base/position): **COI**—C/105, G/300, C/330, A/357, C/385, and C/408; **18S**—G/1,032; and **ITS**—T/82, A/83, A/203, A/204, C/229, G/355, C/372, T/417, A/418, and A/548. All character states are unique unambiguous synapomophies (see Fig 3B and 3C; Tables 5, 7 and 9).

**Remarks.** *Anindobothrium inexpectatum* sp. n. was found in specimens of *S. schmardae* collected off the coast of Belize and northeastern coast of Panama in the Caribbean Sea. This species appears to have a disjunctive distribution with respect to *A. anacolum*, despite sharing the same host and close distributional ranges. This somewhat odd host association and distributional pattern would suggest the hypothesis that this new species should be considered a population of *A. anacolum*. We think we have enough evidence to support an alternative interpretation by considering those specimens recovered off the coast of Belize and northeastern coast of Panama in the Caribbean Sea as a new taxon: *Anindobothrium inexpectatum* sp. n. This alternative hypothesis is supported by molecular, morphological data and patterns of endemism in the Caribbean Sea, which will be addressed in the Discussion.

We found 6 unique unambiguous synapomorphies for COI and 11 for the nuclear genes 18S and ITS that can be used to characterize *A. inexpectatum* sp. n. (Fig 3B and 3C; Tables 5, 7 and 9). *Anindobothrium anacolum*, on the other hand, can be diagnosed based on 7 unique unambiguous synapomorphies (see above). Therefore, in total, there are 23 sites that can be used to distinguish the two species (Tables 5–7 and 9). In addition to the sets of molecular synapomorphies that diagnose this species, we also found that specimens of *A. inexpectatum* sp. n. display non negligible amounts of molecular divergence—as inferred by K2P-distances (Table 4)—when compared to haplotypes of *A. anacolum* (*e.g.*, 10.5% for COI, 1.3% for ITS, and 1.0% for Calmodulin). COI divergence is of particular interest, since empirical data for more than 13,000 congeneric pairs among 11 phyla of Metazoa revealed that most pairs (98%) have over 2% sequence divergence in this mitochondrial region [60]. Therefore, given the amount of sequence divergence and that *A. inexpectatum* sp. n. and *A. anacolum* can be diagnosed unambiguously by sets of character states from nucleotide data, we considered *A. inexpectatum* sp. n. as a new species of the genus.

Our hypothesis is also supported by morphological data. As the case for other marine species of the genus, *A. inexpectatum* sp. n. can be differentiated from the freshwater species *A. lisae* by the morphology of the bothridia (see Remarks for *A. anacolum* above). Among marine species, we were unable to find any discrete morphological attribute or morphometric discontinuity that could be used to distinguish *A. inexpectatum* sp. n. from *A. anacolum*. The multivariate statistics analyses of morphometric data showed, however, that *A. inexpectatum* sp. n. not only differs from the marine lineages of the genus but also it is most similar to a lineage of the genus found in the eastern Pacific coast of Panama (Fig 5A and 5B). The PCA analysis (Fig 5A), for instance, showed that the cluster of specimens of *A. inexpectatum* sp. n. overlap more with those from the eastern Pacific congener than to those of *A. anacolum*. The linear discriminant analysis (LDA; Fig 5B) is congruent with the phylogenetic pattern observed in the sense that this analytical tool was able to discriminate *A. inexpectatum* sp. n. from other marine lineages of the genus with an error rate of 3%. Therefore, the absence of a discrete morphological attribute or any morphometric discontinuity should not pose any problem to identify *A. inexpectatum* sp. n. on the bases of morphological data, since discriminant function analysis would serve this purpose.

#### *Anindobothrium carrioni* sp. n.

**Fig 13:**
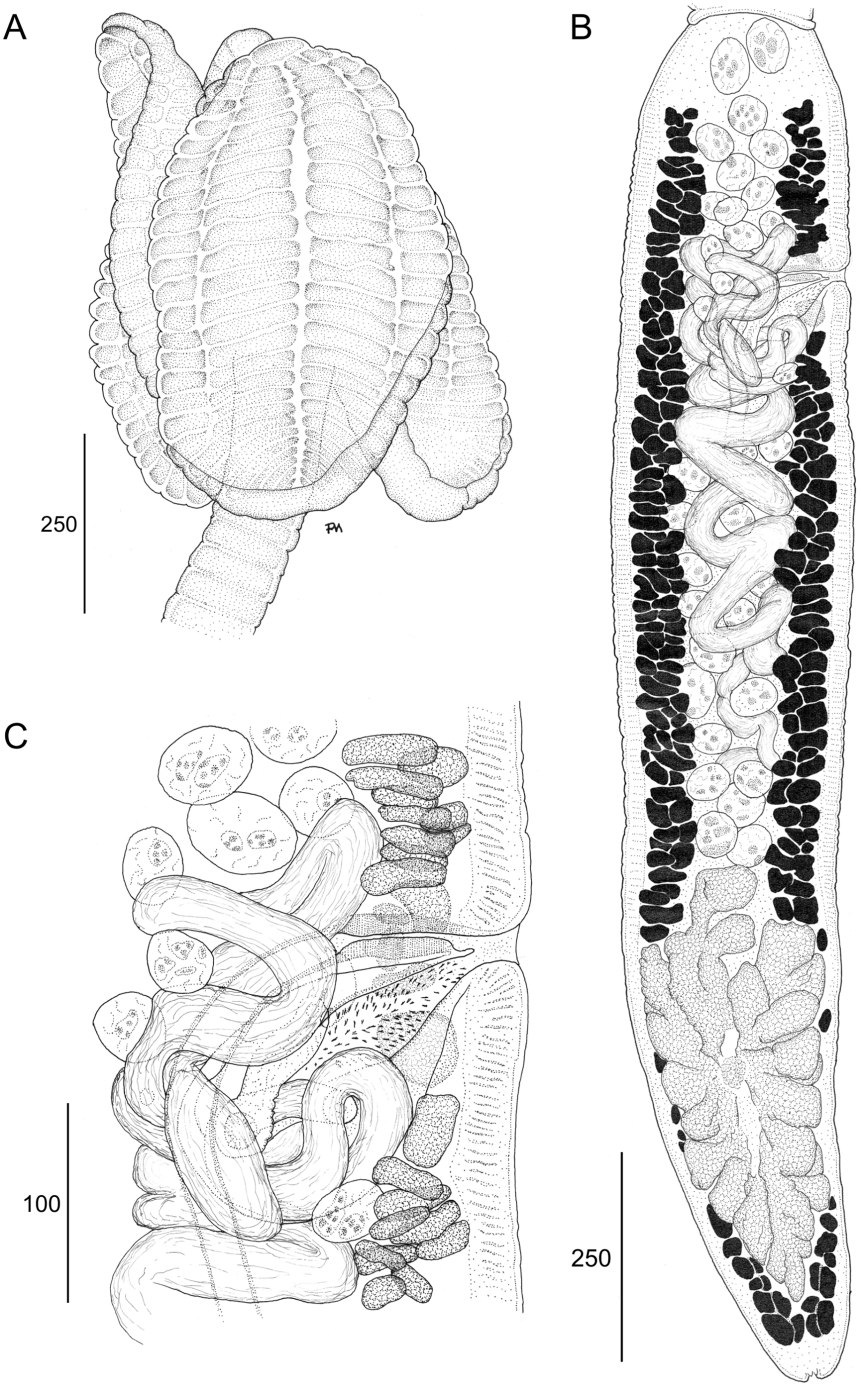
Line drawings of *Anindobothrium carrioni* sp. n. ex *Styracura pacifica*. A. scolex (MIUP C-TET-PHY-A2, holotype). B. terminal proglottid (LRP 9286, paratype). C. cirrus sac (LRP 9286, paratype).

**Fig 14:**
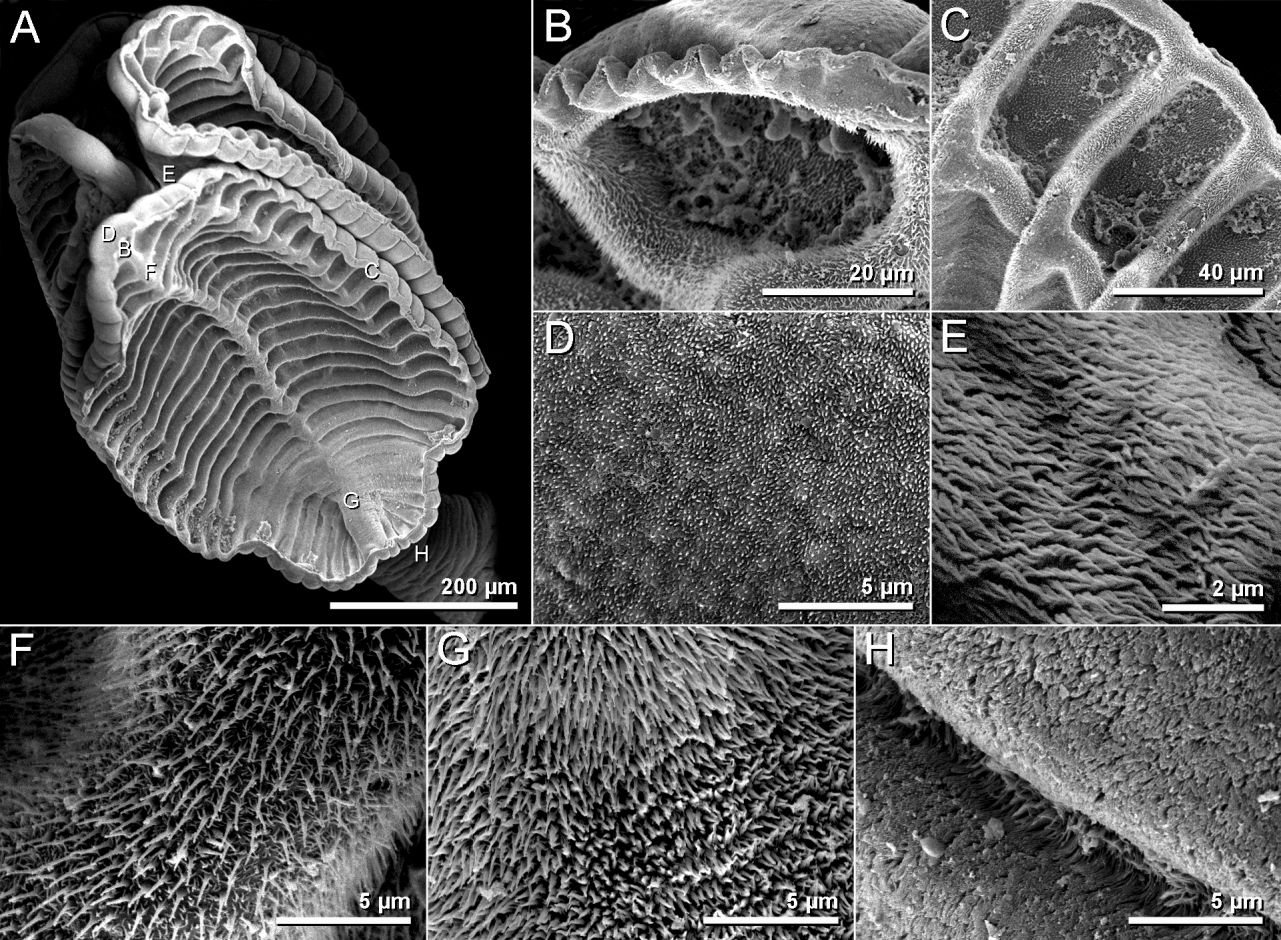
Scanning electron micrographs of *Anindobothrium carrioni* sp. n. ex *Styracura pacifica*. A. scolex. B. distal surface of apical sucker. C. distal surface of medial loculi. D. proximal surface of apical sucker. E. proximal bothridial surface near centre of bothridium. F. distal anterior surface of longitunal septum. G. posterior portion of longitudinal septum on distal bothridial surface. H. cephalic peduncle.

urn:lsid:zoobank.org:act:0A955A70-E88D-4EFC-98AB-13851FB6EB4B

(Figs 6D, 9G and 9H and 13 and 14)

**Type host:** *Styracura pacifica* (Beebe & Tee-Van) de Carvalho, Loboda & da Silva (Myliobatiformes: Potamotrygonidae).

**Type locality:** Pacific Ocean, Playa Caleta, Montijo, Veraguas, Panama (7°29’ N, 81°13’ W).

**Site of infection:** Spiral intestine.

**Type material:** **MIUP** C-TET-PHY-A2 [holotype], **MIUP** C-TET-PHY-A2-P1–P4 [paratypes]; **MZUSP** 7785–7788 [molecular vouchers], 7936a–7936d [paratypes]; **LRP** 9286–9290 [paratypes], and **HWML** 139148–139155 [paratypes].

**Etymology:** This species is named in honor of Señor Agustín Carrión, a gifted and witty fisherman who guided us to find *Styracura pacifica* in the Gulf of Montijo during our collecting trip to the eastern Pacific coast of Panama.

**Description.** (Based on 36 mature specimens: 32 whole mounts, two worms observed with SEM, and two serially-sectioned): Worms acraspedote, apolytic, 4.8–13.9 mm (32) long, composed of 20–33 (32) proglottids (Fig 6D). Scolex with greatest width 368–763 (32) composed by four stalked bothridia (Figs 6D, 13A and 14A). Bothridia elliptoid-shaped, 531–1,000 (32) long by 162–375 (32) wide, divided by 21–26 (28) transverse septa and one medial longitudinal septum into 43–53 (28) loculi. Medial longitudinal septum extending from posterior margin of apical sucker to posterior margin of bothridium. Apical sucker 38–64 (23) long by 52–86 (23) wide (Fig 14B). Short cephalic peduncle. Proximal surface of apical sucker covered by acicular filitriches (Fig 14D) and medial portion of proximal bothridial surface covered by gladiate spinitriches (Fig 14E). Distal surfaces of bothridia covered by gladiate spinitriches and acicular filitriches (Fig 14C, 14F and 14G). Cephalic peduncle covered by capilliform filitriches (Fig 14H).

Immature proglottids wider than long. Mature proglottids 648–1,752 (30) long by 164–353 (30) wide, 3–6 (32) in number. Some terminal proglottids with sperm-filled vas deferens (Fig 13B). Gravid proglottids not observed. Testes round to oval, 32–65 (27) long by 26–45 (27) wide; 21–31 (29) in number; 2–5 pre-poral, 6–11 post-poral, 11–19 (29) anti-poral (Figs 6D and 9G). Cirrus sac in anterior $\frac{1}{4}$ of proglottid, round, 58–135 (26) long by 72–134 (26) wide, containing eversible coiled cirrus armed with spinitriches (Fig 13B and 13C). Genital atrium present. Genital pores 15–29% (29) of proglottid length from anterior end, irregularly alternating. The vagina runs anterior to the cirrus sac and then turns posteriorly towards the ootype. Ovary near posterior end of proglottid, bilobed in dorso-ventral view, tetra-lobed in cross-section (Figs 13B and 9H), symmetrical, 230–570 (28) long by 112–199 (27) wide at isthmus; anteroventral lobes converging anteriorly to midline of proglottid, but not fusing; ovarian margins lobulate. Vitelline follicles extending length of proglottid, 15–56 (27) long by 12–29 (27) wide; partial or total interruption by the ovary. Eggs not observed.

**Molecular diagnosis**: *Anindobothrium carrioni* sp. n. may be differentiated from other members of the genus by the following set of 25 character states (base/position): **COI**—T/141, A/168, C/219, C/250, C/279, G/348, C/483, G/495, and G/504; **18S**—A/1,042; **28S**—A/591; **Calmodulin**—A/122, T/307, and T/312; and **ITS**—T/213, G/277, A/281, T/339, T/355, T/461, C/464, C/552, C/653, T/722, and A/774. All character states are unique unambiguous synapomophies (see Fig 3B and 3C; Tables 5–9).

**Remarks.** *Anindobothrium carrioni* sp. n. is the only species of the genus known from the eastern Pacific Ocean. This species is sister to the clade comprised by the remaining marine species of the genus reported from the Caribbean Sea, *A. anacolum* and *A. inexpectatum* sp. n. This phylogenetic pattern mirrors many examples in the literature in which the uplift of the Isthmus of Panama is credited to have generated sister clades in many groups of Metazoa (see references in [61, 62]).

The molecular evidence for the recognition of *Anindobothrium carrioni* sp. n. as a segregated lineage within the genus is overwhelming. Members of this species have a set of 25 unique unambiguous synapomorphies—9 from COI and 16 from nuclear regions (Fig 3B and 3C; Tables 5, 6, 7 and 9). Sequences of *Anindobothrium carrioni* sp. n. are the most divergent ones among marine species of the genus. In average, sequences of COI, Calmodulin and ITS—the most variable regions included in this study—differed from *A. anacolum* and *A. inexpectatum* from Caribbean in 13.8%, 2.7% and 2.7%, respectively (Table 4). This constitutes strong molecular support to recognize *Anindobothrium carrioni* sp. n. as a new species.

Despite the molecular divergence and sets of diagnostic character states, all marine species of the genus have a very similar morphology, especially with regards to the bothridial architecture that share the presence of longitudinal septa and facial loculi. The bothridial morphology can be used to distinguish the marine lineages from the only species known to be restricted to potamotrygonids in freshwater systems of South America, *A. lisae*, as discussed earlier. However, there is no discrete morphological character or discontinuous morphometric variable that could be used to distinguish *A. carrioni* sp. n. from *A. anacolum* and *A. inexpectatum* sp. n. But, based on the PCA analysis *A. carrioni* sp. n. seems to be phenetically closer to *A. inexpectatum* sp. n., since the area circumscribed by the 95% confidence interval around the centroids for each species overlap to a great extent (Fig 5A). Despite the overlap observed in the PCA plot, all marine species are discriminated in the LDA (Fig 5B). Therefore, as for *A. inexpectatum* sp. n., *A. carrioni* sp. n. could only be recognized morphologically by discriminant function analysis.

## Discussion

### Phylogeny of the Rhinebothriidea and the position of *Anindobothrium*

The order Rhinebothriidea was erected by Healy *et al*. [2] as the result of a phylogenetic analysis based on molecular data for a selected group of the polyphyletic Phyllobothriidae, then an family of Tetraphyllidea. Healy *et al*. [2] circumscribed the taxonomic representation of their study upon Euzet’s [63, 64] concept of the Rhinebothriinae Euzet, 1953, which was proposed to accommodate phyllobothriids that lacked a myzorhynchus in adult forms and which had subdivided and unarmed bothridia. Healy *et al*. [2] found molecular support for a number of phyllobothiids—especially members of Echeneibothriinae and Rhinebothriinae—that were also characterized by possessing stalked bothridia. Accordingly, the original concept of the order Rhinebothriidea included members of *Anthocephalum* Linton, 1890, *Echeneibothrium* van Beneden, 1850, *Rhabdotobothrium* Euzet, 1953, *Rhinebothroides* Mayes, Brooks & Thorson, 1981, *Rhinebothrium* Linton, 1890, *Rhodobothrium* Linton, 1889, *Scalithrium* Ball, Neifar & Euzet, 2003, *Spongiobothrium* Linton, 1889, and the undescribed “New genera 1–4” (sensu Healy *et al*. [2]). In addition, based on the putative morphological synapomorphy of the order, Healy *et al*. (2009) suggested that some of the genera in the subfamily Phyllobothriinae such as *Anthobothrium* van Beneden, 1850 and *Carpobothrium* Shipley & Hornell, 1906 could ultimately be found to be members of the order. They also included as potential members of the new order *Anindobothrium* and *Pararhinebothroides* Zamparo, Brooks & Barriga, 1999 as they were also described as possessing stalked bothridia.

The internal relationships and composition of the Rhinebothriidea was revisited recently in two studies. Ruhnke *et al*. [8] expanded the taxon sampling of Healy *et al*. [2] by adding putative members of the order, in addition to most species of *Anthocephalum* recognized to date. Based on their phylogenetic hypothesis, they recognized four families within the Rhinebothriidea: Rhinebothriidae, Echeneibothriidae, Anthocephaliidae and Escherbothriidae, from which the latter two families were newly erected. Marques and Caira [10] corroborated Ruhnke *et al*.’s [8] suspicion that *Pararhinebothroides* not only was a member of the Anthocephaliidae, but in fact a member of *Anthocephalum*. In both studies, however, no members of *Anindobothrium* were included.

**Fig 15:**
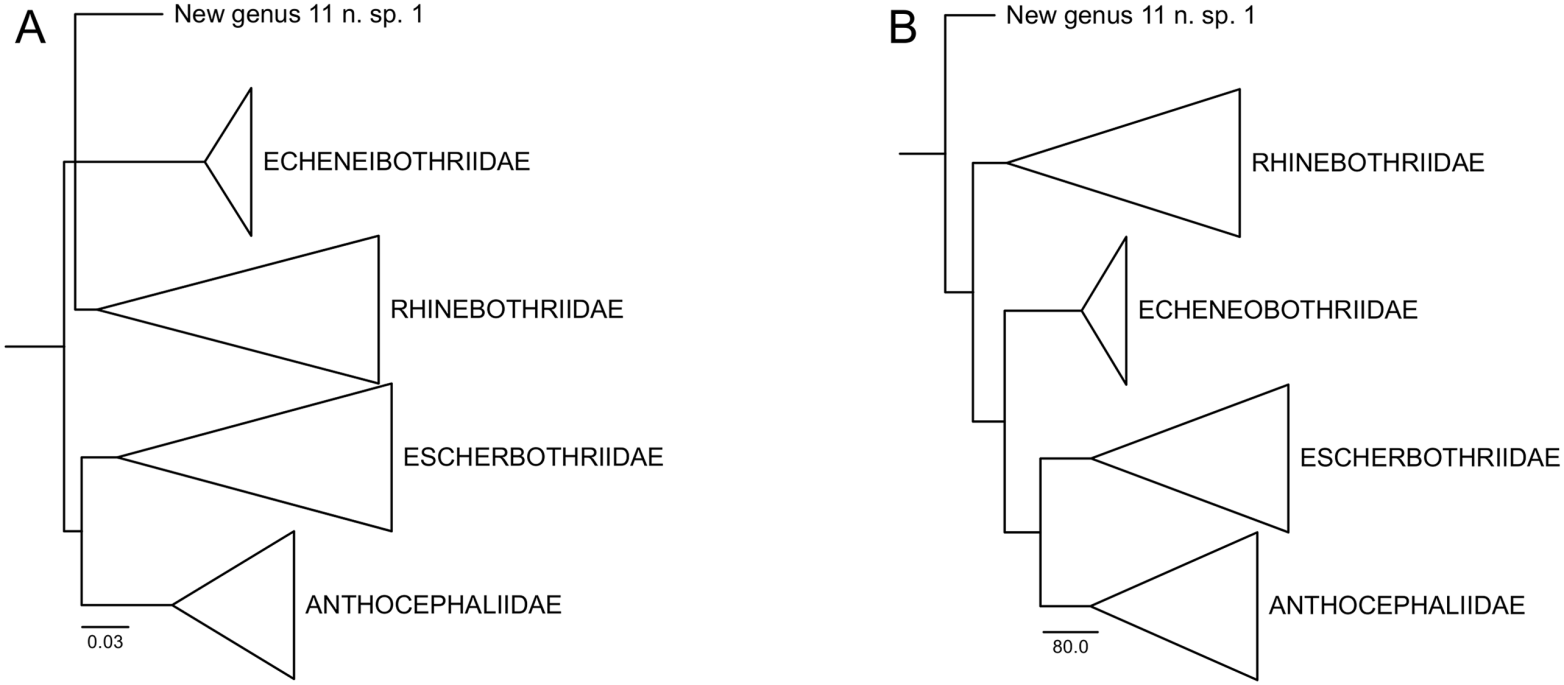
Previous phylogenetic hypotheses for members of the Rhinebothriidea based on LSU and SSU nucleotide data. A. Modified from Ruhnke *et al*. [8]. B. Modified from Marques and Caira [10].

Our phylogenetic analysis provided unambiguous evidence that *Anindobothrium* is a member of the Rhinebothriidea (Fig 1A and 1B). However, the phylogenetic position of this genus seems to be unstable, as are most internal nodes within the order. A comparison between the phylogenetic hypotheses proposed by Ruhnke *et al*. [8] and Marques and Caira [10] is an example of this instability (Fig 15A and 15B). Although both studies suggested that the Anthocephaliidae and the Escherbothriidae are sister taxa, they proposed different phylogenetic arrangements for the remaining taxa. Comparing the present results to previous studies, we observed that each study provided different sets of sister-group relationships for the families (see Figs 1 and 15). All studies had different taxon sampling schemes and some phylogenies were based on different optimality criteria. However, we believe that most of the discrepancies among these studies are related to nodes that have relatively low support due to the apparent limited resolution power of 18S and 28S rDNA. Hence, the inclusion of additional markers in future studies could provide a more stable resolution for the inter-relationships among families of the Rhinebothriidea.

Although sister-group relationships within the Rhinebothriidea are unstable, all families—including the Anindobothriidae—are well supported by the data regardless of optimality criteria (Fig 1A and 1B). This support stems from the large sets of molecular synapomorphies for each clade, albeit not all of them are unique and unambiguous (Fig 2). In addition, all members of this monotypic family possess genital pores at the anterior $\frac{1}{4}$ of the proglottids and have testes anterior and posterior to the cirrus sac. Therefore, Anindobothriidae can be circumscribed by both molecular and morphological characters.

### Species diversity within *Anindobothrium*

The diversification of *Anindobothrium* can be correlated to all major paleogeographic and biogeographical events that shaped the biotas of the Netropical freshwater systems, the tropical eastern Pacific and the tropical western Atlantic Oceans. These events comprise the colonization of the fluvial systems of South America by marine lineages, including an ancestor potamotrygonid lineage, during the Paleogene Period—between the early Miocene and mid-Eocene (*i.e.*, 22.5–46 Mya) [11, 12, 14, 15]—and the isolation of the transisthmian marine fauna during the Late Pliocene—3.2–2.7 Mya [61, 62, 65].

**Fig 16:**
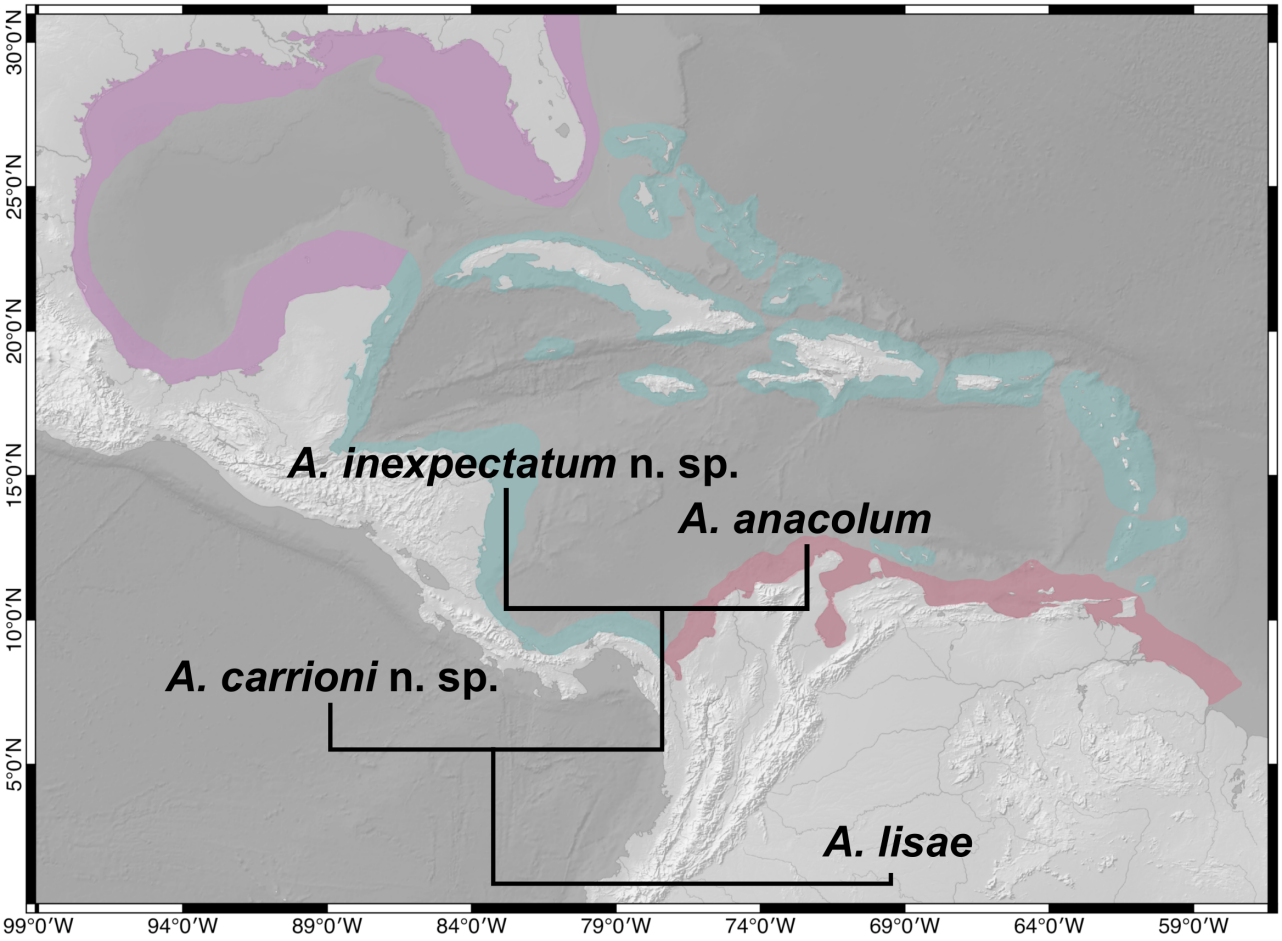
Diversification of *Anindobothrium*. Colored coast lines of the Great Caribbean represent three major provinces based on patterns of endemism as suggested by Robertson & Cramer [66]: Northern Province—the Gulf of Mexico and southeastern USA [pink]; Central Province—the West Indies, Bermuda and Central America [blue]; and Southern Province—Northern South America (red). [Map raster source by Natural Earth in QGIS Geographic Information System/Open Source Geospatial Foundation].

The phylogeny of *Anindobothrium* is congruent with the history of colonization of freshwater stingrays. The current hypothesis of the origin of these freshwater stingrays suggests that the ancestor of this lineage colonized the rivers of South America after the marine incursions in the northern region of that continent during the Miocene [11, 14, 15, 17]. As postulated initially, freshwater potamotrygonids formed a clade sister to amphi-American species of *Himantura* Müller & Henle, which were recently transferred to a new genus, *Styracura*, within the Potamotrygonidae by de Carvalho *et al*. [18]. According to the phylogeny of the host and its biogeographical implications, the divergence of the freshwater lineage took place prior to the diversification of *Styracura* spp., possibly as a result of the closure of the Isthmus of Panama (see below). The phylogeny of *Anindobothrium* mirrors the phylogeny of the host, suggesting an event of codivergence (sensu Page & Charleston [19]) at the split between marine and freshwater lineages of *Anindobothrium* (Fig 16).

It is puzzling, however, that so far, no other freshwater species of *Anindobothrium* have been found and *A. lisae* is the only representative of the genus in freshwater potamotrygonids. Other lineages of cestodes, which had the same historical fate as *Anindobothrium*, diversified after the colonization of the river systems of South America. There are 35 species of freshwater stingrays distributed throughout all major river basins of South America [67, 68]. These hosts house at least 6 valid species of *Acanthobothrium*, 8 of *Potamotrygonocestus* Brooks & Thorson, 1976, 5 of *Rhinebothroides*, 7 of *Rhinebothrium*, and 2 of *Paroncomegas* Campbell, Marques & Ivanov, 1999. In addition, for all of these genera there are many species waiting formal description (Marques, *unpub. data*) as a result of an extensive collecting effort in the past years that led to the examination of ∼1,300 host specimens of freshwater potamotrygonids. Yet, *A. lisae* is the only species in the genus, which compared to many other cestodes found in freshwater potamotrygonids has a very restricted distributional range: upper-middle Orinoco and Rio Negro—two river basins that are known to share freshwater lineages since they are connected by the Casiquiare river [59]. The reasons behind the lack of diversification of *Anindobothrium* in freshwater systems, as compared to other lineages of cestodes in the same habitat or its marine congeners, still need to be elucidated. One possibility might be the life cycle requirements for members of this genus, which could differ from that of other genera found in freshwater potamotrygonids. However, very little is known about the life cycles of these cestodes.

The paleogeographic history of the tropical eastern Pacific and tropical western Atlantic Oceans seems to have imposed the pattern of diversification we observed in marine lineages of *Anindobothrium* and their hosts. After the marine incursions into South America in the Miocene—credited to have allowed the colonization of South American rivers by many marine lineages (see Lovejoy *et al*. [17] and references therein)—the region that now comprises the tropical eastern Pacific and tropical western Atlantic Oceans underwent drastic changes due to the uptlift of the Isthmus of Panama that now separates these bodies of water. The seaway that connected these two regions started narrowing and shallowing by the middle Miocene (13-12 Mya [65, 69, 70]) causing the interruption of gene flow between shallow marine animal populations in the Late Pliocene ∼3.2 Mya [62]. This major paleogeographic event is known to have driven global oceanic reorganization and major biotic change on land and at sea [62], as it not only allowed the exchange of fauna between North and South America (see review by Leigh *et al*. [65]) but also imposed independent evolutionary trajectories of marine organisms now segregated to two oceans affected by changes in current patterns, salinity, temperature, and primary productivity [61]. As a result, the uplift of the Isthmus of Panama affected the taxonomic composition of its adjacent waters [65].

The pattern of diversification of marine lineages of *Anindobothrium* and their hosts is congruent with this paleogeographical event. Species of *Styracura* have transisthmian distribution in which *S. pacifica* is known for the tropical eastern Pacific Ocean and *S. schmardae* is found along the Caribbean coast of Central America and northern coast of South America. The distribution of *Styracura* spp. resembles the pattern documented for many other species pairs of marine lineages with transisthmian distribution (see [61, 62] and references therein) for which as early as 1908 Jordan [71] coined the term “geminate species”. The split between *Anindobothrium carrioni* and the clade formed by the Caribbean species of this genus mirrors the divergence between their host (*S. pacifica* and *S. schmardae*) suggesting codivergence between host and parasite lineages (Fig 16). In addition, the molecular divergence between transisthmian lineages of *Anindobothrium* is within the range of what is documented for many other groups of Metazoa for which species pairs are believed to have originated by the uplift of the Isthmus of Panama [61].

Lessios [61] compared the K2P nucleotide sequence distances for 38 genomic regions involving 115 pairs of geminate clades of echinoids (9), crustaceans (38), fishes (42), and molluscs (26). He concluded, among other things, that 34 clades had diverged at the time of the Isthmus of Panama completion, when gene flow was interrupted between the isolated areas (*i.e.*, 3.2–2.7 Mya [61, 62, 65]). For the most prevalent genomic region used in his study, Lessios [61] suggested that ranges of K2P distances for COI of 8.7–13.5% for echinoids, 4.1–8.7% for crustaceans, 3.2–5.5% for fishes, and 7.4–9.2% for molluscs were an indication that those clades diverged at the time of the Isthmus completion. Although we acknowledge that genetic divergence is not produced exclusively by vicariant events, since other factors can affect it as well (*e.g.*, different effective population sizes, differential mutation rates, or distinct modes and intensities of selection), it is interesting to notice that the sister clade of marine lineages of *Anindobothrium* (*i.e.*, *A. carrioni* + *A. anacolum*/*A. inexpectatum*) presented a K2P distance of 4.9–16.2%, averaging 13.8% for COI. These values may suggest that these lineages were segregated prior to the final stages of the Isthmus completion, as thought to be the case for most of the geminate pairs included in Lessios’s [61] study.

The final event of diversification in marine lineages of *Anindobothrium* involved the split between *A. anacolum* and *A. inexpectatum* hosted by *S. schmardae* from the Caribbean Sea. This putative event of associate-lineage duplication (sensu Page & Charleston [19]) was surprising, albeit it is congruent with the patterns of endemism reported for the Caribbean [66, 72].

Although the tropical eastern Pacific Ocean and the Caribbean Sea are sister areas with a common faunal heritage, the uplift of the Isthmus of Panama had great ecological impact on both now separated bodies of water. This is most evident in the Caribbean, which, compared to the ancestral area, became a mainly oligotrophic and rich in coral reefs albeit in some parts it retained eutrophic environments prevalent before the closure of the Isthmus of Panama [61, 65, 66]. The heterogeneity of the Caribbean has driven areas of endemism that have been recognized as early as 1950’s [73] (see also Robertson & Cramer [66] and references therein). Robertson & Cramer [66] is the most recent and comprehensive study on patterns of distribution of shorefishes of the Caribbean. They analyzed ∼800,000 species site records which included 1,559 species of elasmobranchs and bony fishes from the Caribbean reported for the upper 100 m of the water column of continental and insular shelves. They found evidence to recognize three biogeographical provinces: the Northern Province—the Gulf of Mexico and southeastern USA; the Central Province—the West Indies, Bermuda and Central America; and the Southern Province—Northern South America (see Fig 16). Two of these provinces are occupied by different species of *Anindobothrium*.

The distribution of the sister species *A. anacolum* and *A. inexpectatum* are congruent with the biogeographical provinces recognized by Robertson & Cramer [66] (Fig 16). *Anindobothrium anacolum* seems to be restricted to the Southern Province, which included the entire continental shelf of northern South America from Colombia to northern Guyana [66]. This biogeographical province is quite different from the Central Province from which *A. inexpectatum* is reported. The continental shelf of northern South America is characterized by having a tropical and eutrophic environment due to high nutrient inputs from coastal wind-driven upwelling systems and outflows from large rivers that drain from South America. This is quite different from the Central Province characterized by eutrophic environments in which primary production occurs on the sea floor [65]. To date, only a single species of *Styracura* is known from the Caribbean Sea and, as some species included in Robertson & Cramer’s study, *S. schmardae* seems to be distributed throughout the Central and Southern Provinces. However, the pattern of host association for the Caribbean species of *Anindobothrium* calls for a close examination of the population structure of *S. schmardae*, since there is a possibility that this host group is, in fact, a species complex.

The apparent recent diversification of marine lineages of *Anindobothrium* may respond to the cohesive morphological attributes of these species. To the best of our knowledge there is no discrete morphological attribute that could be used to identify any of the three species. The same applies for the recognition of morphometric discontinuities, which have been the prevailing criterion for species recognition in related groups of cestodes found in elasmobranchs and other vertebrates. The only morphological signal we were able to recover to diagnose these species was using discriminant function analysis (Fig 5). Linear discriminant functions have already been used successfully by others to recognize parasite lineages [74, 75] and other groups of Metazoa (*e.g.*, [76, 77]). Our study suggests that the inclusion of discriminant function analysis into the toolbox of cestode systematics might be fruitful, especially in the absence of molecular data, which for *Anindobothrium* provided discrete nucleotide data to diagnose all the species we now recognize for the genus. We are not advocating that species should be erected on the basis of molecular or discriminant analyses alone—although some have taken this path [78, 79]. The approach adopted in the present study should be an example of integrative taxonomy (sensu Padial *et al*. [80, 81]), a concept based on integration by congruence of evidence generated from the analysis and evaluation of data from different sources (*e.g.*, molecular, morphological, biogeographical, among others). This approach, as was undertaken in the present study, has the potential to support robust hypotheses for species.

### Integrative taxonomy and cryptic species in cestodes

The morphological homogeneity of these three marine species of *Anindobothrium* and their distribution in two species of batoid fishes draws attention to a component on the diversity of cestodes that might have been neglected in the past, that is cryptic species—those assumed to be only recognized by the examination of molecular data. Reports of cryptic species have been published for cestodes before (see reviews in [82, 83]), but to the best of our knowledge, no accounts of cryptic species exists for cestodes infecting elasmobranchs. Even so, compared to other groups of Metazoa, there is a small number of publications addressing cryptic speciation in cestodes. Pérez-Ponce de León & Nadler [82] reported 15 studies referring to cryptic species for this group from 1999 to 2009. Recently, this list was revised by Pérez-Ponce de León & Poulin [83] who found 14 studies from 1978 to June 2016 for cryptic species in cestodes.

Independent of the status of *A. inexpectatum* as a truly cryptic species or a pseudocryptic one—those not initially recognized as phenotypically distinct due inadequate analysis of morphological data [84, 85]—, this species would not have been recognized by traditional approaches applied by taxonomists in the group. The taxonomy of cestodes, in particular those lineages parasitic in elasmobranchs, has historically been based on morphological discontinuities and on the assumption that cestode fauna of a species of elasmobranch does not vary substantially across its distribution [86]. Since cryptic lineages have been shown to be common throughout the metazoan taxa (see [87–91], and references therein), we should expected to find them also among cestode species.

Many studies have found cryptic species by evaluating the molecular diversity of wide spread taxa composed by populations that are morphologically similar—if not indistinguishable (*e.g.*, [79, 92–98]). These studies illustrate the importance of recognize cryptic speciation as part of the process that produces biodiversity since the failure to detect cryptic species can result in underestimation of biodiversity [91]. The importance of recognizing this component of our biota relies on the fact that most questions in evolutionary biology (*e.g.*, speciation), ecology (*e.g.*, ecosystem development), conservation biology (*e.g.*, conservation priorities) or biogeography (*e.g.*, diversification processes) depend, to a great extent, on the accurate recognition of the lineages that comprise that biodiversity [80]. For host-parasite systems, the circumscription of host and parasite lineages shape our understanding of host specificity, influence our efforts to control parasitic diseases and may determine our ability to provide robust hypotheses for historical association events [83, 99].

As pointed out by Goldstein & DeSalle [100], the documentation of cryptic species demands creative approaches. The inclusion of scanning electron microscopy is a common practice in the taxonomy of elasmobranch cestodes to document tegumental structures (*i.e.*, microtriches patterns) and it has been useful in taxonomic decisions (see Caira [86] and references therein). Also, the concern for selecting characters not subject to fixation artifacts and the search for those thought to display interspecific variation are credited to have improved the taxonomy of cestodes over the years [86]. Molecular data have contributed to the taxonomy of many groups of cestodes, however timidly so. A non-exhaustive survey of cestode species described in the past 10 years revealed that more that 300 species were described during this period (source: Global Cestode Database [101] and ISI Web of Knowledge; December 2016), from which 47 species were described in studies that included molecular data (*e.g.*, [7, 8, 102–124]). In addition to the list provided by Pérez-Ponce de León [83] accounting for studies revealing cryptic species in cestodes we found 8 more publications [125–132] (Source: ISI Web of Knowledge, March 2017). Among all theses studies, only one described a species after recognizing it as cryptic, which turned out also to be distinct morphologically [131] (see also Nakao *et al*. [128]). Marques *et al*. [133], for instance, explicitly recognized that their study provided molecular data to circumscribe two different species of *Didymobothrium* Nybelin, 1922, but refrained to make any nomenclatural changes as they were unable to provide morphological diagnoses.

Molecular data has greatly accelerated the identification of cryptic species (see Padial *et al*. [81], and references therein), but the sole identification of cryptic species will not solve the taxonomic challenges we face today. Formally described species are not only the basis for biological classification but also the only means by which we can effectively communicate and link information to existing knowledge [134]. Be that as it may, molecular data are rarely included in formal descriptions [134, 135] and there is still the prevailing notion that a morphological diagnosis is required to describe species, although no current codes for biological nomenclature specify the class of characters upon which descriptions ought to be based [136]. Thus, the trend observed in cestode systematics mirrors the practice observed in other groups.

Especially in cases of cryptic species, we should acknowledge that molecular data could be valuable as diagnostic characters in the absence of morphological discontinuities and there is no epistemological justification to exclude them. The goal of modern taxonomy is to provide sets of characters upon which we can erect species hypotheses. Systematists should be under no obligation to stick to any class of data (*i.e.*, phenotypic and/or genotypic) in their taxonomic practice. Rather, we should embrace the virtues of “integrative taxonomy”, which seems to be the most profitable path not only to incorporate all information available at the time into species description but also to provide a more robust empirical foundation for systematics [100]. Systematics, as a science, should proceed via free exploration of ideas, and not by peer pressure to give preference to any class of characters as the primary data source for species descriptions nor by restricting the methods available to investigators [137]. We predict that as we closely look at cestode fauna across biogeographical areas and apply a variety of tools available for species discovery, we will encounter closely related species morphologically very similar but yet displaying discrete sets of molecular characters, which may also be congruent with other attributes (*e.g.*, host association, paleogeographical history, among others). Our only concern should be to provide testable hypotheses about the structure of biodiversity [138], which can be corroborated or refuted in light of new empirical data.

## Supporting information

S1 Text Hosts examined in the present study. (TEX)

S1 Fig Phylogeny of the Rhinebothriidea based on parsimony analysis of LSU and SSU nucleotide data. (TIF)

S2 Fig Phylogeny of the Rhinebothriidea based on maximum likelihood analysis of LSU and SSU nucleotide data. (TIF)

S1 Table Correlation matrix (Pearson) between morphometric variables considered for marine lineages of *Anindobothrium*. (TEX)

S2 Table Loadings values for each variable in Principal Component Analysis (PCA). (TEX)

S3 Table Loadings values for each variable in Linear Discriminant Analysis (LDA). (TEX)

## References

[1] Reyda FB. Intestinal helminths of freshwater stingrays in southeastern Peru, and a new genus and two new species of cestode. J Parasitol. 2008;94: 684–699. 10.1645/GE-1230.1 18605776

[2] Healy CJ, Caira JN, Jensen K, Webster BL, Littlewood DTJ. Proposal for a new tapeworm order, Rhinebothriidea. Int J Parasitol. 2009;39: 497–511. 10.1016/j.ijpara.2008.09.002 18929566

[3] Ruhnke TR. Tapeworms of Elasmobranchs (Part III). A monograph on the Phyllobothriidae (Platyhelminthes: Cestoda). Bulletin of the University of Nebraska State Museum; 2011;25: i–xii, 1–208.

[4] Marques FPL, Brooks DR, Lasso CA. *Anindobothrium* n. gen. (Eucestoda: Tetraphyllidea) inhabiting marine and freshwater potamotrygonid stingrays. J Parasitol. 2001;87: 666–672. 10.1645/0022-3395(2001)087[0666:ANGETI]2.0.CO;2 11426733

[5] Vermes Braun M., Abtheilung I. b. Cestodes In: Bronn H.H. Klassen und Ordnungen des ThierReichs. Leipzig: C. F. Winter’sche Verlagshandlung; 1894–1900 p. 927–1731 + XXXV–LIX.

[6] Carus JV. Vermes In: Carus JV. Handbuch der Zoologie. Zweiter Band. Raderthiere, Würmer, Echinodermen,Coelenteraten und Protozoen. Leipzig: Wilhelm Engelmann; 1863 p. 422–484.

[7] Caira JN, Jensen K, Waeschenbach A, Olson PD, Littlewood DTJ. Orders out of chaos—molecular phylogenetics reveals the complexity of shark and stingray tapeworm relationships. Int J Parasitol. 2014;44: 55–73. 10.1016/j.ijpara.2013.10.004 24275646 PMC4834996

[8] Ruhnke TR, Caira JN, Cox A. The cestode order Rhinebothriidea no longer family-less: A molecular phylogenetic investigation with erection of two new families and description of eight new species of *Anthocephalum*. Zootaxa. 2015;3904: 51–81. 10.11646/zootaxa.3904.1.3 25660771

[9] Ruhnke TR, Reyda FB, Marques FPL. Rhinebothriidea Healy, Caira, Jensen, Webster & Littlewood, 2009 In: Caira JN, Jensen K, editors. Planetary Biodiversity Inventory (PBI): Tapeworms from vertebrate bowels of the earth (2008–2017). Kansas: University of Kansas Natural History Museum Special Publication; 2017 p. 327–348.

[10] Marques FPL, Caira JN. *Pararhinebothroides*—Neither the sister-taxon of *Rhinebothroides* nor a valid genus. J Parasitol. 2016;102: 249–259. 10.1645/15-894 26761428

[11] Aschliman NC. The Batoid tree of life: Recovering the patterns and timing of the evolution of skates, rays and allies (Chondrichthyes: Batoidea). 1st ed Tallahassee: The Florida State University; 2011.

[12] De Carvalho MR, Maisey JG, Grande L. Freshwater stingrays of the Green River formation of Wyoming (early Eocene), with the description of a new genus and species and an analysis of its phylogenetic relationships (Chondrichthyes: Myliobatiformes). Bull Am Mus Nat Hist. 2004;284: 1–136. 10.1206/0003-0090(2004)284<0001:FSOTGR>2.0.CO;2

[13] Lovejoy NR. Systematic of myliobatoid elasmobranchs: whith emphasis on phylogeny and historical biogeography of Neotropical freshwater stingrays (Potamotrigonidae: Rajiformes). Zool J Linn Soc. 1996;117: 207–257. 10.1111/j.1096-3642.1996.tb02189.x

[14] Lovejoy NR, Bermingham E, Martin AP. South American rays came in with the sea. Nature. 1998;396: 421–422.

[15] Marques FPL. Evolution of Neotropical freshwater stingrays and their parasites: Taking into account space and time. Ph.D. Thesis, University of Toronto. 2000. Avaiable from: http://www.collectionscanada.gc.ca/obj/s4/f2/dsk1/tape2/PQDD_0028/NQ50066.pdf.

[16] Naylor GJP, Caira JN, Jensen K, Rosana KAM, Straube N, Lakner C. Elasmobranch Phylogeny: A Mitochondrial Estimate Based on 595 Species In: Carrier JC, Musick JA, Heithaus MR, editors. Biology of Sharks and Their Relatives. Florida: CRC Press; 2012 p. 31–56.

[17] Lovejoy NR, Albert JS, Crampton WG. Miocene marine incursions and marine/freshwater transitions: Evidence from Neotropical fishes. J South Am Earth Sci. 2006;21: 5–13. 10.1016/j.jsames.2005.07.009

[18] Carvalho MD, Loboda TS, Silva JD. A new subfamily, Styracurinae, and new genus, *Styracura*, for *Himantura schmardae* (Werner, 1904) and *Himantura pacifica* (Beebe & Tee-Van, 1941)(Chondrichthyes: Myliobatiformes). Zootaxa. 2016; 4175: 201–221. 10.11646/zootaxa.4175.3.1 27811760

[19] Page RDM, Charleston MA. Trees within trees: Phylogeny and historical associations. Tree. 1998;13: 356–359. 21238340 10.1016/s0169-5347(98)01438-4

[20] Schindelin J, Arganda-Carreras I, Frise E, Kaynig V, Longair M, Pietzsch T, et al Fiji: an open-source platform for biological-image analysis. Nat Methods. 2012;9: 676–682. 10.1038/nmeth.2019 22743772 PMC3855844

[21] GitHub [Internet]. Vellutini BC, Marques FPL. WormBox. [cited 2014 Jan 8]. Available from: https://github.com/nelas/WormBox.

[22] R Core Team, 2014 R: A language and environment for statistical computing. Vienna: R Foundation for Statistical Computing; 2013.

[23] Clopton RE. Standard nomenclature and metrics of plane shapes for use in gregarine taxonomy. Comp Parasitol. 2004;71: 130–140. 10.1654/4151

[24] Chervy L. Unifed terminology for cestode microtriches: a proposal from the International Workshops on Cestode Systematics in 2002–2008. Folia Parasitol. 2009;56: 199–230. 10.14411/fp.2009.025 19827364

[25] Pleijel F, Jondelius U, Norlinder E, Nygren A, Oxelman B, Schander C, et al Phylogenies without roots? A plea for the use of vouchers in molecular phylogenetic studies. Mol Phylogenet Evol. 2008;48: 369–371. 10.1016/j.ympev.2008.03.024 18424089

[26] Ewing B, Green P. Base-calling of automated sequencer traces using Phred II. Error probabilities. Genome Res. 1998;8: 186–194. 9521922

[27] Ewing B, Hillier L, Wendl MC, Green P. Base-calling of automated sequencer traces using Phred I. Accuracy assessment. Genome Res. 1998;8: 175–185. 10.1101/gr.8.3.175 9521921

[28] Gordon D, Abajian C, Green P. Consed: a graphical tool for sequence finishing. Genome Res. 1998;8: 195–202. 10.1101/gr.8.3.195 9521923

[29] Gordon D, Desmarais C, Green P. Automated finishing with autofinish. Genome Res. 2001;11: 614–625. 10.1101/gr.171401 11282977 PMC311035

[30] Katoh K, Misawa K, Kuma KI, Miyata T. MAFFT: a novel method for rapid multiple sequence alignment based on fast Fourier transform. Nucleic Acids Res. 2002;30: 3059–3066. 10.1093/nar/gkf436 12136088 PMC135756

[31] Hall TA. BioEdit: a user-friendly biological sequence alignment editor and analysis program for Windows 95/98/NT. Nucleic Acids Symp. 1999;41: 95–98.

[32] Swofford D. PAUP*: Phylogenetic analysis using parsimony (* and other methods). Version 4 Massachussets: Sinauer Associates; 2002.

[33] Templeton AR, Crandall KA, Sing CF. A cladistic analysis of phenotypic association with haplotypes inferred from restriction endonuclease mapping and DNA sequence data. III. Cladogram estimation. Genetics. 1992;132: 619–635. 1385266 10.1093/genetics/132.2.619 PMC1205162

[34] Paradis E. Pegas: an R package for population genetics with an integrated-modular approach. Bioinformatics. 2010;26: 419–420. 10.1093/bioinformatics/btp696 20080509

[35] Wheeler WC. Optimization Alignment: the end of multiple sequence alignment in phylogenetics? Cladistics. 1996;12: 1–9. 10.1111/j.1096-0031.1996.tb00189.x

[36] Varón A, Vinh LS, Wheeler WC. POY version 4: phylogenetic analysis using dynamic homologies. Cladistics. 2010;26: 7285. 10.1111/j.1096-0031.2009.00282.x 34875752

[37] Wheeler WC, Aagesen L, Arango CP, Faivovich J, Grant T, D’harse C, et al Dynamic homology and phylogenetic systematics: A unified approach using POY. New York: American Museum of Natural History; 2006.

[38] Goloboff PA. Analyzing large data sets in reasonable times: solutions for composite optima. Cladistics. 1999;15: 415–428. 10.1111/j.1096-0031.1999.tb00278.x 34902941

[39] Wheeler WC. Iterative pass optimization. Cladistics. 2003;19: 254–260. 10.1111/j.1096-0031.2003.tb00368.x 12901382

[40] Wheeler WC. Implied alignment: a synapomorphy based multiple-sequence alignment method and its use in cladogram search. Cladistics. 2003;19: 261–268. 10.1111/j.1096-0031.2003.tb00369.x 12901383

[41] Goloboff PA, Farris JS, Nixon KC. TNT, a free program for phylogenetic analysis. Cladistics. 2008;24: 774–786. 10.1111/j.1096-0031.2008.00217.x

[42] Nixon KC. The parsimony ratchet, a new method for rapid parsimony analysis. Cladistics. 1999;15: 407–414. 10.1111/j.1096-0031.1999.tb00277.x 34902938

[43] Bremer K. The limits of amino acid sequence data in angiosperm phylogenetic reconstruction. Evolution. 1988;42: 795–803. 10.2307/2408870 28563878

[44] Bremer K. Branch support and tree stability. Cladistics. 1994;10: 295–304. 10.1111/j.1096-0031.1994.tb00179.x

[45] Goodman M, Olson CB, Beeber JE, Czelusniak J. New perspectives in the molecular biological analysis of mammalian phylogeny. Ann Zool Fenn. 1982;169: 19–35.

[46] Grant T, Kluge AG. Credit where credit is due: The Goodman-Bremer support metric. Mol Phylogenet Evol. 2008;49: 405–406. 10.1016/j.ympev.2008.04.023 18514548

[47] Machado DJ. YBYRÁ facilitates comparison of large phylogenetic trees. BMC Bioinformatics. 2015;16: 204 10.1186/s12859-015-0642-9 26130249 PMC4488063

[48] Darriba D, Taboada GL, Doallo R, Posada D. jModelTest 2: more models, new heuristics and parallel computing. Nat Methods. 2012;9: 772–772. 10.1038/nmeth.2109 22847109 PMC4594756

[49] Guindon S, Gascuel O. A Simple, Fast, and Accurate Algorithm to Estimate Large Phylogenies by Maximum Likelihood. Syst Biol. 2003;52: 696–704. 10.1080/10635150390235520 14530136

[50] Zwickl DJ. Genetic algorithm approaches for the phylogenetic analysis of large biological sequence datasets under the maximum likelihood criterion. Ph.D. Thesis, University of Texas at Austin. 2006. Avaiable from: https://repositories.lib.utexas.edu/bitstream/handle/2152/2666/zwickld81846.pdf?sequence=2&isAllowed=y.

[51] Lee MSY, Hugall AF. Partitioned likelihood support and the evaluation of data set conflict. Syst Biol. 2003;52: 15–22. 10.1080/10635150390132650 12554436

[52] Grant T, Kluge AG. Clade support measures and their adequacy. Cladistics. 2008;24: 1051–1064. 10.1111/j.1096-0031.2008.00231.x 34892887

[53] Meireles CM, Czelusniak J, Ferrari SF, Schneider MPC, Goodman M. Phylogenetic relationships among Brazilian howler monkeys, genus *Alouatta* (Platyrrhini, Atelidae), based on g1-globin pseudogene sequences. Genet Mol Biol. 1999;22: 337–344. 10.1590/S1415-47571999000300009

[54] Fisher RA. The use of multiple measurements in taxonomic problems. Ann Eugen. 1936;7: 179–188. 10.1111/j.1469-1809.1936.tb02137.x

[55] Rao CR. The utilization of multiple measurements in problems of biological classification. J R Stat Soc Series B Stat Methodol. 1948;10: 159–203.

[56] Swofford D. PAUP*: Phylogenetic Analysis Using Parsimony. Illinois: Illinois Natural History Survey; 1990.

[57] Euzet L, Combes C. Les problèmes de l’espèce chez les animaux parasites. In: Problèmes de l’espèce dans le règne animal. Mem Soc Zool France. 1980;3: 239–285.

[58] Brooks DR. Six new species of tetraphyllidean cestodes, including a new genus, from a marine stingray *Himantura schmardae* (Werner, 1904) from Colombia. Proc Helminthol Soc Wash. 1977;44: 51–59.

[59] Hubert N, Renno JF. Historical biogeography of South American freshwater fishes. J Biogeogr. 2006;33: 1414–1436. 10.1111/j.1365-2699.2006.01518.x

[60] Hebert PD, Ratnasingham S, deWaard JR. Barcoding animal life: cytochrome c oxidase subunit 1 divergences among closely related species. Proc Biol Sci. 2003; 270: S96–S9. 10.1098/rsbl.2003.0025 12952648 PMC1698023

[61] Lessios HA. The great American schism: Divergence of marine organisms after the rise of the Central American Isthmus. Annu Rev Ecol Evol Syst. 2008; 39: 63–91. 10.1146/annurev.ecolsys.38.091206.095815

[62] O’Dea A, Lessios HA, Coates GA, Eytan RI, Restrepo-Moreno SA, Cione AL, et al Formation of the Isthmus of Panama. Sci Adv. 2016;2: e1600883 10.1126/sciadv.1600883 27540590 PMC4988774

[63] Euzet L. Suggestions pour une nouvelle classification des cestodes tétraphyllides. In: XIV International Congress of Zoology 1956: Proceedings of the XIV International Congress of Zoology; 1953 Aug 5-12; Copenhagen: Danish Science Press; 1956. p. 347–349.

[64] Euzet L. Order Tetraphyllidea Carus, 1863 In: Khalil LF, Jones A, Bray RA, editors. Keys to the cestode parasites of vertebrates. Oxfordshire: CAB International; 1994 pp. 149–194.

[65] Leigh EG, O’Dea A, Vermeij GJ. Historical biogeography of the Isthmus of Panama. Biol Rev. 2014;89: 148–172. 10.1111/brv.12048 23869709

[66] Robertson DR, Cramer KL. Defining and dividing the Greater Caribbean: insights from the biogeography of shorefishes. PLOS One. 2014;9: e102918 10.1371/journal.pone.0102918 25054225 PMC4108436

[67] Carvalho MR, Description of two extraordinary new species of freshwater stingrays of the genus *Potamotrygon* endemic to the rio Tapajós basin, Brazil (Chondrichthyes: Potamotrygonidae), with notes on other Tapajós stingrays. Zootaxa. 2016;4167: 1–63. 10.11646/zootaxa.4167.1.1 27701358

[68] Fontenelle J.P., Carvalho M.R. Systematic revision of the *Potamotrygon scobina* Garman, 1913 species-complex (Chondrichthyes: Myliobatiformes: Potamotrygonidae), with the description of three new freshwater stingray species from Brazil and comments on their distribution and biogeography. Zootaxa. 2017;4310: 1–63. 10.11646/zootaxa.4310.1.1

[69] Coates AG, Aubry MP, Berggren WA, Collins LS, Kunk M. Early neogene history of the Central American arc from Bocas del Toro, Western Panama. Geol Soc Am Bull. 2003;115: 271–287. 10.1130/0016-7606(2003)115<0271:ENHOTC>2.0.CO;2

[70] Coates AG, McNeill DF, Aubry MP, Berggren WA, Collins LS. An introduction to the geology of the Bocas del Toro archipelago, Panama. Caribb J Sci. 2005;41: 374–391.

[71] Jordan DS. The law of geminate species. Am Nat. 1908;42: 73–80. 10.1086/278905

[72] Trevisan B., Marques F.P.L. Species diversity of *Rhinebothrium* Linton, 1890 (Eucestoda: Rhinebothriidea) from *Styracura*, including the description of a new species. Zootaxa. 2017;4300: 421–437. 10.11646/zootaxa.4300.3.5

[73] Ekman S. Zoogeography of the sea. London: Sidgwick & Jackson; 1953.

[74] Agrawal N, Agarwal GG, Tripathi P, Pant R. Discriminant analysis: A supportive tool for monogenoidean taxonomy. Biosci Trends. 2008;2: 128–132. 20103916

[75] Hanzelová V, Kuchta R, Scholz T, Shinn AP. Morphometric analysis of four species of *Eubothrium* (Cestoda: Pseudophyllidea) parasites of salmonid fish: an interspecific and intraspecific comparison. Parasitol Int. 2005;54: 207–214. 10.1016/j.parint.2005.05.001 15979933

[76] Jaiswara R, Nandi D, Balakrishnan R. Examining the effectiveness of discriminant function analysis and cluster analysis in species identification of male field crickets based on their calling songs. PLOS One. 2013;8: e75930 10.1371/journal.pone.0075930 24086666 PMC3783383

[77] Parsons S, Jones G. Acoustic identification of twelve species of echolocating bat by discriminant function analysis and artificial neural networks. J Exp Biol. 2000;203: 2641–2656. 10934005 10.1242/jeb.203.17.2641

[78] Clouse RM, Wheeler WC. Descriptions of two new, cryptic species of *Metasiro* (Arachnida: Opiliones: Cyphophthalmi: Neogoveidae) from South Carolina, USA, including a discussion of mitochondrial mutation rates. Zootaxa. 2014;3814: 177–201. 10.11646/zootaxa.3814.2.2 24943422

[79] Leliaert F, Verbruggen H, Vanormelingen P, Steen F, López-Bautista JM, Zuccarello GC, et al DNA-based species delimitation in algae. Eur J Phycol. 2014;49: 179–196. 10.1080/09670262.2014.904524

[80] Dayrat B. Towards integrative taxonomy. Biol J Linn Soc. 2005;85: 407–415. 10.1111/j.1095-8312.2005.00503.x

[81] Padial JM, Miralles A, De-la-Riva I, Vences M. The integrative future of taxonomy. Front Zool. 2010;7: 16 10.1186/1742-9994-7-16 20500846 PMC2890416

[82] Pérez-Ponce de León G, Nadler SA. What we don’t recognize can hurt us: a plea for awareness about cryptic species. J Parasitol. 2010;96: 453–464. 10.1645/GE-2260.1 19925040

[83] Pérez-Ponce de León G, poulin R. An updated look at the uneven distribution of cryptic diversity among parasitic helminths. J Helminthol. 2017;6: 1–6. 10.1017/S0022149X17000189 28260533

[84] Knowlton N. Sibling species in the sea. Annu Rev Ecol Evol Syst. 1993;24: 189–216. 10.1146/annurev.es.24.110193.001201

[85] Lajus D, Sukhikh N, Alekseev V. Cryptic or pseudocryptic: can morphological methods inform copepod taxonomy? An analysis of publications and a case study of the *Eurytemora affinis* species complex. Ecol Evol. 2015;5: 2374–2385. 10.1002/ece3.1521 26120427 PMC4475370

[86] Caira JN. Synergy advances parasite taxonomy and systematics: an example from elasmobranch tapeworms. Parasitology. 2011;138: 1675–1687. 10.1017/S0031182011000643 21729352

[87] Bickford D, Lohman DJ, Navjot SS, Ng PKL, Meier R, Winker K, et al Cryptic species as a window on diversity and conservation. Trends Ecol Evol. 2007;22: 148–155. 10.1016/j.tree.2006.11.004 17129636

[88] Pfenninger M, Schwenk K. Cryptic animal species are homogeneously distributed among taxa and biogeographical regions. BMC Evol Biol. 2007;7: 121 10.1186/1471-2148-7-121 17640383 PMC1939701

[89] Trontelj P, Fišer C. Perspectives: cryptic species diversity should not be trivialised. Syst Biodivers. 2009;7: 1–3. 10.1017/S1477200008002909

[90] Jörger KM, Schrödl M. How to describe a cryptic species? Practical challenges of molecular taxonomy. Front Zool. 2013;10: 59 10.1186/1742-9994-10-59 24073641 PMC4015967

[91] Karanovic T, Djurakic M, Eberhard SM. Cryptic species or inadequate taxonomy? Implementation of 2D geometric morphometrics based on integumental organs as landmarks for delimitation and description of copepod taxa. Syst Biology. 2016;65: 304–327. 10.1093/sysbio/syv088 26608965

[92] Morard R, Quillévéré F, Escarguel G, de Garidel-Thoron T, de Vargas C, Kucera M. Ecological modeling of the temperature dependence of cryptic species of planktonic Foraminifera in the Southern Hemisphere. Palaeogeogr, Palaeoclimatol, Palaeoecol. 2013;391: 13–33. 10.1016/j.palaeo.2013.05.011

[93] Fontaneto D, Kaya M, Herniou EA, Barraclough TG. Extreme levels of hidden diversity in microscopic animals (Rotifera) revealed by DNA taxonomy. Mol Phylogenet Evol. 2009;53: 182–189 10.1016/j.ympev.2009.04.011 19398026

[94] Fouquet A, Vences M, Salducci MD, Meyer A, Marty C, Blanc M, et al Revealing cryptic diversity using molecular phylogenetics and phylogeography in frogs of the *Scinax ruber* and *Rhinella margaritifera* species groups. Mol Phylogenet Evol. 2007;43: 567–582. 10.1016/j.ympev.2006.12.006 17303441

[95] Janzen DH, Hallwachs W, Blandin P, Burns JM, Cadiou J, Chacon I, et al Integration of DNA barcoding into an ongoing inventory of complex tropical biodiversity. Mol Ecol Res. 2009;9: S1–S26. 10.1111/j.1755-0998.2009.02628.x 21564960

[96] Lahaye R, Van der Bank M, Bogarin D, Warner J, Pupulin F, Gigot G, et al DNA barcoding the floras of biodiversity hotspots. Proc Nati Acad Sci. 2008;105: 2923–2928. 10.1073/pnas.0709936105 PMC2268561 18258745

[97] Vieites DR, Wollenberg KC, Andreone F, Köhler J, Glaw F, Vences M. Vast underestimation of Madagascar’s biodiversity evidenced by an integrative amphibian inventory. Proc Nati Acad Sci. 2009;106: 8267–8272. 10.1073/pnas.0810821106 PMC2688882 19416818

[98] Mayer F, Dietz C, Kiefer A. Molecular species identification boosts bat diversity. Front Zool. 2007;4: 4 10.1186/1742-9994-4-4 17295921 PMC1802075

[99] Caira JN, Jensen K. An investigation of the coevolutionary relationships between onchobothriid tapeworms and their elasmobranch hosts. Int J Parasitol. 2001;31: 960–975. 10.1016/S0020-7519(01)00206-5 11406144

[100] Goldstein PZ, DeSalle R. Integrating DNA barcode data and taxonomic practice: determination, discovery, and description. BioEssays. 2010;33: 135–147. 10.1002/bies.201000036 21184470

[101] Caira, JN, Jensen K, Barbeau E. Editors. Global Cestode Database; 2016 [cited 2016 Dec 12]. Database: Uconn [Internet]. Avaiable from: www.tapewormdb.uconn.edu.

[102] Kuchta R, Scholz T. *Australicola pectinatus* n. gen. and sp. n. (Cestoda: Pseudophyllidea) from deep-sea fish *Beryx splendens* from Tasmania. J Parasitol. 2006;92: 126–129. 10.1645/GE-3511.1 16629325

[103] Kuchta R, Scholz T. A new triaenophorid tapeworm from blackfish *Centrolophus niger*. J Parasitol. 2008;94: 500–504. 10.1645/GE-1425.1 18564751

[104] Randhawa HS, Saunders GW, Scott ME, Burt MDB. Redescription of *Pseudanthobothrium hanseni* Baer, 1956 and description of *P. purtoni* sp. n.(Cestoda: Tetraphyllidea) from different pairs of rajid skate hosts, with comments on the host-specificity of the genus in the northwest Atlantic. Syst Parasitol. 2008;70: 41–60. 10.1007/s11230-007-9122-6 18373219

[105] De Chambrier A, Scholz T, Beletew M, Mariaux J. A new genus and species of proteocephalidean (Cestoda) from *Clarias catfishes* (Siluriformes: Clariidae) in Africa. J Parasitol. 2009;95: 160–168. 10.1645/GE-1594.1 18576867

[106] Fyler CA, Caira JN, Jensen K. Five new species of *Acanthobothrium* (Cestoda: Tetraphyllidea) from an unusual species of *Himantura* (Rajiformes: Dasyatidae) from northern Australia. Folia Parasitol. 2009;56: 107–128. 10.14411/fp.2009.016 19606787

[107] Fyler CA, Caira JN. Phylogenetic status of four new species of *Acanthobothrium* (Cestoda: Tetraphyllidea) parasitic on the wedgefish *Rhynchobatus laevis* (Elasmobranchii: Rhynchobatidae): implications for interpreting host associations. Invertebr Syst. 2010;24: 419–433. 10.1071/IS10034

[108] Haukisalmi V, Hardman LM, Foronda P, Feliu C, Laakkonen J, Niemimaa J, et al Systematic relationships of hymenolepidid cestodes of rodents and shrews inferred from sequences of 28S ribosomal RNA. Zool Scr. 2010;39: 631–641. 10.1111/j.1463-6409.2010.00444.x

[109] Cutmore SC, Theiss SM, Bennett MB, Cribb TH. A new phyllobothriid genus and species from the snaggletooth shark, *Hemipristis elongata* (Carcharhiniformes: Hemigaleidae), from Moreton Bay, Australia. Folia Parasitol. 2011;58: 187–196. 10.14411/fp.2011.019 22053616

[110] Fyler CA. An extremely hyperapolytic *Acanthobothrium species* (Cestoda: Tetraphyllidea) from the Japanese wobbegong, *Orectolobus japonicus* (Elasmobranchii: Orectolobiformes) in Taiwan. Comp Parasitol. 2011;78: 4–14. 10.1654/4454.1

[111] Melo FTDV, Giese EG, Furtado AP, Soares MJ, Gonçalves EC, Vallinoto ACR, Santos JND. *Lanfrediella amphicirrus* gen. n. sp. n. Nematotaeniidae (Cestoda: Cyclophyllidea), a tapeworm parasite of *Rhinella marina* (Linnaeus, 1758)(Amphibia: Bufonidae). Mem Inst Oswaldo Cruz. 2011;106: 670–677. 10.1590/S0074-02762011000600005 22012220

[112] Reyda FB, Marques FPL. Diversification and species boundaries of *Rhinebothrium* (Cestoda; Rhinebothriidea) in South American freshwater stingrays (Batoidea; Potamotrygonidae). PLOS One. 2011;6: e22604 10.1371/journal.pone.0022604 21857936 PMC3153936

[113] Schaeffner BC, Gasser RB, Beveridge I. *Ancipirhynchus afossalis* n. g., sp. n. (Trypanorhyncha: Otobothriidae), from two species of sharks off Indonesian and Malaysian Borneo. Syst Parasitol. 2011;80: 1–15. 10.1007/s11230-011-9309-8 21805386

[114] Greiman SE, Tkach VV. Description and phylogenetic relationships of *Rodentolepis gnoskei* sp. n. (Cyclophyllidea: Hymenolepididae) from a shrew *Suncus varilla* minor in Malawi. Parasitol Int. 2012;61: 343–350. 10.1016/j.parint.2012.01.003 22265723

[115] Kuchta R, Burianová A, Jirků M, de Chambrier A, Oros M, Brabec J, et al Bothriocephalidean tapeworms (Cestoda) of freshwater fish in Africa, including erection of *Kirstenella* n. g. and description of *Tetracampos martinae* sp. n. Zootaxa. 2012;3309: 1–35.

[116] Greiman SE, Tkach VV, Cook JA. Description of molecular differentiation of a new *Staphylocystoides* (Cyclophyllidea: Hymenolepididae) from the dusky shrew *Sorex monticolus* in Southeast Alaska. J Parasitol. 2013;99: 1045–1049. 10.1645/13-302.1 23919726

[117] Ruhnke TR, Workman RE. Two new species and a new phyllobothriid cestode genus from sharks of the genus *Negaprion Whitley* (Carcharhiniformes). Syst Parasitol. 2013;85: 37–48. 10.1007/s11230-013-9411-1 23595490

[118] Tkach VV, Makarikov AA, Kinsella JM. Morphological and molecular differentiation of *Staphylocystis clydesengeri* sp. n.(Cestoda, Hymenolepididae) from the vagrant shrew *Sorex vagrans* (Soricimorpha, Soricidae) in North America. Zootaxa. 2013;3691: 389–400. 26167593 10.11646/zootaxa.3691.3.7

[119] Haukisalmi V, Hardman LM, Hoberg EP, Henttonen H. Phylogenetic relationships and taxonomic revision of *Paranoplocephala* Lühe, 1910 sensu lato (Cestoda, Cyclophyllidea, Anoplocephalidae). Zootaxa. 2014;3873: 371–415 10.11646/zootaxa.3873.4.3 25544228

[120] Phillips AJ, Georgiev BB, Waeschenbach A, Mariaux J. Two new and two redescribed species of *Anonchotaenia* (Cestoda: Paruterinidae) from South American birds. Folia Parasitol. 2014;61: 441–461. 25549500

[121] Bernot JP, Caira JN, Pickering M. The dismantling of *Calliobothrium* (Cestoda: Tetraphyllidea) with erection of *Symcallio* n. gen. and description of two new species. J Parasitol. 2015;101: 167–181. 10.1645/14-571.1 25506725

[122] Binkienė R, Kornienko SA, Tkach VV. *Soricinia genovi* sp. n. from *Neomys fodiens* in Bulgaria, with redescription of *Soricinia globosa* (Baer, 1931) (Cyclophyllidea: Hymenolepididae). Parasitol Res. 2015;114: 209–218 10.1007/s00436-014-4180-6 25342463

[123] Makarikov AA, Nims TN, Galbreath KE, Hoberg EP. *Hymenolepis folkertsi* sp. n.(Eucestoda: Hymenolepididae) in the oldfield mouse *Peromyscus polionotus* (Wagner) (Rodentia: Cricetidae: Neotominae) from the southeastern Nearctic with comments on tapeworm faunal diversity among deer mice. Parasitol Res. 2015;114: 2107–2117. 10.1007/s00436-015-4399-x 25762188

[124] Reyda FB, Healy CJ, Haslach AR, Ruhnke TR, Aprill TL, Bergman MP, et al A new genus of rhinebothriidean cestodes from batoid elasmobranchs, with the description of five new species and transfer of two species. Folia Parasitol. 2016;63: 038 10.14411/fp.2016.038 27973339

[125] Chilton NB, O’Callaghan MG, Beveridge I, Andrews RH. Genetic markers to distinguish *Moniezia expansa* from *M-benedeni* (Cestoda: Anoplocephalidae) and evidence of the existence of cryptic species in Australia. Parasitol Res. 2007;100: 1187–1192. 10.1007/s00436-006-0388-4 17206509

[126] Jia WZ, Yan HB, Lou ZZ, Ni XW, Dyachenko V, Li HM, et al Mitochondrial genes and genomes support a cryptic species of tapeworm within *Taenia taeniaeformis*. Acta Trop. 2012;123: 154–163. 10.1016/j.actatropica.2012.04.006 22569565

[127] Makarikov AA, Galbreath KE, Hoberg EP. Parasite diversity at the Holarctic nexus: species of *Arostrilepis* (Eucestoda: Hymenolepididae) in voles and lemmings (Cricetidae: Arvicolinae) from greater Beringia. Zootaxa. 2013;3608: 401–439. 10.11646/zootaxa.3608.6.1 24614481

[128] Nakao M, Lavikainen A, Iwaki T, Haukisalmi V, Konyaev S, Oku Y, et al Molecular phylogeny of the genus Taenia (Cestoda: Taeniidae): Proposals for the resurrection of *Hydatigera* Lamarck, 1816 and the creation of a new genus Versteria. Int J Parasitol. 2013;43: 427–437. 10.1016/j.ijpara.2012.11.014 23428901

[129] Beveridge I, Gasser RB. Diversity in parasitic helminths of Australasian marsupials and monotremes: a molecular perspective. Int J Parasitol. 2014;44: 859–864. 10.1016/j.ijpara.2014.06.001 24992656

[130] Scholz T, Oros M, Bazsalovicsova E, Brabec J, Waeschenbach A, Xi BW, et al Molecular evidence of cryptic diversity in *Paracaryophyllaeus* (Cestoda: Caryophyllidea), parasites of loaches (Cobitidae) in Eurasia, including description of *P-vladkae* sp. n. Parasitol Int. 2014;63: 841–850. 10.1016/j.parint.2014.07.015 25111941

[131] Lavikainen A, Iwaki T, Haukisalmi V, Konyaev SV, Casiraghi M, Dokuchaev NE, et al Reappraisal of *Hydatigera taeniaeformis* (Batsch, 1786) (Cestoda: Taeniidae) sensu lato with description of *Hydatigera kamiyai* sp. n. Int J Parasitol. 2016;46: 361–374. 10.1016/j.ijpara.2016.01.009 26956060

[132] Guo AJ. *Moniezia benedeni* and *Moniezia expansa* are distinct cestode species based on complete mitochondrial genomes. Acta Trop. 2017;166: 287–292 10.1016/j.actatropica.2016.11.032 27923556

[133] Marques JF, Santos MJ, Gibson DI, Cabral HN, Olson PD. Cryptic species of *Didymobothrium rudolphii* (Cestoda: Spathebothriidea) from the sand sole, *Solea lascaris*, off the Portuguese coast, with an analysis of their molecules, morphology, ultrastructure and phylogeny. Parasitol. 2007;34: 1057–1072. 10.1017/S0031182007002491 17326848

[134] Renner SS. A return to Linnaeus’s focus on diagnosis, not description: the use of DNA characters in the formal naming of species. Syst Biol. 2016;65: 1085–1095. 10.1093/sysbio/syw032 27146045

[135] Cook LG, Edwards RD, Crisp MD, Hardy NB. Need morphology always be required for new species descriptions? Invertebr Syst. 2010;24: 322–326.

[136] Morard R, Escarguel G, Weiner AKM, André A, Douady CJ, Wade CM, et al Nomenclature for the nameless: A proposal for an integrative molecular taxonomy of cryptic diversity exemplified by planktonic foraminifera. Syst Biol. 2016;65: 925–940. 10.1093/sysbio/syw031 27073250

[137] Esselstyn JA. Should universal guidelines be applied to taxonomic research? Biol J L Soc. 2007;90: 761–764. 10.1111/j.1095-8312.2007.00776.x

[138] Pante E, Puillandre N, Viricel A, Arnaud-Haond S, Aurelle D, Castelin M, et al Species are hypotheses: avoid connectivity assessments based on pillars of sand. Mol Ecol. 2015;24: 525–544. 10.1111/mec.13048 25529046

