# Supplementary figures and images for: Systematics and diversification of Anindobothrium Marques, Brooks & Lasso, 2001 (Eucestoda: Rhinebothriidea)
Source: PLoS One. 2017 Sep 27;12(9):e0184632. doi: 10.1371/journal.pone.0184632 (PMC5617167; doi:10.1371/journal.pone.0184632)

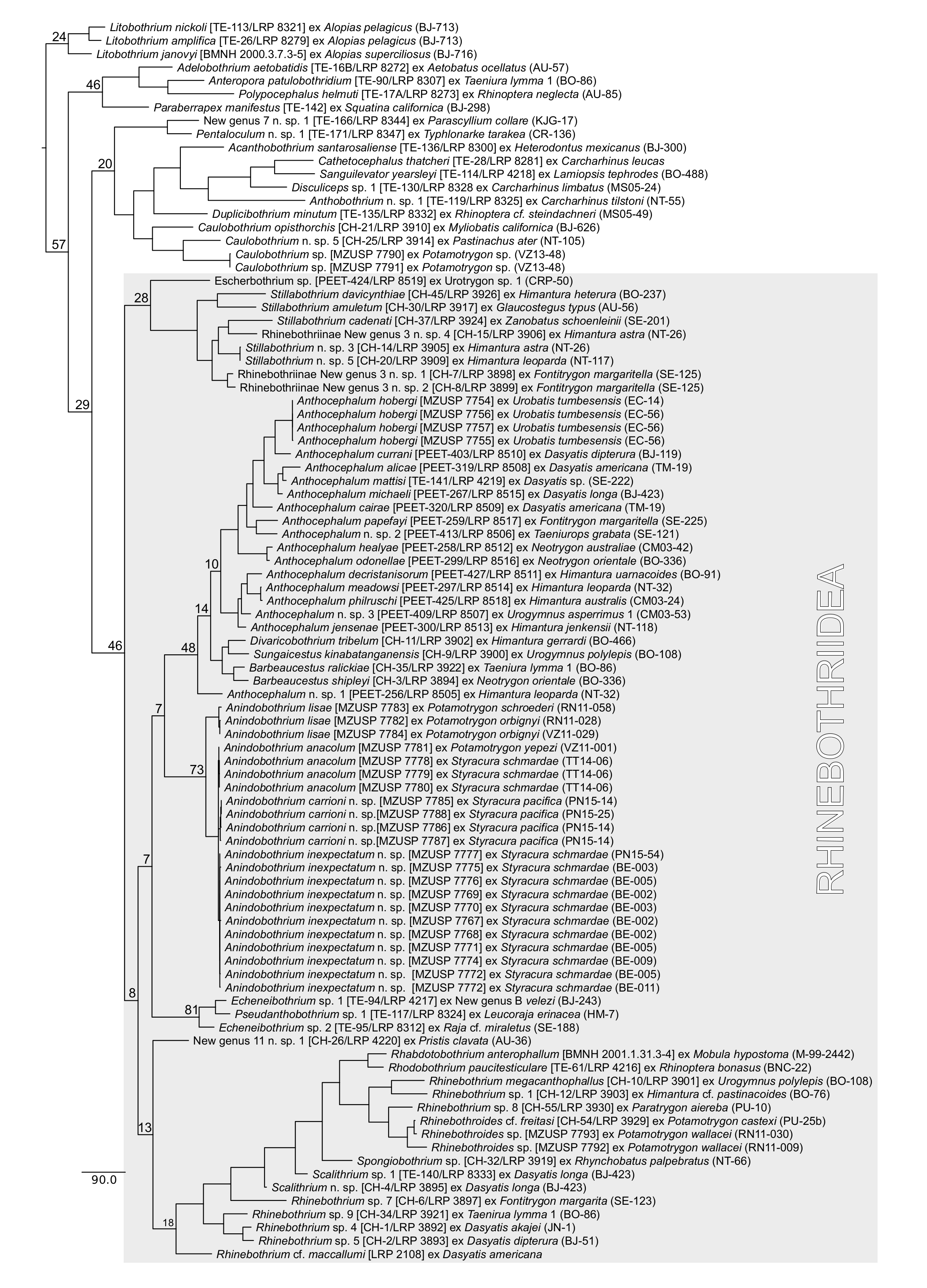

Supplement: S1 Fig — (TIF) [file pone.0184632.s002.tif]

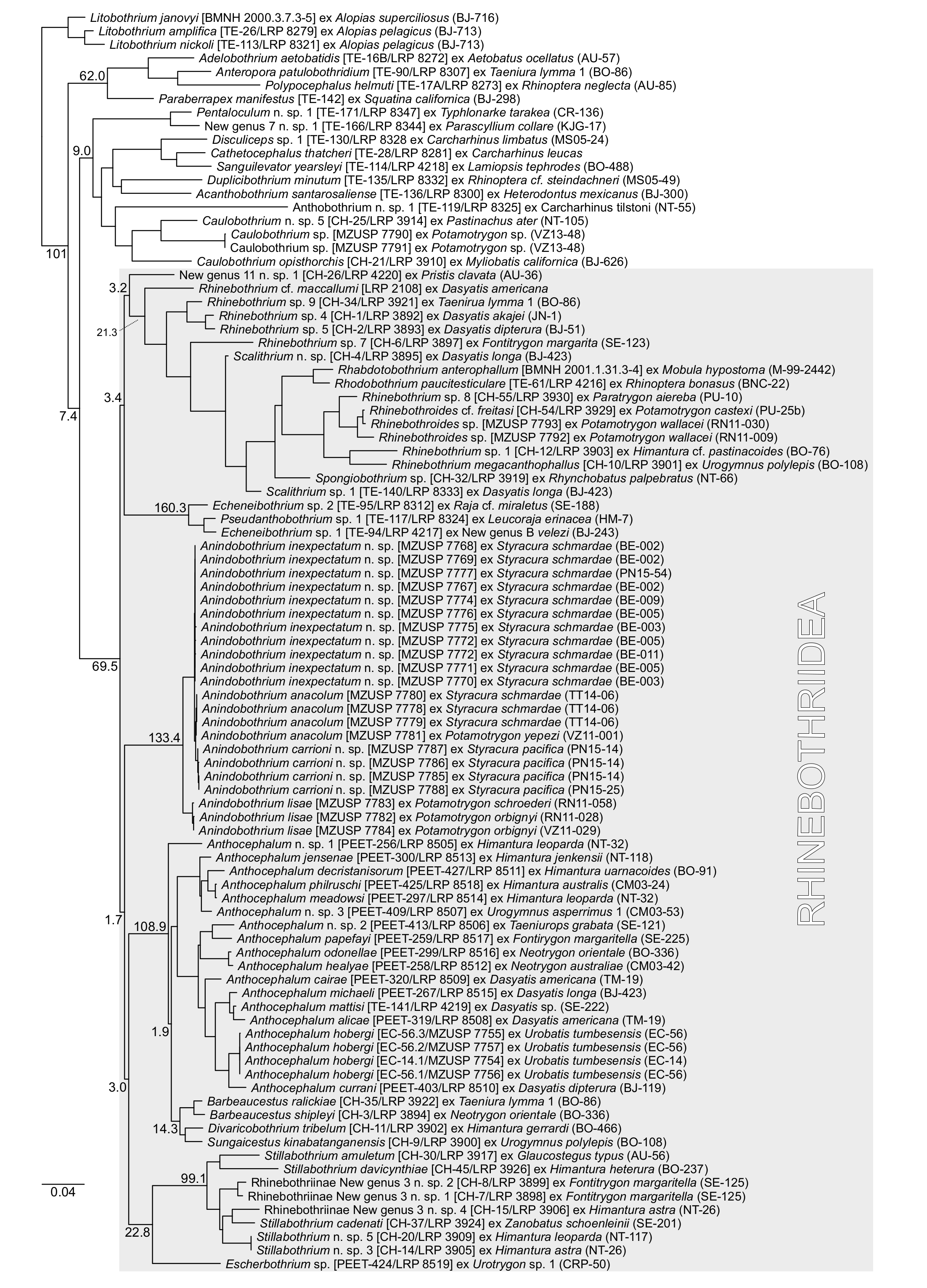

Supplement: S2 Fig — (TIF) [file pone.0184632.s003.tif]
